# Acceptor range of endo-β-*N*-acetylglucosaminidase mutant endo-CC N180H: from monosaccharide to antibody

**DOI:** 10.1098/rsos.171521

**Published:** 2018-05-16

**Authors:** Shino Manabe, Yoshiki Yamaguchi, Junpei Abe, Kana Matsumoto, Yukishige Ito

**Affiliations:** 1 Synthetic Cellular Chemistry Laboratory, RIKEN, Hirosawa, Wako, Saitama 351-0198, Japan; 2 Structural Glycobiology Team, RIKEN, Hirosawa, Wako, Saitama 351-0198, Japan

**Keywords:** endo-β-*N*-acetylglucosaminidase mutant, endo-CC N180H, glycan transfer, antibody, sialylglycopeptide

## Abstract

The endo-β-*N*-acetylglucosaminidase mutant endo-CC N180H transfers glycan from sialylglycopeptide (SGP) to various acceptors. The scope and limitations of low-molecular-weight acceptors were investigated. Several homogeneous glycan-containing compounds, especially those with potentially useful labels or functional moieties, and possible reagents in glycoscience were synthesized. The 1,3-diol structure is important in acceptor molecules in glycan transfer reactions mediated by endo-CC N180H as well as by endo-M-N175Q. Glycan remodelling of antibodies was explored using core-fucose-deficient anti-CCR4 antibody with SGP and endo-CC N180H. Homogeneity of the glycan in the antibody was confirmed by mass spectrometry without glycan cleavage.

## 1. Introduction

**Scheme 1.**
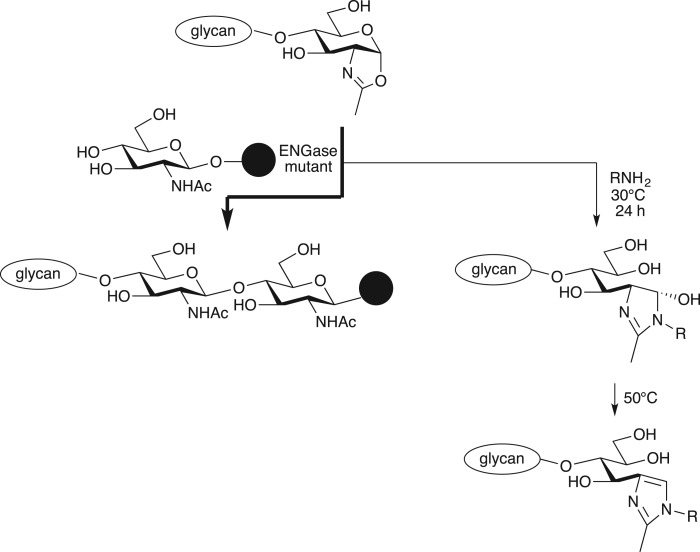
Glycan transfer and side reactions between oxazolines and acceptors mediated by ENGases.

Endo-β-*N*-acetylglucosaminidases (ENGases) are glycosidic hydrolases that act on the β-1,4-glycosidic linkage within the *N,N′*-diacetylchitobiose core of *N*-glycans. Several ENGases, such as Endo-H from *Streptomyces plicatus* [1], Endo-A from *Anthrobacter protophormiae* [2] and Endo-M from *Mucor hiemalis* [3], have been isolated [4–9]. ENGase mutants belonging to the glycosyl hydrolase family 85 transfer glycosides *en bloc* from donor glycans to a variety of glycosyl acceptors containing *N*-acetylglucosamine (GlcNAc), as shown in scheme 1 [10,11]. ENGases are widely employed in synthetic applications that need homogeneous glycosides of glycopeptides, glycoproteins, and glycoconjugates [12–16]. Glycan remodelling of therapeutic monoclonal antibodies is especially important as glycan structure influences antibody effector functions, stability, and pharmacokinetics/pharmacodynamics [17,18]. These strategies employ elegant transition-state oxazoline-mimics as donors together with various ENGases [19–22]. However, it has recently been reported that the oxazoline sugar undergoes side reactions if conditions are not strictly controlled, because a highly reactive amino group can attack the carbon between the nitrogen and oxygen of oxazoline [23–25]. If a complex, high-molecular-weight protein like an antibody is used as acceptor, it is important that side reactions be avoided, as this could make purification more difficult and, more importantly, modify function in unexpected ways. Although it is reported that side reactions are suppressed when more enzyme and less oxazoline are used [23], an alternative approach to solve the problem could be choice of enzyme and glycosyl donor. Recently, glycosynthase mutants of endo-S2, endo-S2 D184M and endo-S2 D184Q, were reported [26,27]. These enzymes have potent transglycosylation activity with minimal side reactions. We expect that we can also minimize side reactions when we avoid oxazoline as a donor.

Endo-CC is an ENGase extracted from *Coprinopsis cinerea* [28], and endo-CC N180H, an endo-CC mutant, transfers glycan to RNase B [29]. Endo-CC N180H has several advantages over other ENGase mutants: it can be prepared easily and in high quantities from *E. coli* cell culture, it is thermally stable (survives 50°C for 10 min), and has an optimum pH of 7.5. We anticipated that endo-CCN180H may be a useful glycan transfer enzyme, but its specificity has not been well characterized. Here, we report the glycan transfer activity of endo-CC N180H from sialylglycopeptide (SGP) **1** [30] to various GlcNAc-containing potential substrates such as monosaccharides, glycopeptides and a deglycosylated antibody.

## 2. Material and methods

### 2.1. General methods

All commercial reagents were used without further purification. Analytical thin-layer chromatography was performed on silica gel 60 F254 plates (Merck) and visualized by UV fluorescence quenching and 12 molybdo(VI) phosphoric acid /phosphoric acid /sulfuric acid staining. Flash column chromatography was performed on silica gel 60N (spherical, neutral, 40–100 µm, Kanto Chemical Co., Inc.). Yields reported here are isolated yields. ^1^H- and ^13^C-NMR spectra of acceptors were recorded with a JEOL AL 400 spectrometer (400 and 100 MHz, respectively) at ambient temperatures (23–24°C) in CDCl_3_, CD_3_OD. Chemical shifts (*δ*) are reported in ppm relative to internal tetramethylsilane (*δ* = 0.00 ppm) in CDCl_3_, or remaining solvent peak (*δ* = 3.30 ppm for CD_3_OD) for ^1^H-NMR spectra. ^13^C-NMR chemical shifts (*δ*) are reported in ppm relative to remaining solvent peak CDCl_3_ (*δ* = 77.00 ppm), CD_3_OD (*δ* = 49.00 ppm). NMR spectra of compounds **3**, **4b**, **5b**, **6b**, **7b**, **8b**, **9b**, and **15b** were recorded with a 600 MHz NMR spectrometer (Bruker BioSpin) equipped with a 5-mm TXI probe. The probe temperature was set to 25°C. The samples were dissolved in D_2_O, and pH was adjusted to 7 with NaOD or DCl. ^1^H-NMR chemical shifts were reported relative to the internal standard 4,4-dimethyl-4-silapentane-1-sulfonic acid (DSS, methyl peak as 0 ppm). The ^13^C-NMR chemical shifts were reported using the external DSS. NMR signals from the ^13^C-labelled glucose residue were assigned by 1D ^1^H, 2D ^1^H-^13^C heteronuclear single quantum coherence (HSQC) and 2D HCCH-COSY spectra. 2D ^1^H-^13^C heteronuclear multiple bond correlation (HMBC) spectrum was collected for the confirmation of linkage between GlcNAc-2 and Glc-1. For the linkage analysis of compound **9b**, 1D ^1^H, 1D-selective TOCSY, 2D ^1^H-^1^H DQF-COSY, 2D ^1^H-^13^C HSQC and 2D ^1^H-^13^C HMBC spectra were used. To estimate the coupling constants (^1^*J*_CH_), ^13^C-coupled 2D ^1^H-^13^C HSQC spectrum was collected with the final digital resolution of 1.8 Hz/point at the ^1^H-dimension. The NMR data were processed with TopSpin (v. 2.1), and the spectra were displayed using XWIN-PLOT. High-resolution mass spectrometry (HRMS) was conducted with a hybrid quadrupole-TOF tandem mass spectrometer (Synapt G2, Waters). Electrospray ionization mass spectrometry (ESI-MS) analysis of the antibody product was performed using a QSTAR ELITE quadrupole-time-of-flight mass spectrometer (AB Sciex, Foster City, CA) equipped with a Nanospray Tip (Humanix, Hiroshima, Japan). Optical rotations were measured at room temperature (JASCO DIP-310). High-performance liquid chromatography (HPLC; Prominence, Shimadzu, Kyoto, Japan) was used for purification of low-molecular-weight compounds and analyses of time-course of enzymatic reaction. Ultra-performance liquid chromatography (UPLC; ACQUITY, UPLC H-Class System, Waters) was used for reaction analyses of antibody-glycan remodelling.

Compounds **6a**, **7a**, **8a**, **9a**, **10a**, **11a**, and **12a** were purchased from Sigma-Aldrich. D-[UL-^13^C_6_] glucose was from CIL. Mightysil RP-18 GP (20 × 250** **mm for preparative scale; 4.6 × 250** **mm for analytical scale) was purchased from Kanto Chemical Co., Inc. Endo-CC N180H was available from Fushimi Pharmaceutical Co. Ltd (Marugame, Japan). Endo-M-N175Q was available from TCI (Tokyo). SGP **1** was available from Fushimi Pharmaceutical Co. Ltd and TCI. One unit of endo-CC N180H is defined as the amount of enzyme that produces 1 µmol SG-GlcNAc-*p*NP from *p*NP-GlcNAc per minute at 30°C, pH 7.5. One unit of endo-M-N175Q is defined as the amount of enzyme that produces 1 µmol SG-GlcNAc-*p*NP from *p*NP-GlcNAc per minute at 30°C, pH 7.0.

### 2.2. General procedure for glycan transfer to low-molecular-weight acceptors by endo-CC N180H

A solution of SGP (9 or 30** **mM) and the glycosyl acceptor (3** **mM) were incubated with endo-CC N180H (1.3 or 2.6 or 6.4 mU) in 50 µl of Tris/HCl buffer (20** **mM, pH 7.5) containing DMSO (5 µl) at 30°C or 40°C. For the HPLC analysis at the desired time point (0** **min, 15** **min, 30** **min, 1** **h, 3** **h, 6** **h, 12** **h, 24** **h and 48** **h), a part of the reaction mixture (5 µl) was heated at 100°C for 3** **min to denature the enzyme and quench the reaction and the contents analysed by reversed phase HPLC. Analytical HPLC was performed using a C18 reverse phase column (Mightysil RP-18 GP, Kanto Chemical Co., Inc., Tokyo) with 0% MeCN for 10** **min and then a linear gradient of 0–100% MeCN in 0.1% aqueous trifluoroacetic acid (TFA) over 40** **min at room temperature at a flow rate of 1 ml min^−1^, detected at 214, and 254 or 280 or 301** **nm. The latter three wavelengths were used to determine the HPLC yield by calculating the ratio among the sum of peak areas for each transglycosylation product and the sum of peak areas of all the detected peaks.

**Compound 3.** Selected data of^1^H-NMR (D_2_O) *δ* 8.25 (d, *J* = 9.3 Hz, 2H), 7.17 (d, *J* = 9.3 Hz, 2H), 5.31 (d, *J* = 8.4 Hz, 1H), 5.13 (s, 1H), 4.95 (s, 1H), 4.62–4.60 (m, 4H), 4.44 (dd, *J* = 7.8 Hz, 3.0 Hz, 2H), 4.23 (s, 1H), 4.19 (s, 1H), 4.15 (s, 1H), 4.06 (t, *J* = 10.2 Hz, 1H), 2.67 (td, *J* = 12.0 Hz, 4.0 Hz, 2H), 2.10 (s, 3H), 2.07 (s, 3H), 2.06 (s, 3H), 2.05 (s, 6H), 2.02 (s, 3H), 1.72 (t, *J* = 12.6 Hz, 2H); ^13^C-NMR (D_2_O) *δ* 177.62, 177.43, 177.38, 176.24, 164.34, 145.39, 128.79, 119.17, 106.24, 104.11, 103.17, 102.87, 102. 84, 102.27, 102.03, 101.95, 101.13, 99.64, 83.40, 83.33, 83.19, 82.33, 81.56, 79.09, 78.90, 77.58, 77.12, 77.08, 77.04, 76.39, 74.24, 75.22, 75.11, 74.85, 74.81, 74.75, 74.67, 74.41, 73.42, 72.89, 72.17, 72.12, 71.09, 71.05, 70.90, 70.03, 69.99, 68.57, 68.38, 66.02, 65.34, 64.41, 64.33, 62.92, 62.65, 62.52, 57.62, 57.39, 57.32, 54.57, 25.11, 24.96, 24.74.

**Compound 4b.** Selected data of^1^H-NMR (D_2_O) *δ* 8.25 (d, *J* = 9.0 Hz, 2H), 7.17 (d, *J* = 9.0 Hz, 2H), 5.31 (d, *J* = 8.4 Hz, 1H), 5.13 (s, 1H), 4.95 (s, 1H), 4.64–4.60 (m, 4H), 4.45 (dd, *J* = 7.8 Hz, 3.0 Hz, 2H), 4.26 (s, 1H), 4.19 (s, 1H), 4.16 (s, 1H), 4.06 (dt, *J* = 10.8 Hz, 3.6 Hz, 1H), 2.67 (dt, *J* = 12.4 Hz, 4.4 Hz, 2H), 2.10 (s, 3H), 2.07 (s, 3H), 2.06 (s, 3H), 2.03 (s, 3H), 2.01 (s, 3H), 2.00 (s, 3H), 1.72 (t, *J* = 12.0 Hz, 2H); ^13^C-NMR (D_2_O) *δ* 180.51, 177.62, 177.43, 177.15, 176.24, 164.34, 145.38, 128.79, 119.17, 106.24, 104.11, 103.17, 102.87, 102.27, 102.03, 101.96, 101.12, 99.64, 81.41, 83.33, 83.19, 81.56, 79.09, 78.90, 77.58, 77.12, 77.08, 76.39, 76.24, 75.53, 75.24, 75.11, 74.85, 74.75, 74.67, 74.41, 73.42, 72.89, 72.17, 72.12, 71.09, 71.05, 70.91, 70.03, 69.98, 68.58, 68.39, 66.02, 65.35, 64.41, 62.93, 62.65, 62.53, 57.62, 57.39, 57.32, 54.57, 42.76, 25.12, 24.96, 24.91, 24.74, 24.58.

**Compound 5b.** Selected data of^1^H-NMR (D_2_O) *δ* 8.25 (d, *J* = 9.0 Hz, 2H), 7.18 (d, *J* = 9.0 Hz, 2H), 5.37 (d, *J* = 8.4 Hz, 1H), 5.13 (s, 1H), 4.95 (s, 1H), 4.65–4.60 (m, 4H), 4.45 (dd, *J* = 7.8 Hz, 2.4 Hz, 2H), 4.26 (s, 1H), 4.19 (s, 1H), 2.68 (dt, *J* = 12.4 Hz, 4.2 Hz, 2H), 2.07 (s, 3H), 2.06 (s, 3H), 2.03 (s), 1.72 (t, *J* = 12.2 Hz, 2H); ^13^C-NMR (D_2_O) *δ* 177.60, 177.43, 176.24, 164.25, 145.43, 128.82, 119.16, 106.26, 104.12, 103.17, 102.87, 102.83, 102.27, 102.03, 101.95, 100.87, 99.66, 83.41, 83.33, 83.20, 83.16, 82.35, 81.53, 79.10, 78.91, 77.63, 77.12, 77.08, 77.04, 76.39, 76.25, 75.53, 75.24, 75.11, 74.85, 74.75, 74.68, 74.47, 74.41, 73.44, 72.88, 72.17, 72.12, 71.09, 71.05, 70.90, 70.03, 69.98, 68.59, 68.40, 66.01, 65.34, 64.42, 64.32, 62.92, 62.67, 62.51, 57.63, 57.47, 57.34, 57.31, 54.64, 54.5742.76, 25.12, 24.96, 24.73.

**Compound 6b.** Selected data of^1^H-NMR (D_2_O) *δ* 7.92 (d, *J* = 7.6 Hz, 2H), 7.70 (d, *J* = 7.2 Hz, 2H), 7.50 (t, *J* = 7.4 Hz, 2H), 7.44–7.41 (m, 2H), 5.13 (s, 1H), 4.99 (d, *J* = 9.5 Hz, 1H), 4.94 (s, 1H), 4.60 (d, *J* = 7.0 Hz, 2H), 4.44 (d, *J* = 7.6 Hz, 2H), 4.33 (m, 1H), 4.25 (s, 1H), 4.19 (s, 1H), 4.11 (s, 1H), 2.73–2.65 (m, 3H), 2.52 (dd, *J* = 15.0 Hz, 6.0 Hz, 1H), 2.06 (s, 9H), 2.02 (s, 6H), 1.71 (t, *J* = 12.2 Hz, 2H); ^13^C-NMR (D_2_O) *δ* 177.61, 177.44, 177.30, 176.24, 160.30, 146.58, 146.45, 143.60, 130.77, 130.26, 130.20, 127.92, 127.85, 122.88, 106.27, 103.99, 103.17, 102.86, 102.24, 102.01, 101.96, 99.63, 83.38, 83.32, 83.17, 81.32, 80.87, 79.07, 78.91, 78.82, 77.12, 77.05, 76.39, 76.23, 75.53, 75.41, 75.23, 75.11, 74.85, 74.75, 74.68, 74.41, 73.43, 72.89, 72.18, 72.12, 71.09, 70.91, 70.03, 69.97, 69.10, 68.39, 66.02, 65.34, 64.40, 64.32, 62.92, 62.63, 62.40, 57.58, 57.32, 56.4454.57, 49.63, 42.76, 41.29, 25.11, 24.94, 24.74, 24.65.

**Compound 7b.** Selected data of^1^H-NMR (D_2_O) *δ* 7.75 (d, *J* = 8.5 Hz, 1H), 7.06 (d, *J* = 8.5 Hz, 1H), 7.06 (s, 1H), 6.28 (s, 1H), 5.28 (d, *J* = 8.4 Hz, 1H), 5.13 (s, 1H), 4.95 (s, 1H), 4.64 (d, *J* = 7.8 Hz, 1H), 4.60 (d, *J* = 7.2 Hz, 2H), 4.44 (d, *J* = 5.4 Hz, 2H), 4.26 (s, 1H), 2.19 (s, 1H), 4.12 (s, 1H), 4.06 (t, *J* = 9.6 Hz, 1H), 2.66 (m, 2H), 2.45 (s, 3H), 2.10 (s, 3H), 2.07 (s, 3H), 2.03 (s, 3H), 1.72 (t, *J* = 12.2 Hz, 2H); ^13^C-NMR (D_2_O) *δ* 177.60, 177.44, 176.24, 167.35, 162.11, 156.68, 129.47, 118.29, 116.55, 114.14, 106.45, 106.26, 104.11, 103.20, 102.87, 102.85, 102.02, 101.97, 101.42, 99.69, 83.41, 83.33, 83.19, 82.33, 81.57, 79.09, 78.93, 77.54, 77.12, 77.08, 76.29, 76.25, 75.53, 75.22, 75.11, 74.87, 74.71, 74.41, 73.44, 72.90, 72.17, 72.12, 71.09, 71.05, 70.91, 70.02, 69.98, 68.38, 66.02, 65.34, 64.40, 64.32, 62.92, 62.54, 62.54, 57.61, 57.45, 57.33, 54.57, 42.77, 25.11, 24.96, 24.78, 24.73, 20.66.

**Compound 8b.** Selected data of^1^H-NMR (D_2_O) *δ* 8.27 (d, *J* = 9.0 Hz, 2H), 7.24 (d, *J* = 9.0 Hz, 2H), 5.28 (d, *J* = 164.26 Hz, 7.6 Hz, 1H), 5.13 (s, 1H), 4.95 (s, 1H), 4.16–4.60 (m, 3H), 4.44 (d, *J* = 6.3 Hz, 2H), 4.26 (s, 1H), 4.20 (s, 1H), 4.12 (s, 1H), 2.67 (m, 2H), 2.12 (s), 2.10 (s), 2.07 (s), 2.06 (s); ^13^C-NMR (D_2_O) *δ* 177.60, 177.44, 177.36, 176.24, 164.33, 145.35, 128.81, 119.12, 106.24, 104.09, 103.17, 102.86, 102.83, 102.26, 102.00 (d), 101.38, 101.10, 99.65, 83.40, 83.34, 83.26, 83.19, 82.37, 81.38 (t), 70.09, 78.90, 77.52 (t), 76.70 (t), 76.24, 75.53, 75.00, 74.41, 73.42, 72.90, 72.17, 72.12, 71.09, 71.06, 70.91, 68.56, 68.38, 66.01, 65.34, 64.42, 64.32, 62.92, 62.48 (d), 57.65, 57.32, 54.57, 42.76, 25.12, 24.95, 24.74.

**Compound 9b.** Selected data of^1^H-NMR (D_2_O) *δ* 8.26 (d, *J* = 9.0 Hz, 2H), 7.27 (d, *J* = 9.0 Hz, 2H), 5.76 (s, 1H), 5.13 (s, 1H), 4.94 (s, 1H), 4.60 (m, 4H), 4.44 (d, *J* = 7.8 Hz, 2H), 4.25 (d, *J* = 10.2 Hz, 2H), 4.19–4.18 (m, 2H), 4.11 (s, 1H), 2.66 (dd, *J* = 12.4 Hz, 4.4H, 2H), 2.07 (s, 3H), 2.06 (s, 3H), 2.02 (s), 1.71 (dt, *J* = 12.2 Hz, 4.5 Hz, 2H); ^13^C-NMR (D_2_O) *δ* 177.61, 177.43, 177.29, 176.24, 163.48, 145.00, 128.69, 119.32, 106.26, 104.09, 103.17, 102.86, 102.84, 102.27, 102.02, 101.94, 100.02, 99.65, 83.40, 83.31, 83.20, 82.42, 79.49, 79.09, 78.90, 77.10, 77.05, 76.38, 76.24, 75.52, 75.23, 75.11, 74.85, 74.73, 74.68, 74.41, 73.43, 72.90, 72.16, 72.12, 71.87, 71.70, 71.09, 71.05, 70.90, 70.02, 69.98, 68.55, 68.36, 66.03, 65.34, 64.41, 64.31, 62.93, 62.75, 62.71, 57.61, 57.32, 54.57, 42.76, 25.12,25.10, 24.90, 24.74.

**Compound 15b.** Selected data of^1^H-NMR (D_2_O) *δ* 7.09 (dd, *J* = 8.2 Hz, 6.6 Hz, 2H), 6.81 (dd, *J* = 8.2 Hz, 6.6 Hz, 2H), 6.80 (dd, *J* = 8.4 Hz, 7.8 Hz, 2H), 5.13 (s, 1H), 5.03 (d, *J* = 12.0 Hz, 1H), 4.94 (s, 1H), 4.68 (t, *J* = 7.2 Hz, 1H), 4.60 (m, 3H), 4.55 (t, *J* = 6.0 Hz, 1H), 4.50 (t, *J* = 7.8 Hz, 1H), 4.44 (d, *J* = 7.2 Hz, 1H), 4.37 (t, *J* = 5.4 Hz, 1H), 4.28–4.19 (m, 9H), 4.11 (s, 1H), 2.28–2.18 (m, 7H), 2.06 (s), 2.05 (s), 2.04 (s), 2.02 (s), 2.00 (s), 1.80 (m, 1H), 1.72 (t, *J* = 12.0 Hz, 2H), 1.62 (m, 1H), 1.52 (m, 1H), 1.14 (d, *J* = 6.0 Hz, 3H); ^13^C-NMR (D_2_O) *δ* 184.06, 183.99, 180.35, 178.28, 177.61, 177.43, 177.39, 177.25, 177.12, 176.39, 176.24, 175.73, 175.60, 175.42, 175.20, 175.08, 174.95, 174.34, 159.32, 157.29, 157.15, 133.26, 133.14, 130.42, 130.37, 118.28, 118.16, 106.26, 103.99, 103.17, 102.86, 102.25, 102.01, 101.95, 99.64, 83.39, 83.30, 83.17, 82.33, 81.24, 80.91, 79.07, 78.91, 78.85, 77.12, 77.04, 76.39, 76.24, 75.52, 75.45, 75.23, 75.11, 74.84, 74.75, 74.68, 74.41, 73.44, 72.89, 72.16, 72.12, 71.09, 70.90, 70.03, 69.98, 69.37, 68.58, 68.39, 66.02, 65.34, 64.40, 64.32, 63.49, 62.93, 62.91, 62.63, 62.53, 62.39, 59.07, 58.38, 57.91, 57.57, 57.34, 57.31, 56.81, 56.72, 56.46, 55.67, 55.38, 54.57, 52.63, 43.08, 42.75, 39.03, 38.70, 38.59, 36.33, 36.21, 33.58, 30.69, 30.36, 29.90, 29.26, 26.94, 25.11, 24.93, 24.80, 24.74, 24.42, 21.53.

**Preparation of compound glycopeptide 15a.** After the Rink amide resin (0.53 mmol g^−1^, 150 mg) was swollen for 1 h in dimethylformamide (DMF), Fmoc deprotection was carried out by treatment with 20% (v/v) piperidine/DMF (5 min × 1 and 10 min × 1), followed by washing with DMF (× 3). Fmoc-Arg(pbf)-OH (155 mg, 0.239 mmol) in DMF in the presence of HATU (91 mg, 0.24 mmol) and *N*,*N*-diisopropylethylamine (42 µl, 0.24 mmol) was introduced to the resin at room temperature for 90 min, followed by washing with DMF (×3). The unreacted amine on the resin was capped with Ac_2_O : pyridine = 3 : 2 (v/v) at room temperature for 30 min and the resin was washed with DMF (×3). The subsequent peptide chain was assembled by deprotection and coupling. Fmoc deprotection was carried out by treatment with 20% (v/v) piperidine/DMF (5 min × 1 and 10 min × 1) and the resin washed with DMF (×3). The sequential coupling of activated Fmoc-amino acid (3.0 eq.) in DMF in the presence of HATU (91 mg, 0.24 mmol) and *N*, *N*-diisopropylethylamine (42 µl, 0.24 mmol) was carried out at room temperature for 90 min, followed by washing with DMF (×3). The deprotection and coupling cycles were repeated until the full peptide sequence was completed. After completion, the peptide–resin was washed with MeOH (×3) and dried for 2 h *in vacuo*. The peptide was cleaved from the resin with TFA in the presence of triisopropylsilane and distilled water (95 : 2.5 : 2.5) for 60 min at room temperature, concentrated by evaporation after filtration and precipitated with Et_2_O at 0°C. The resulting precipitate was collected by filtration, washed with Et_2_O and dried *in vacuo* to afford the crude peptide. Preparative HPLC was performed using a C18 reverse phase column (Mightysil RP-18 GP, Kanto Chemical Co., Inc., Tokyo) with 2% MeCN for 2 min followed by a linear gradient of 15–45% MeCN over 30 min in 0.1% aqueous TFA at room temperature at a flow rate of 8 ml min^−1^, detected at 214 nm. The fraction was immediately frozen using liquid N_2_ and lyophilized to afford the desired peptide (53 mg, 40% yield from the resin loading). MS (MALDI-TOF MS): *m/z* calcd for C_66_H_95_N_16_O_28_ [M+H]^+^ 1559.7, found 1560.4. Retention time: 17.5 min.

To a solution of the peptide (53 mg, 0.032 mmol) in H_2_O (1950 µl), hydrazine monohydrate (53 µl, 1.1 mmol) was added (final conc. = 0.016 M). The mixture was stirred at room temperature for 3 h and then directly purified using a C18 reverse phase column (Mightysil RP-18 GP, Kanto Chemical Co., Inc., Tokyo) with 2% MeCN for 2 min, followed by a linear gradient of 12.5–35% MeCN over 45 min in 0.1% aqueous TFA at room temperature at a flow rate of 8 ml min^−1^, detected at 214 nm. The fraction was immediately frozen using liquid N_2_ and lyophilized. The peptide was additionally purified using a gel filtration column (Sephadex™ LH-20; GE Healthcare Japan, Tokyo) with water as eluent and the peak fraction was immediately frozen using liquid N_2_ and lyophilized to afford the desired peptide (33 mg, 67% yield).

MS (MALDI-TOF MS): *m/z* calcd for C_60_H_89_N_16_O_25_ [M+H]^+^ 1433.6, found 1433.7. Retention time: 13.9 min.

### 2.3. Preparation of antibody with homogeneous glycan

#### 2.3.1. Deglycosylation of antibody by EndoS: preparation of **17**

Twenty milligrams of anti-CCR4 antibody (4 mg ml^−1^ in 50 mM sodium phosphate buffer, pH 7.4) was incubated with 30 µg of EndoS for 20 h. The deglycosylation was monitored by an UPLC system equipped with an HILIC column (ACQUITY UPLC Glycoprotein BEH Amide Column, 300 Å, 1.7 µm, 2.1 mm × 150 mm, Waters). The antibody peaks were detected using intrinsic fluorescence of tryptophan residues (excitation wavelength, 280 nm; fluorescence wavelength, 320 nm). Antibody was eluted using a gradient of mobile phases A and B (A: 0.1% TFA/0.3% hexafluoro-2-propanol/H_2_O; B: 0.1% TFA/0.3% hexafluoro-2-propanol/acetonitrile). After the reaction was completed, the antibody was purified from the reaction mixture using Protein A Sepharose CL-4B (GE Healthcare Japan, Tokyo).

#### 2.3.2. Transglycosylation of deglycosylated antibody with endo-CC N180H: preparation of **18**

To the deglycosylated antibody solution (6.5 mg ml^−1^, 24 µl), 45 µl of endo-CC N180H solution (0.86 mU μl^–1^ in 20 mM Tris–HCl, pH 7.5) and SGP **1** were added and incubated at 30°C for 48 h. The final concentrations of antibody and enzyme were 1.4 mg ml^−1^ and 0.34 U ml^−1^, respectively. The final concentration of SGP **1** was 395 mg ml^−1^, which is nearly saturated concentration. After the reaction, fully glycosylated antibody was isolated using a cation-exchange column (Mono S 5/50 GL, GE Healthcare Japan, Tokyo) using a gradient of mobile phases A and B (A: 50 mM sodium acetate, pH 4.3; B: 50 mM sodium acetate, 1 M NaCl, pH 4.3). The antibody peaks were detected using the absorbance at 280 nm. The purified product was checked by ESI-MS. Yield was calculated from the ratio of peak areas of UPLC spectrum. For reaction profile, see the electronic supplementary material.

#### 2.3.3. Electrospray ionization mass spectrometric analysis of compound **18**

ESI-MS analysis of the antibody product was performed using a QSTAR ELITE quadrupole-time-of-flight mass spectrometer (AB Sciex) equipped with a Nanospray Tip (Humanix, Hiroshima, Japan). The antibody dissolved in 50** **mM sodium phosphate buffer (pH 7.4) was treated with 10** **mM dithiothreitol for 15** **min at 37°C and then the sample was desalted using a self-made C8 (3 M Empore high-performance extraction discs) Stage Tip. Protein was eluted with 70% (v/v) acetonitrile/0.1% (v/v) formic acid to a concentration of 33 pmol µl^−1^ and directly transferred to the mass spectrometer with an applied voltage of 1.35** **kV. Mass spectra were deconvoluted using Analyst QS software (AB Sciex).

## 3. Results and discussion

**Scheme 2.**
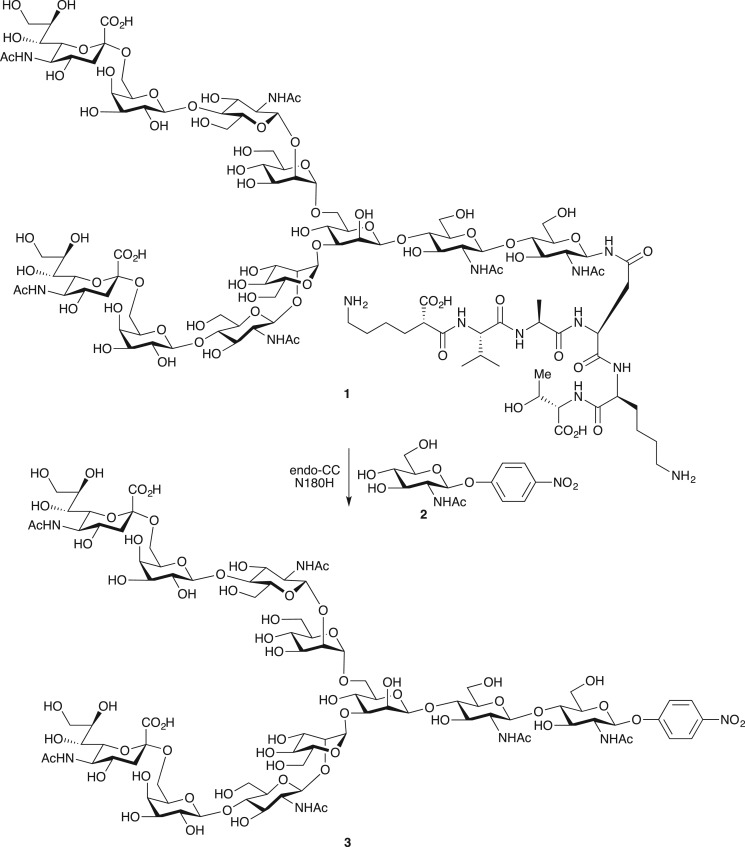
Glycan transfer from SGP **1** to *p*NP-GlcNAc **2**.

**Figure 1.**
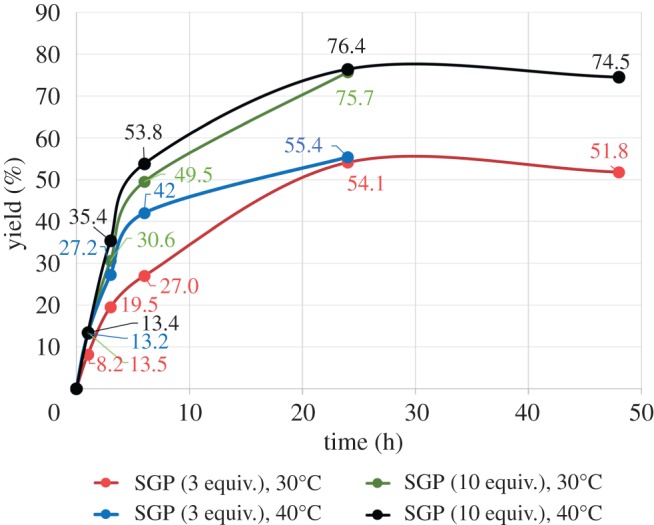
Time-course of transglycosylation to *p*NP-GlcNAc **2** depends on the amount of SGP and the reaction temperature.

The synthetic activity of endo-CC N180H has been reported using oxazoline or full-length SGP **1** as a donor [26,27]. Here reaction conditions of endo-CC N180H were optimized using SGP **1** as donor and *p*-nitrophenyl (*p*NP)-GlcNAc **2** as acceptor (scheme 2). The transfer reaction was monitored by HPLC at 280** **nm, and yields were calculated based on peak ratios. When three equivalents of SGP were used at 30°C, there was a gradual increase in product to 54% yield after 24** **h (red line in figure 1), and 52% after 48** **h. Because it has been reported that endo-CC is stable at temperatures of up to 50°C for 10** **min, the reaction temperature was raised to 40°C. The initial reaction rate was accelerated at 40°C (blue line in figure 1), but the yield after 24** **h was similar to the yield at 30°C (55%). We infer that endo-CC N180H gradually decomposed at 40°C over time. When 10 equivalents of SGP were used, and the enzyme equilibrium shifted in the product direction, yield increased to 76% at 30°C (green line in figure 1). Again, reaction temperature did not affect yield after 24** **h (green and black lines in figure 1). Endo-M-N175Q-mediated reactions at 30°C and 40°C gave the product in 76% and 62% yields, respectively. Because endo-M-N175Q was deactivated at 40°C, yield of **3** did not change at 40°C after 1** **h. These results show that endo-CC N180H was thermally stable compared to endo-M-N175Q as reported [29].

**Figure 2.**
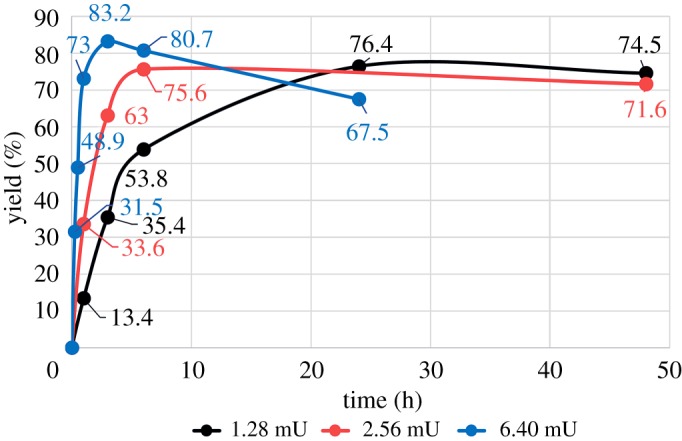
Time-course of transglycosylation to *p*NP-GlcNAc **2** depends on the concentration of endo-CC N180H.

Changes in pH (5.0, 6.0, and 7.5) had little effect on reaction rate or yield after 24** **h (electronic supplementary material). An increase in the concentration of endo-CC N180H to 6.4 mU (blue line in figure 2) gave an optimum yield of 83% at 6** **h. The yield gradually decreased with time owing to hydrolysis of the product. Normally, ENGase mutants do not accept full-length SGP as a donor in transglycosylation reactions because the mutation usually interferes with the hydrolysis activity of the enzyme. Since endo-CC N180H can use SGP as a donor substrate for glycan transfer, we conclude that the endo-CC mutant retains the ability to hydrolyse full-length SGP necessary for glycan transfer. Similar hydrolysis activity was also found in the endo-M mutant N175Q, which can also use SGP as a donor substrate [20]. Indeed, truncated product from SGP **1** was observed.

**Figure 3.**
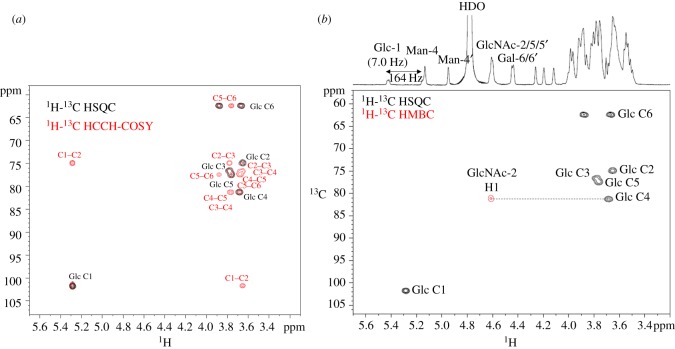
(*a*) 2D ^1^H-^13^C HSQC (black) and HCCH-COSY (red) spectra and (*b*) 2D ^1^H-^13^C HSQC (black) and HMBC (red) spectra of compound **9b**.

Once the reaction parameters had been optimized, a range of glycosyl acceptors was investigated. Acceptor tolerance of wild-type endo-M is rather broad, with oligosaccharide transfers to *p*NP-mannose, *p*NP-glucose and 1,3-diol containing structures, although the products were not rigorously defined [31,32]. Product yields were low because of rapid hydrolysis by endo-M. We expected that endo-CC N180H may also react with various acceptors to form glycoconjugates that may be useful biological tools. For example, a ^13^C-labelled acetyl group could facilitate NMR analyses, an azide carrying neo-glycan could be used for conjugation, and a fluorophore-containing glycan could be advantageous in enzyme assays. We prepared several acceptors for these purposes, and substrate tolerance was compared to endo-M-N175Q (table 1). The ^13^C-labelled GlcNAc derivative **4a**, the azide carrying a GlcNAc derivative **5a**, the Asn-linked GlcNAc **6a** and the 4-methylumbelliferyl group containing compound **7a** were good acceptors, as good as *p*NP-GlcNAc. The product formed by acceptor **7b** is useful for assaying hexosaminidases such as peptide-*N*-glycosidase F (PNGase F), because a fluorescent signal appears only after the hydrolysis of the glycan [33]. *p*NP-glucose **8a** was also a substrate for endo-CC N180H. In order to prove that glycan was transferred to position 4 of the glucose molecule, we used *p*NP-[U-^13^C]-glucose as an acceptor. The product was analysed by a series of NMR measurements enriched with ^13^C. Assigning NMR signals from the ^13^C-labelled glucose residue (C1–C6) was attained by 2D ^1^H-^13^C HSQC spectroscopy and HCCH-COSY experiments (figure 3*a*). The 2D ^1^H-^13^C HMBC spectrum of the product showed a correlation peak between GlcNAc-2 H1 and Glc-1 C4 (figure 3*b*), indicating that glycan was indeed transferred to position 4 of the glucose residue. The one-bond C─H coupling constant (^1^*J*_CH_) was obtained from ^13^C-coupled 2D ^1^H-^13^C HSQC spectrum (electronic supplementary material). The ^1^*J*_CH_ of GlcNAc-2 H1─C1 was found to be 168** **Hz, suggesting that GlcNAc-2 is **β**-linked. *p*NP-mannose **9a** was a poorer acceptor and 41% conversion to product occurred after 24** **h. In **9b**, a correlation was observed between GlcNAc-2 C1 and Man-1 H4 in the 2D ^1^H-^13^C HMBC spectrum, showing that the glycan was transferred to position 4 of the mannose residue (electronic supplementary material). The linkage was found to be *β*, as judged by ^1^*J*_CH_ and ^3^*J*_H1,H2_ of GlcNAc-2 anomeric signal. Unfortunately, *p*NP-galactose **10** and *p*NP-xylose **11** were not substrates. The substrate tolerance of endo-CC N180H was similar to that of endo-M-N175Q, but yields by endo-CC N180H were slightly higher than by endo-M-N175Q, except compound **6**. As reported for the endo-M-catalysed reaction, the 1,3-diol structure and equatorial hydroxy group at C4 are important for enzyme recognition. *p*NP-sialic acid **12**, disaccharide **13** [34] and tetrasaccharide **14** [35] were not substrates, although they possess the 1,3-diol structure. Glycopeptide **15a**, a trypsin digestion fragment of an antibody containing an *N*-glycan attachment at Asn297, showed an 84% yield under the above conditions.

**Table 1.** Scope and limitation of acceptors in glycan transfer reaction mediated by endo-CC N180H and endo-M-N175Q. 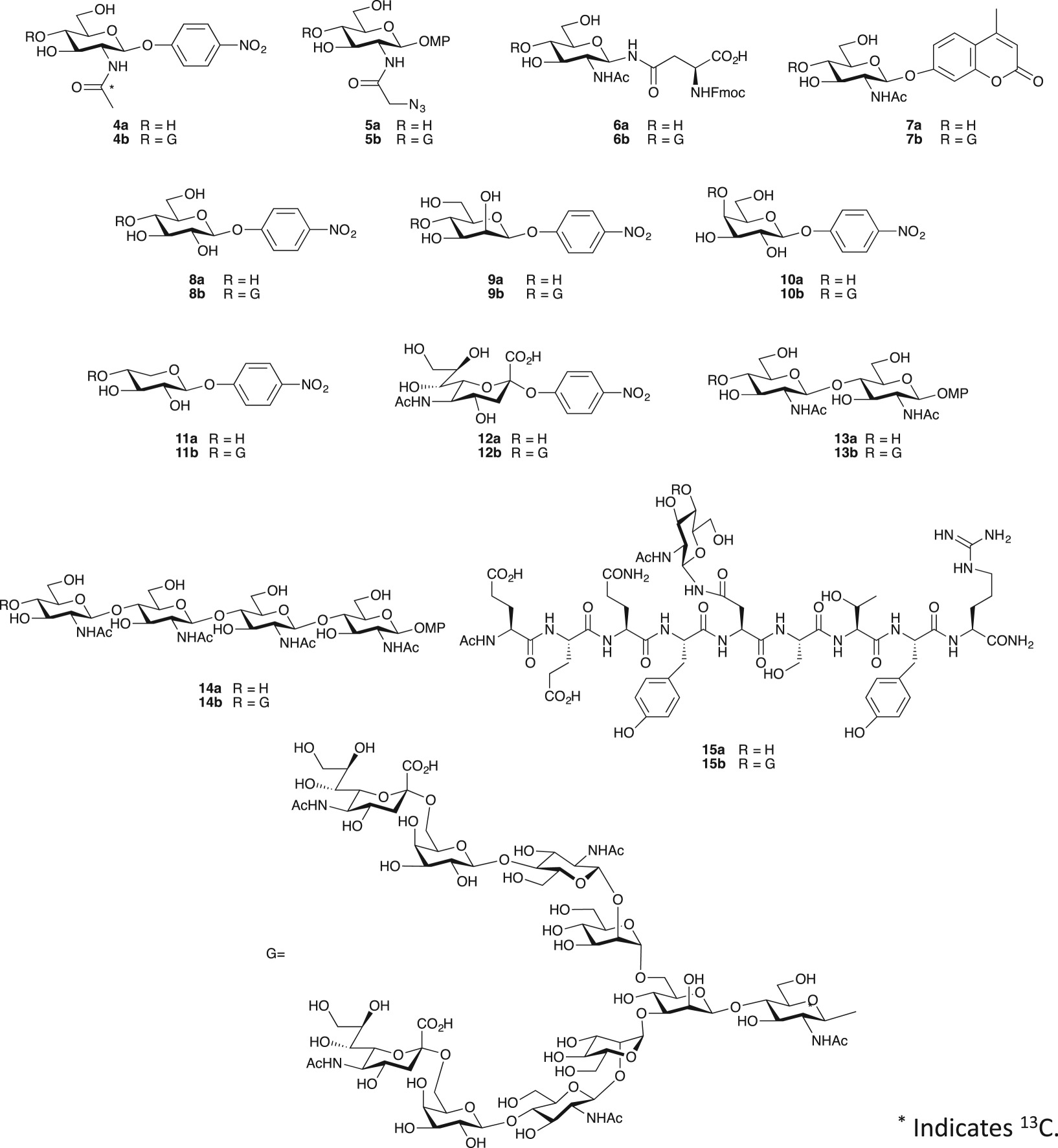

entry	substrate	product	yield (%) by endo-CC N180H^a^	yield (%) by endo-M-N175Q^b^
1	**4a**	**4b**	82	79
2	**5a**	**5b**	80	74
3	**6a**	**6b**	79	81
4	**7a**	**7b**	80	76
5	**8a**	**8b**	79	75
6	**9a**	**9b**	41	25
7	**10a**	**10b**	0	0
8	**11a**	**11b**	0	0
9	**12a**	**12b**	0	0
10	**13a**	**13b**	0	0
11	**14a**	**14b**	0	0
12	**15a**	**15b**	84	73

^a^Endo-CC N180H (6.4 mU), acceptor (3 mM), SGP (30 mM), 20 mM Tris–HCl (pH 7.4) containing 10% DMSO, 40°C.

^b^Endo-M-N175Q (6.4 mU), acceptor (3 mM), SGP (30 mM), 20 mM Tris–HCl (pH 7.4) containing 10% DMSO, 30°C.

**Scheme 3.**
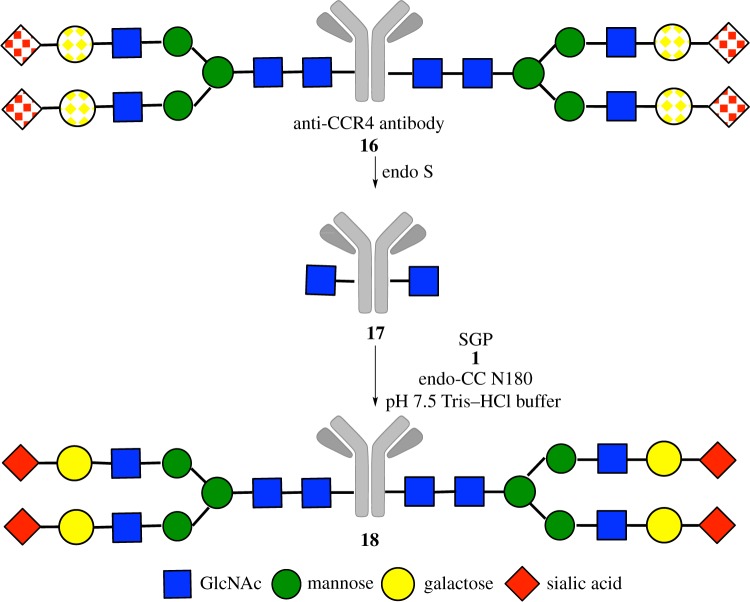
Glycan remodelling of anti-CCR4 antibody using SGP **1** as a donor. Dot-circles and squares indicate a heterogeneous portion.

**Figure 4.**
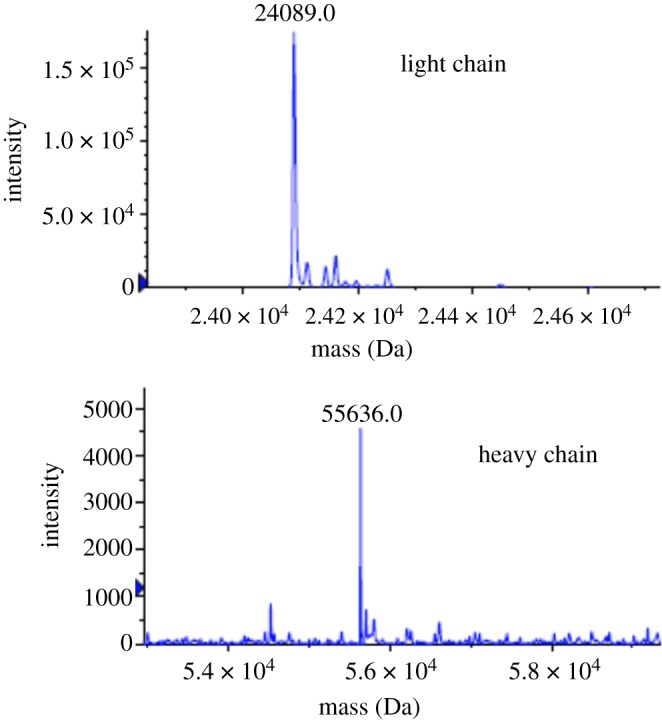
Deconvoluted ESI-MS spectra of reduced antibody product **18**.

Finally, we attempted glycan transfer to a therapeutic antibody again using SGP **1** as donor (scheme 3). The previous use of oxazoline can lead to side reactions, through a reaction with the amino group of lysine residues (scheme 1) [23–25]. We thought that SGP may be an alternative donor because it lacks a highly reactive group. We chose endoS-treated, core-fucose-deficient anti-CCR4 antibody as substrate, because it contains 4- and 6-diol structure in the Asn-linked GlcNAc residue. Heterogeneous *N*-glycan was removed in advance by endoS and glycan transfer initiated in a mix of SGP and endo-CC N180H under slightly basic conditions (pH 7.5). High concentrations of SGP (molar ratio of SGP to antibody = 15 000) increased yield of the fully glycosylated antibody to 85% yield (UPLC calculation yield). The fully glycosylated antibody was isolated from partially glycosylated and GlcNAc-type antibodies using cation-exchange column chromatography. Homogeneity of the purified product **18** was confirmed by mass spectrometry analysis after dithiothreitol reduction (figure 4). Observed mass spectral peaks originating from light chain (24 089** **Da) and heavy chain (55 636** **Da) showed homogeneity of the glycan and the absence of side reactions.

## 4. Conclusion

In this paper, we report on the scope and limitations of acceptors from monosaccharides to an antibody in the endo-CC N180H glycan transfer reaction. Endo-CC N180H had similar substrate acceptance and gave slightly higher yields compared with endo-M-N175Q, a widely used ENGase mutant. The 1,3-diol structure is important in acceptor molecules, but not necessarily the sole requirement. Several low-molecular-weight acceptors, including some with useful labels or functional moieties, were synthesized by the glycosyl transfer reaction. Glycan transfer from SGP to an antibody with reduced side reactions is demonstrated, although a large amount of SGP was required. Homogeneous *N*-glycan attachments for monosaccharides, peptides and proteins incorporating ^13^C, an azide group for conjugation, and a fluorescent moiety are possible candidates for potential use in many biological applications.

Furthermore, glycan remodelling of a therapeutic antibody was achieved without side reactions, and the homogeneity was proved by mass spectrometry without glycan cleavage.

## Supplementary Material

Acceptor range of endo-β-N-acetylglucosaminidase Mutant endo-CC N180H: from Monosaccharide to Antibody: Supporting information

## Data accessibility

Electronic supplementary material including acceptor preparation, ^1^H- and ^13^C-NMR spectra, and time-course of glycan transfer reaction by endo-CC N180H is available.
