# Supplementary material for: Acceptor range of endo-β-N-acetylglucosaminidase mutant endo-CC N180H: from monosaccharide to antibody
Source: R Soc Open Sci. 2018 May 16;5(5):171521. doi: 10.1098/rsos.171521 (PMC5990847; doi:10.1098/rsos.171521)

Supporting Information

Acceptor range of endo-β-*N*-acetylglucosaminidase Mutant endo-CC N180H: from Monosaccharide to Antibody

Shino Manabe*[a], Yoshiki Yamaguchi*[b], Junpei Abe[a], Kana Matsumoto[b], and Yukishige Ito[a]

[a] RIKEN, Synthetic Cellular Chemistry Laboratory
[b] RIKEN, Structural Glycobiology Team

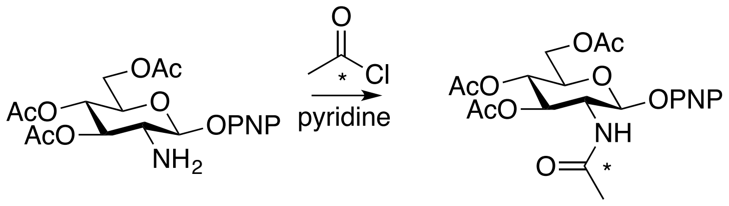

To a solution of amine [1] (100 mg, 0.185 mmol) in pyridine (1 mL), acetyl-2-^{13}C chloride (0.1 mL) was dropped at -40 °C. The mixture was warmed to room temperature, and stirred at room temperature overnight, then mixture was evaporated. The residue was dissolved in $CHCl_3$ and washed with 1 M HCl. The aqueous layer was extracted with $CHCl_3$, and the combined layers were washed with brine. After drying the extract over Na_2SO_4, the mixture was concentrated. The mixture was purified by silica gel column chromatography ($CHCl_3$:MeOH 9:1) to give ^{13}C-acetamide (60.0 mg, 71%).

^{1}H-NMR (CD_3OD) δ 8.23 (d, *J* = 7.2 Hz, 2H), 7.20 (d, *J* = 7.2 Hz, 2H), 5.48 (d, *J* = 8.4 Hz, 1H), 5.36 (t, *J* = 10.0 Hz, 1H), 5.08 (t, *J* = 10.0 Hz, 1H), 4.31 (dd, *J* = 4.8, 12.0 Hz, 1H), 4.17-4.08 (m, 3H), 2.04 (s, 3H), 2.03 (s, 3H), 2.01 (s, 3H), 1.90 (d, *J* = 6.0 Hz, 3H); ^{13}C-NMR (CD_3OD) δ 173.71, 126.72, 117.70, 98.89, 73.63, 73.32, 69.83, 63.05, 55.31, 24.51, 20.60, 20.56, 20.52; [α] -47 (*c* 1.0, $CHCl_3$).

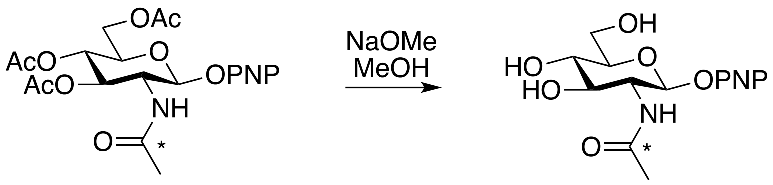

To a solution of acetate in MeOH, 28% solution of NaOMe (MeOH) was added. After overnight, reaction mixture was neutralized with Amberlyst 15E. After filtration, the resin was washed with MeOH. After concentration, the residue was purified by HPLC (Mightysil RP-18 GP with 2% MeCN for 2 min, followed by a linear gradient of 20-50% MeCN over 30 min in 0.1% aqueous TFA at room temperature at a flow rate of 8 mL/min, detected at 214 and 280 nm).

^{1}H-NMR (CD_3CD) δ 8.20 (d, *J* = 9.2 Hz, 2H), 7.17 (d, *J* = 9.2 Hz, 2H), 5.20 (d *J* = 8.0 Hz, 1H), 3.94-9.90 (m, 2H), 3.70 (dd, *J* = 12.4, 6.4 Hz, 1H), 3.59 (t, *J* = 8.8 Hz, 1H), 3.49 (m, 1H), 3.45-3.40 (m, 1H), 3.30 (m, 2H), 1.97 (d, *J* = 6.4 Hz, 3H); ^{13}C-NMR δ 176.79, 173.93, 163.72, 144.00, 126.66, 117.68, 100.02, 78.53, 75.67, 71.72, 62.45, 57.13, 23.22.92; HRMS calcd for $[C_{13}{}^{13}CH_{18}N_2O_8+Na]^+$ 366.0994, found 366.0995.

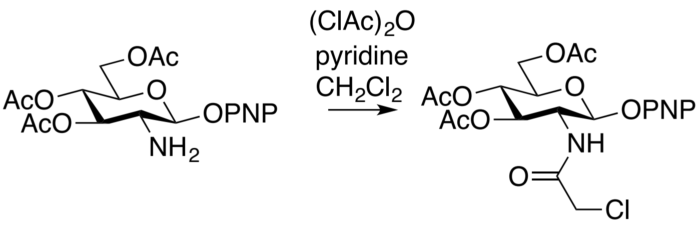

To a solution of amine (77.4 mg, 0.147 mmol) in pyridine (58 μL) and CH_2Cl_2 (2 mL), $(ClAc)_2O$ (50.0 mg, 0.294 mmol) was added at 4 °C. The mixture was stirred at room temperature overnight. The mixture was diluted with $CHCl_3$ and washed with 1 M HCl. After extraction with $CHCl_3$ several times, the combined layers were washed with brine and dried over Na_2SO_4. After concentration, the residue was purified by silica gel column chromatography ($CHCl_3$:MeOH 9:1) to give chroloacetyl compound (66.0 mg, 88 %).

^{1}H-NMR ($CDCl_3$) δ 8.21 (d, J = 9.2 Hz, 2H), 7.08 (d, J = 9.2 Hz, 2H), 6.78 (d, J = 8.0 Hz, 1H), 5.58 (d, J = 8.0 Hz, 1H), 5.53 (t, J = 10.0 H, 1H), 5.16 (t, J = 9.2 Hz, 1H), 4.30 (dd, J = 12.4, 6.0 Hz, 1H), 4.19 (dd, J = 12.4, 2.4 Hz, 1H), 4.04 (d, J = 16.0 Hz, 1H), 4.00 (d, J = 16.0 Hz, 1H), 4.13-3.95 (m, 2H), 2.09 (s, 3H), 2.08 (s, 6H); ^{13}C-NMR ($CDCl_3$) δ 170.43, 169.33, 166.68, 161.17, 143.21, 125.78, 116.64, 97.38, 72.49, 70.66, 68.06, 55.04, 42.37, 20.68, 20.62, 20.59; $[\alpha]^{24}_D$ -20.8 (c = 1.0, $CHCl_3$); HRMS calcd for $[C_{20}H_{23}ClN_2O_{11}+Na]^+$ 525.0883, found 525.0885.

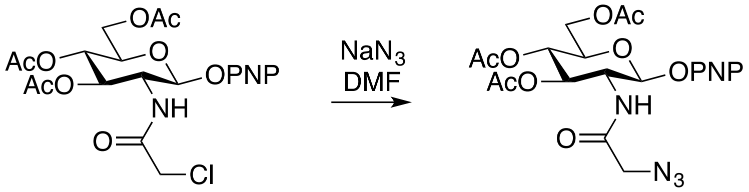

To a solution of chloride (61.2 mg, 0.122 mmol) in DMF (0.3 mL), NaN_3 (50 mg, 0.76 mmol) was added. The mixture was stirred at 50 °C overnight. Then, the mixture was cooled to room temperature, and diluted with $CHCl_3$. The organic layer was washed with sat. $NaHCO_3$ and brine. After drying over Na_2SO_4, the mixture was concentrated. The residue was purified by preparative TLC ($CHCl_3$:MeOH 95:5) to give 49.9 mg (80%) of compound.

^{1}H-NMR ($CDCl_3$) δ 8.20 (d, J = 9.2 Hz, 2H), 7.07 (d, J = 9.2 Hz, 2H), 6.58 (d, J = 8.4 Hz, 1H), 5.58 (d, J = 7.6 Hz, 1H), 5.52 (t, J = 10.4 Hz, 1H), 5.16 (t, J = 9.6 Hz, 1H), 4.30 (dd, J = 5.2 Hz, 12.0 Hz, 1H), 4.18 (dd, J = 2.4, 12.0 Hz, 1H), 4.11-3.91 (m, 4H), 2.08 (s, 9H);

^{13}C-NMR ($CDCl_3$) δ 170.56, 170.42, 169.34, 167.28, 143.17, 125.77, 116.57, 97.35, 72.45, 70.99, 68.09, 61.96, 54.92, 52.57, 20.68, 20.61; [α] -15.3 (c = 0.92, $CHCl_3$); HRMS calcd for $[C_{20}H_{35}N_5O_{11}+Na]^+$ 532.1292, found 532.1284.

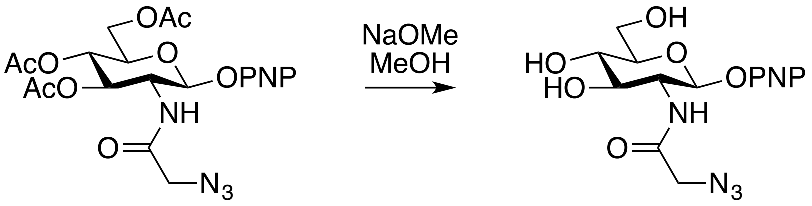

To a solution of acetate (46.2 mg, 0.0908 mmol) in MeOH (1 mL), 1 drop of 28% NaOMe solution (MeOH) was added. After overnight, the mixture was neutralized with Amberlyst 15 and filtered. After concentration, the residue was purified by HPLC (Mightysil with 2% MeCN for 2 min, followed by a linear gradient of 27.5-42.5% MeCN over 30 min in 0.1% aqueous TFA at room temperature at a flow rate of 8 mL/min, detected at 214 and 280 nm.).

^{1}H-NMR (CD_3OD) δ 8.20 (d, J = 9.2 Hz, 2H), 7.18 (d, J = 9.2 Hz, 2H), 5.26 (d, J = 8.4 Hz, 1H), 4.85-4.90 (m, 4H), 3.72 (dd, J = 11.6, 5.2 Hz, 1H), 3.64 (dd, J = 10.4, 8.8 Hz, 1H), 3.51 (m, 1H), 3.44 (t, J = 9.2 Hz, 1H); ^{13}C-NMR (CD_3OD) δ 163.65,144.06, 126.65, 117.74, 99.85, 78.59, 75.41, 71.74, 62.45, 57.20, 53.17; HRMS calcd for $[C_{14}H_{17}N_5O_8+Na]^+$ 406.0975, found 406.0970.

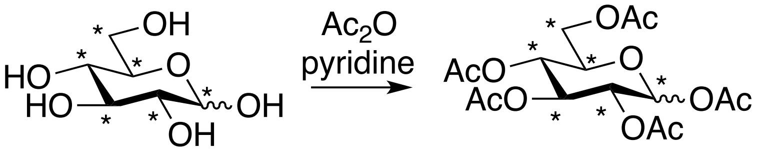

To a solution of D-[UL-^{13}C] glucose (300.0 mg, 1.61 mmol) in pyridine (20 mL), Ac_2O (20 mL) was added. The mixture was stirred at room temperature overnight. After concentration, the mixture was dissolved in $CHCl_3$ and washed with 1 M HCl. The aqueous layer was extracted with $CHCl_3$. The combined layers were washed with brine. After drying over Na_2SO_4 and concentration, the mixture was purified by silica gel column chromatography (hexane:EtOAc 7:3-1:1) to give pentaacetate as an α/β mixture (600 mg, 94%). HRMS calcd for $[C_{10}{}^{13}C_6H_{22}O_{11}+Na]^+$ 419.1261, found 419.1250.

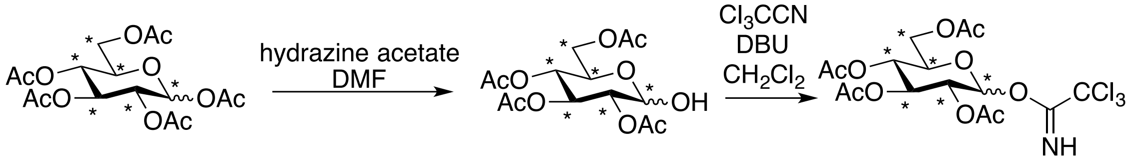

To a solution of acetate (0.72 g, 1.85 mmol) in DMF (2 mL), hydrazine acetate (336 mg, 3.65 mmol) was added. After overnight, the mixture was diluted with $CHCl_3$ and washed with water and brine. After the mixture was dried over Na_2SO_4, the mixture was concentrated. The residue was purified by silica gel column chromatography (hexane:EtOAc 7:3-1:1) to give hemiaetal as an α/β mixture (0.35 g, 54%). HRMS calcd for $[C_8{}^{13}C_6H_{20}O_{10}+Na]^+$ 377.1155, found 377.1153.

To a solution of hemiacetal (351 mg, 1.01 mmol) in Cl_3CCN (1 mL, 10 mmol) and CH_2Cl_2 (2 mL), DBU (50 μL, 0.033 mmol) was added at 4 °C. After 1h, the mixture was purified by silica gel column chromatography (hexane:EtOAc 7:3) to give imidate as an α/β mixture (450 mg, 91%).

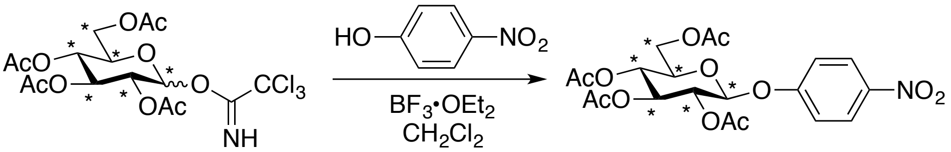

As reported procedure,[2] to a solution of imidate (450mg, 0.915 mmol) and *p*-nitrophenol (400 mg, 2.88 mmol) in CH_2Cl_2, BF_3•OEt_2 (13 μL, 0.091 mmol) was added at 4 °C. After 1.5 h, sat. $NaHCO_3$ was added, and the aqueous phase was extracted with $CHCl_3$. The combined layers were washed with brine, and dried over Na_2SO_4. After concentration, the residue was purified by silica gel column chromatography (hexane:EtOAc 7:3) to give *p*-nitrophenyl glucoside (386 mg, 90%).

^{1}H-NMR ($CDCl_3$) δ 8.15 (dd, *J* =7.6 Hz, 2.8 Hz, 2H), 7.01 (dd, *J* = 7.6 Hz, 2.8 Hz, 2H), 5.43-5.31 (m, 2H), 5.06 (m, 1H), 4.94 (m, 1H), 4.38 (m, 0.5 H), 4.28 (m, 0.5 H), 4.04 (m, 1H), 3.94 (m, 0.5 H), 3.68 (m, 0.5 H), 2.01 (s, 3H), 2.00 (s, 3H), 200 (s, 3H), 1.98 (s, 3H); ^{13}C-NMR ($CDCl_3$) δ 170.43, 170.15, 169.34, 169.18, 143.28, 125.80, 116.60, 98.09(d), 72.63(t), 70.86(t), 67.95(t), 61.79(d), 20.66, 20.57; HRMScalcd for $[C_{14}{}^{13}C_6H_{23}NO_{12}+Na]^+$ 498.1319, found 498.1317.

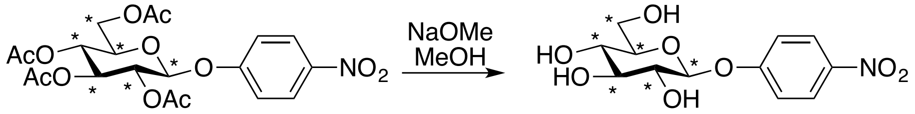

To a solution of acetate (30 mg, 0.0640 mmol) in MeOH (0.5 mL) and THF (0.5 mL), 1 drop of 28% NaOMe solution (MeOH) was added. After overnight, acetic acid was added to neutralize the mixture. After concentration, *p*-nitrophenyl D-[UL-$^{13}C_6$] glucoside was purified by HPLC (Mightysil RP-18 GP with 2% MeCN for 2 min, followed by a linear gradient of 20-50% MeCN over 30 min in 0.1% aqueous TFA at room temperature at a flow rate of 8 mL/min, detected at 214 and 280 nm).

^{1}H-NMR (CD_3OD) δ 8.21 (d, *J* = 7.2 Hz, 4H), 7.24 (d, *J* = 7.2 Hz, 4H), 5.25 (d, *J* = 7.2 Hz, 1H), 4.07 (d, *J* = 11.6 Hz, 1H), 3.88 (m, 1H), 3.70-3.53 (m 5H), 3.28 (m, 1H); ^{13}C-NMR (CD_3OD) δ 126.6, 117.8, 101.96(d), 78.83(t), 78.00(t), 74.70(t), 71.39,(t) 62.40(d), HRMS calcd for $[^{13}C_{12}H_{15}NO_8+Na]^+$ 330.0897, found 330.0891.

MS and HPLC spectra of glycopeptide 15a.

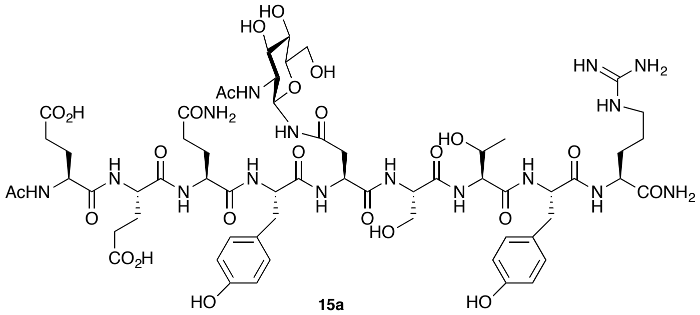

15a

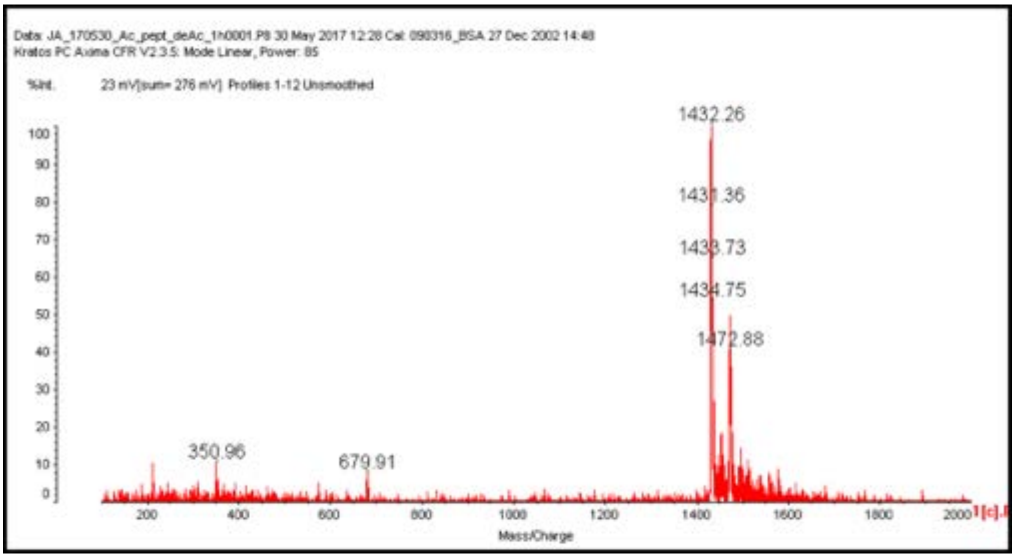

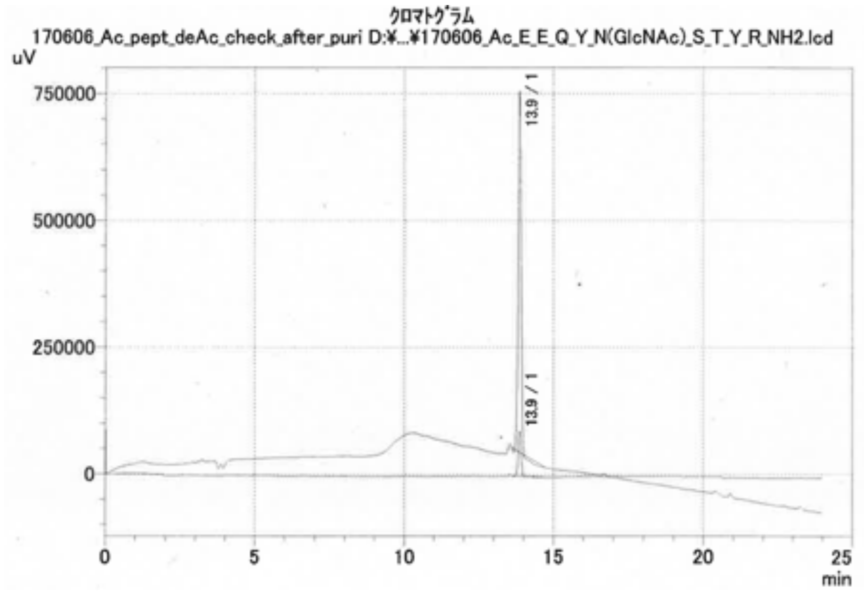

[1] K. J. Loft, P. Bojarová, K. Slámová, V. Křen, S. J. Williams, *ChemBioChem*, **2009**, 10, 565-576.

[2] Y. Li, H. Mo, G. Lian, B. Yu, *Carbohydr. Res*. **2012**, *363*, 14-22.

2D ^{13}C-coupled ^{1}H-^{13}C HSQC spectrum of glycan-transferred *p*NP-[U-^{13}C] glucose. $^{1}J_{CH}$ values are labeled for each anomeric signal.

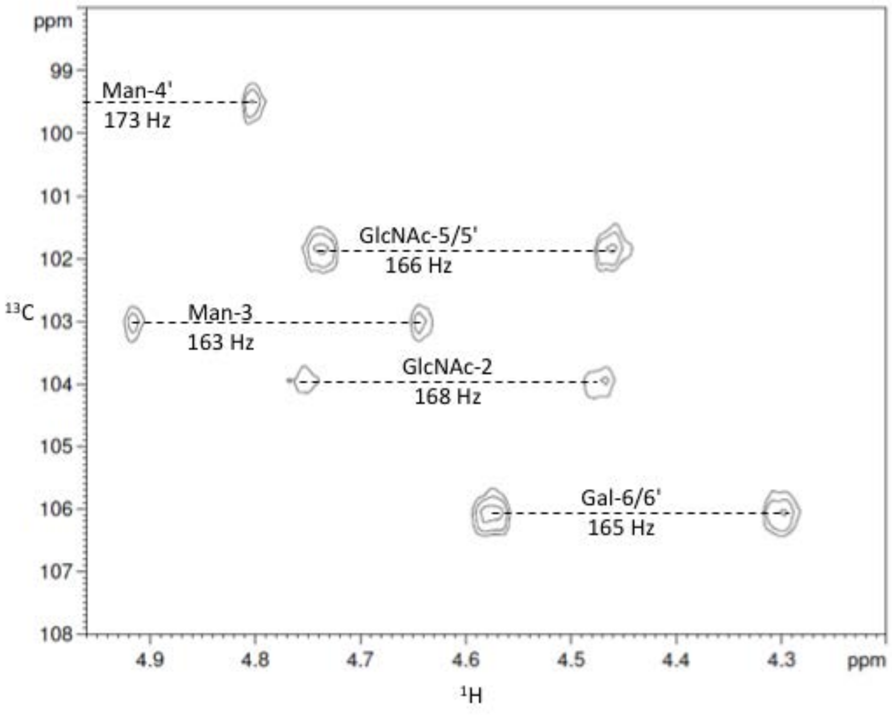

NMR spectra of glycan-transferred *p*-nitrophenyl mannose 9b

(A) 1D ^{1}H-NMR spectrum

(B) 1D selective TOCSY (mixing time = 200 ms) inverting H1 signal of Man-1

(C) 2D DQF-COSY

(D) 2D ^{1}H-^{13}C HSQC (black, upper and lower panels) and 2H ^{1}H-^{13}C constant-time HMBC (red, lower panel) spectra of glycan-transferred *p*NP-mannose **9b**

(E) 2D ^{13}C-coupled ^{1}H-^{13}C HSQC spectrum (anomeric region) of glycan-transferred *p*NP-mannose. $^{1}J_{CH}$ values are labeled for each anomeric signal. ^{3}J(H1, H2) of GlcNAc-2 is 8.8 Hz.

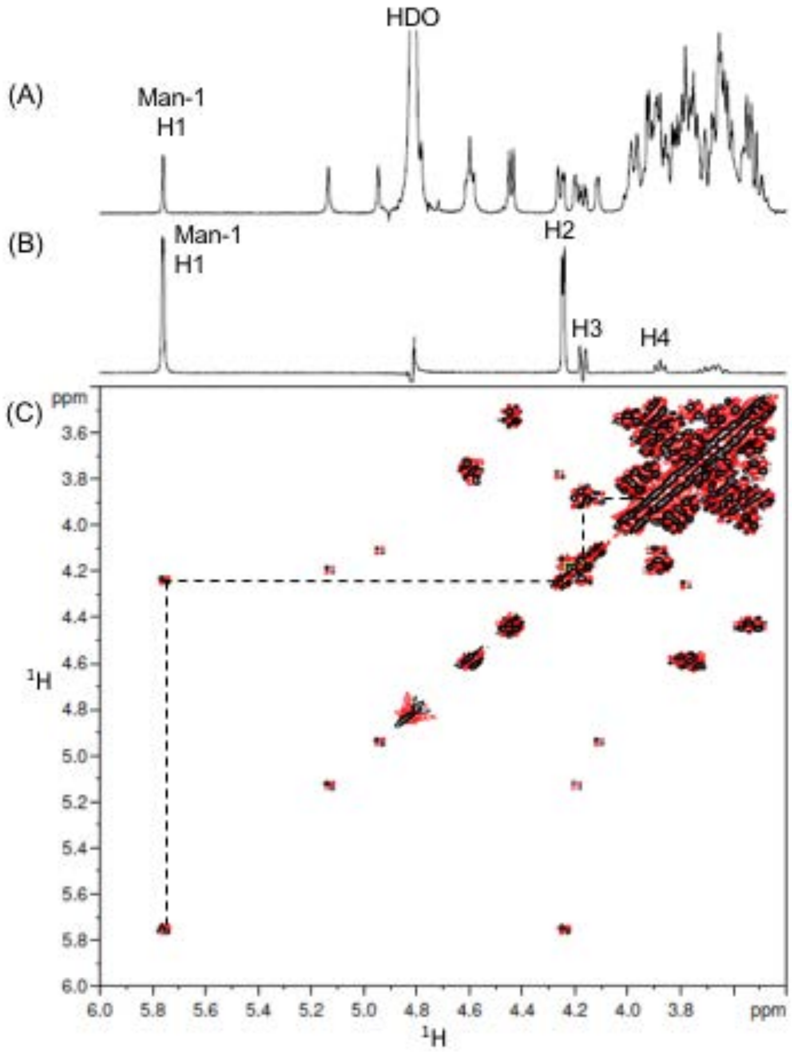

(D)

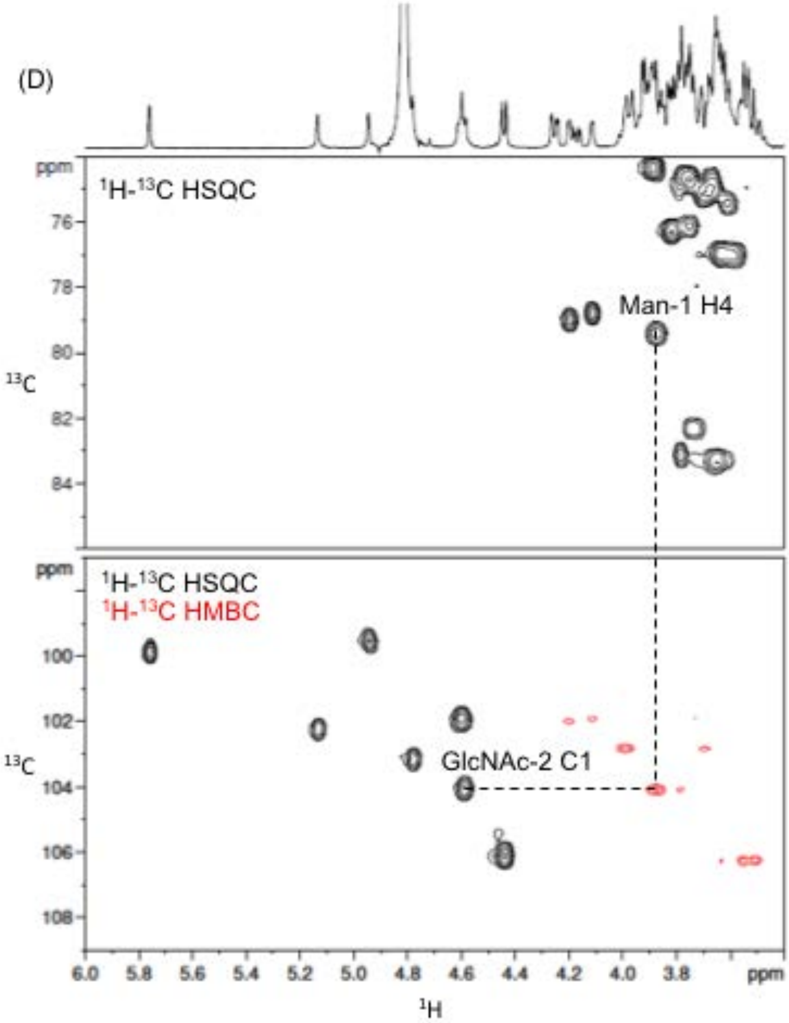

ppm
1H-13C HSQC
76
78
80
82
84
13C
Man-1 H4
ppm
1H-13C HSQC
1H-13C HMBC
100
102
104
106
108
13C
GlcNAc-2 C1
6.0 5.8 5.6 5.4 5.2 5.0 4.8 4.6 4.4 4.2 4.0 3.8 ppm
1H

(E)

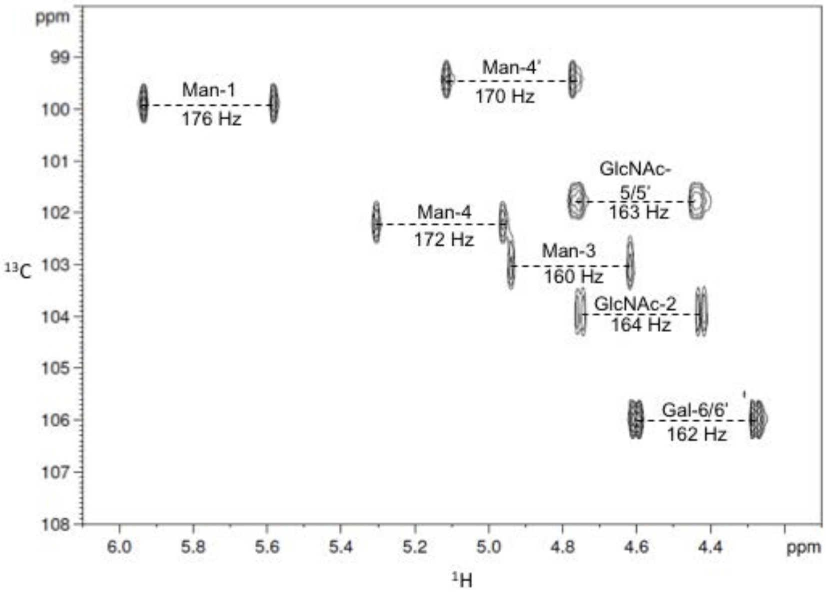

ppm
99
100
101
102
103
104
105
106
107
108
13C
Man-1
176 Hz
Man-4'
170 Hz
GlcNAc-
5/5'
163 Hz
Man-4
172 Hz
Man-3
160 Hz
GlcNAc-2
164 Hz
Gal-6/6'
162 Hz
6.0 5.8 5.6 5.4 5.2 5.0 4.8 4.6 4.4 ppm
1H

Time course of glycan transfer reaction mediated endo-CC N180H on pH difference.

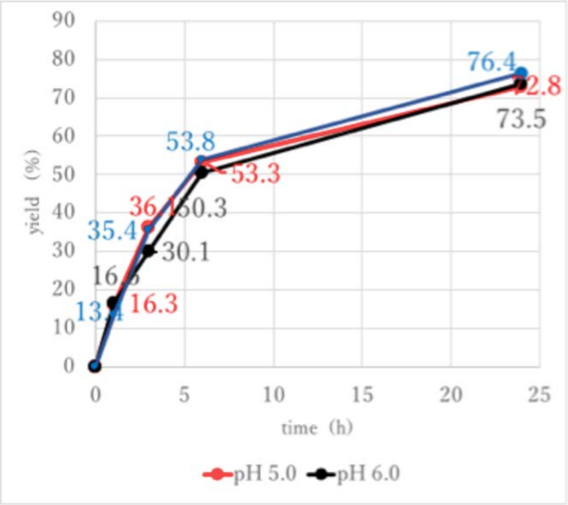

Time-course of preparation of **4b.**

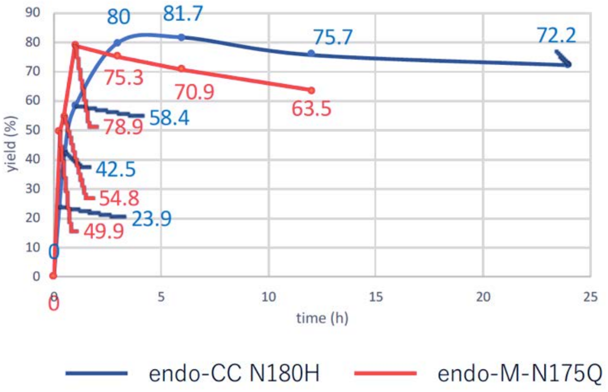

detection 214, 280 nm: r.t. 23.2 min.

Time-course of preparation of **5b**

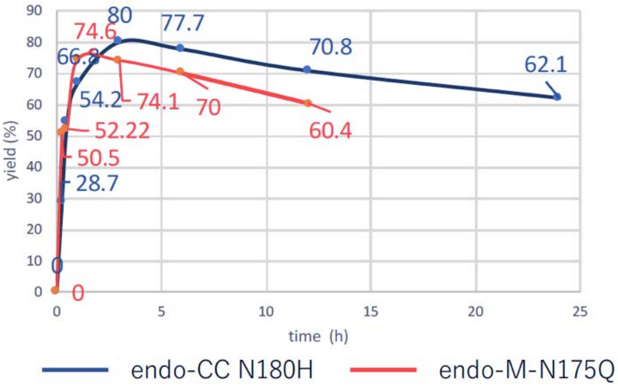

detection 214, 280 nm: r.t. 25.6 min.

Time-course of preparation of **6b**

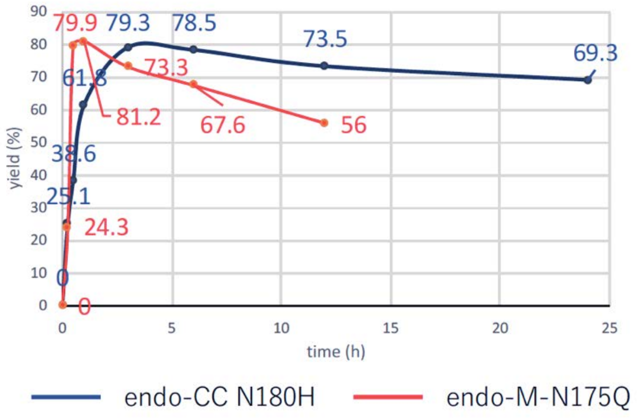

detection 214, 301 nm: r.t. 28.9 nm.

Time-course of preparation of **7b**

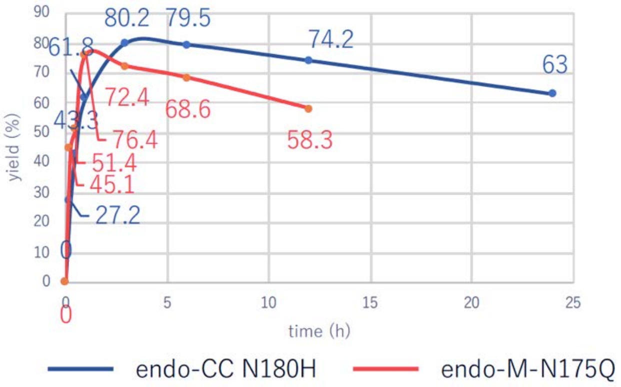

detection 214, 301 nm: r.t. 23.5 min.

Time-course of preparation of **8b.**

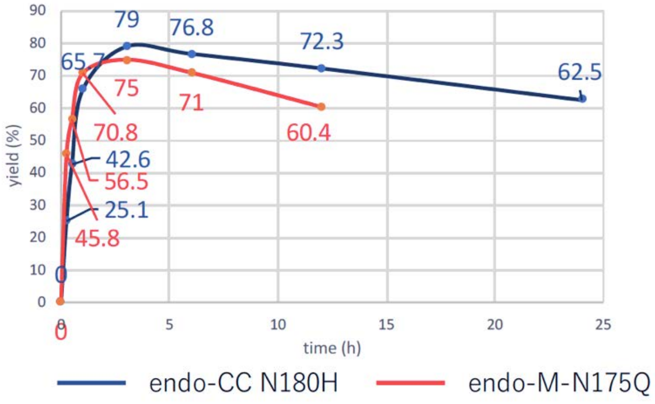

detection 214, 280 nm: r.t. 22.8 min.

Time-course of preparation of **9b.**

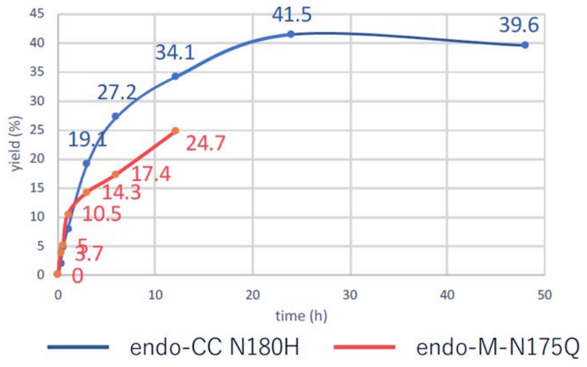

detection 214, 280 nm: r.t. 23.7 min.

Time-course of preparation of **15b.**

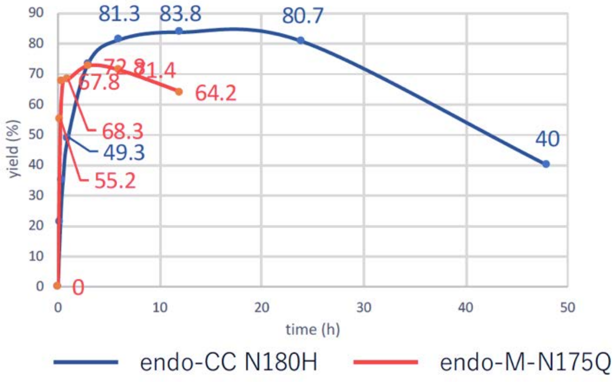

detection 214 nm, 280 nm: r.t. 22.3 min.

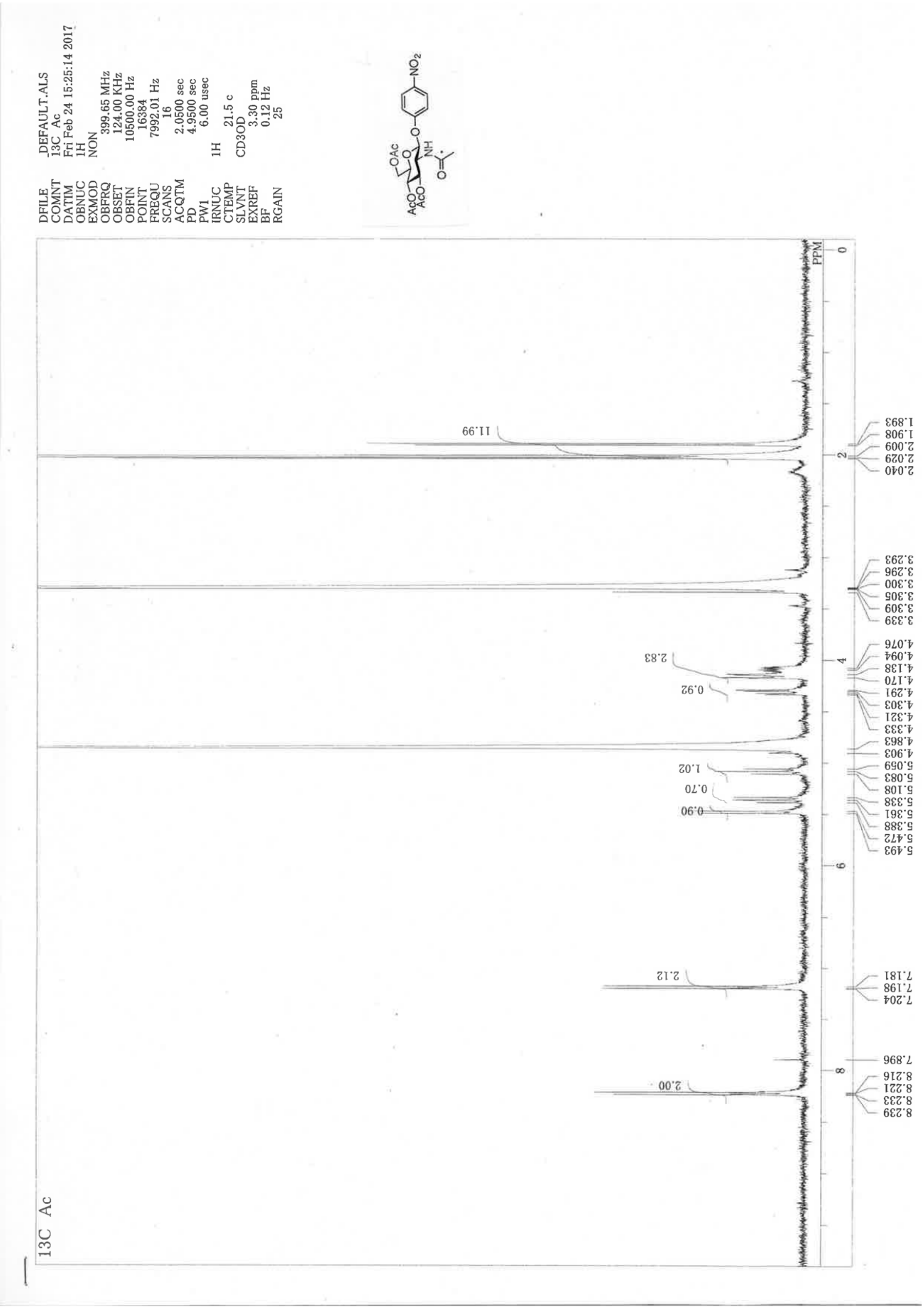

DFILE _DEFAULT.ALS
COMNT 13C Ac
DATIM Fri Feb 24 15:25:14 2017
OBNUC 1H
EXMOD NON
OBFRQ 399.65 MHz
OBSET 124.00 KHz
OBFIN 10500.00 Hz
POINT 16384
FREQU 7992.01 Hz
SCANS 16
ACQTM 2.0500 sec
PD 4.9500 sec
PW1 6.00 usec
IRNUC 1H
CTEMP 21.5 c
SLVNT CD3OD
EXREF 3.30 ppm
BF 0.12 Hz
RGAIN 25
AcO
AcO
OAc
O
NH
O
NO2
13C Ac
PPM
11.99
2.83
0.92
1.02
0.70
0.90
2.12
2.00
1.893
1.908
2.009
2.029
2.040
3.293
3.296
3.300
3.305
3.309
3.339
4.076
4.094
4.138
4.170
4.291
4.303
4.321
4.333
4.863
4.903
5.059
5.083
5.108
5.338
5.361
5.388
5.472
5.493
7.181
7.198
7.204
7.896
8.216
8.221
8.233
8.239
0
2
4
6
8

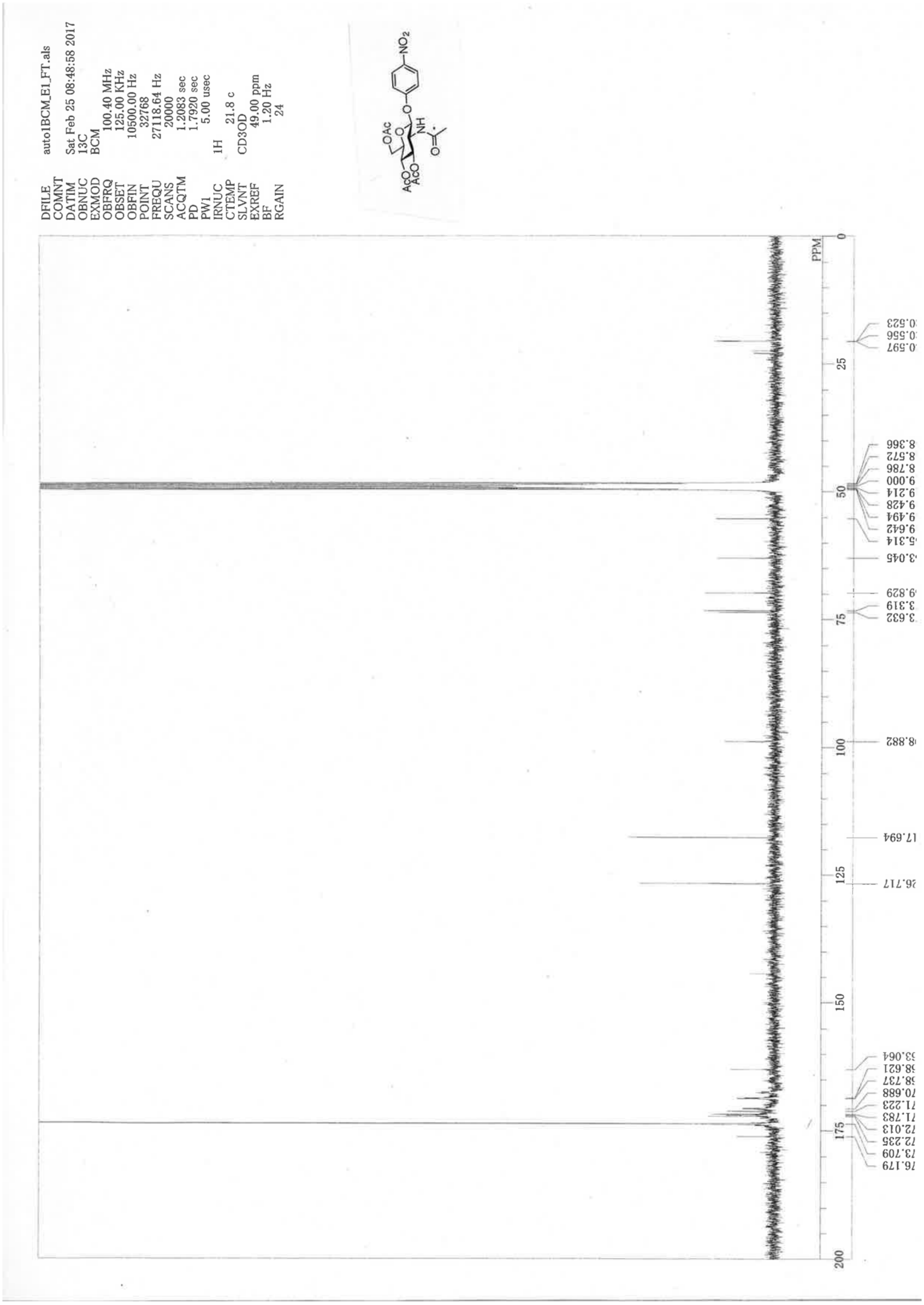

DFILE auto1BCM_E1_FT.als
COMNT
DATIM Sat Feb 25 08:48:58 2017
OBNUC 13C
EXMOD BCM
OBFRQ 100.40 MHz
OBSET 125.00 KHz
OBFIN 10500.00 Hz
POINT 32768
FREQU 27118.64 Hz
SCANS 20000
ACQTM 1.2083 sec
PD 1.7920 sec
PW1 5.00 usec
IRNUC 1H
CTEMP 21.8 c
SLVNT CD3OD
EXREF 49.00 ppm
BF 1.20 Hz
RGAIN 24
PPM

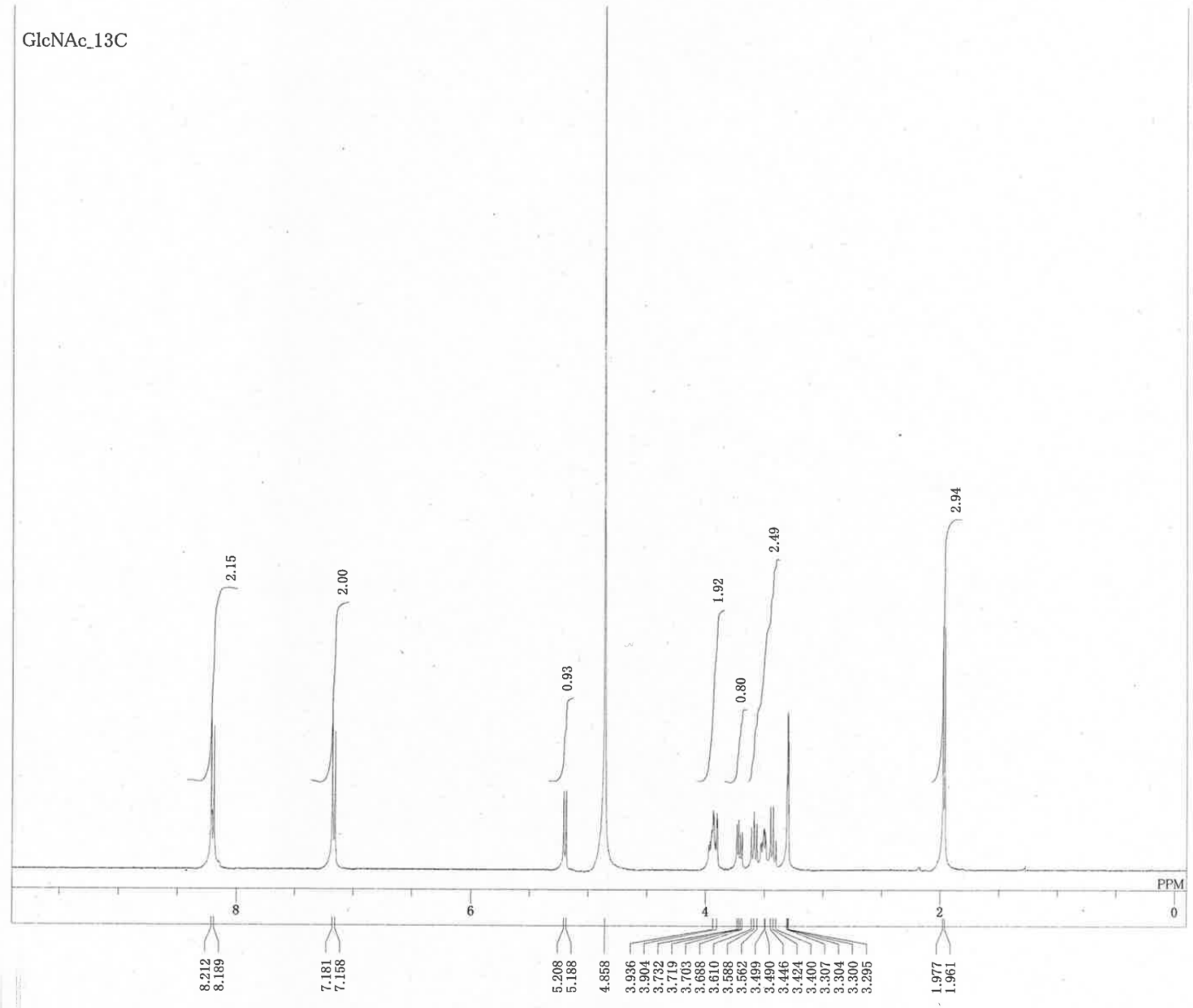

DFILE	_DEFAULT.ALS
COMNT	GlcNAc_13C
DATIM	Mon Jun 05 14:17:38 2017
OBNUC	1H
EXMOD	NON
OBFRQ	399.65 MHz
OBSET	124.00 KHz
OBFIN	10500.00 Hz
POINT	16384
FREQU	7992.01 Hz
SCANS	16
ACQTM	2.0500 sec
PD	4.9500 sec
PW1	6.00 usec
IRNUC	1H
CTEMP	22.0 c
SLVNT	CD3OD
EXREF	3.30 ppm
BF	0.12 Hz
RGAIN	23

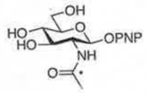

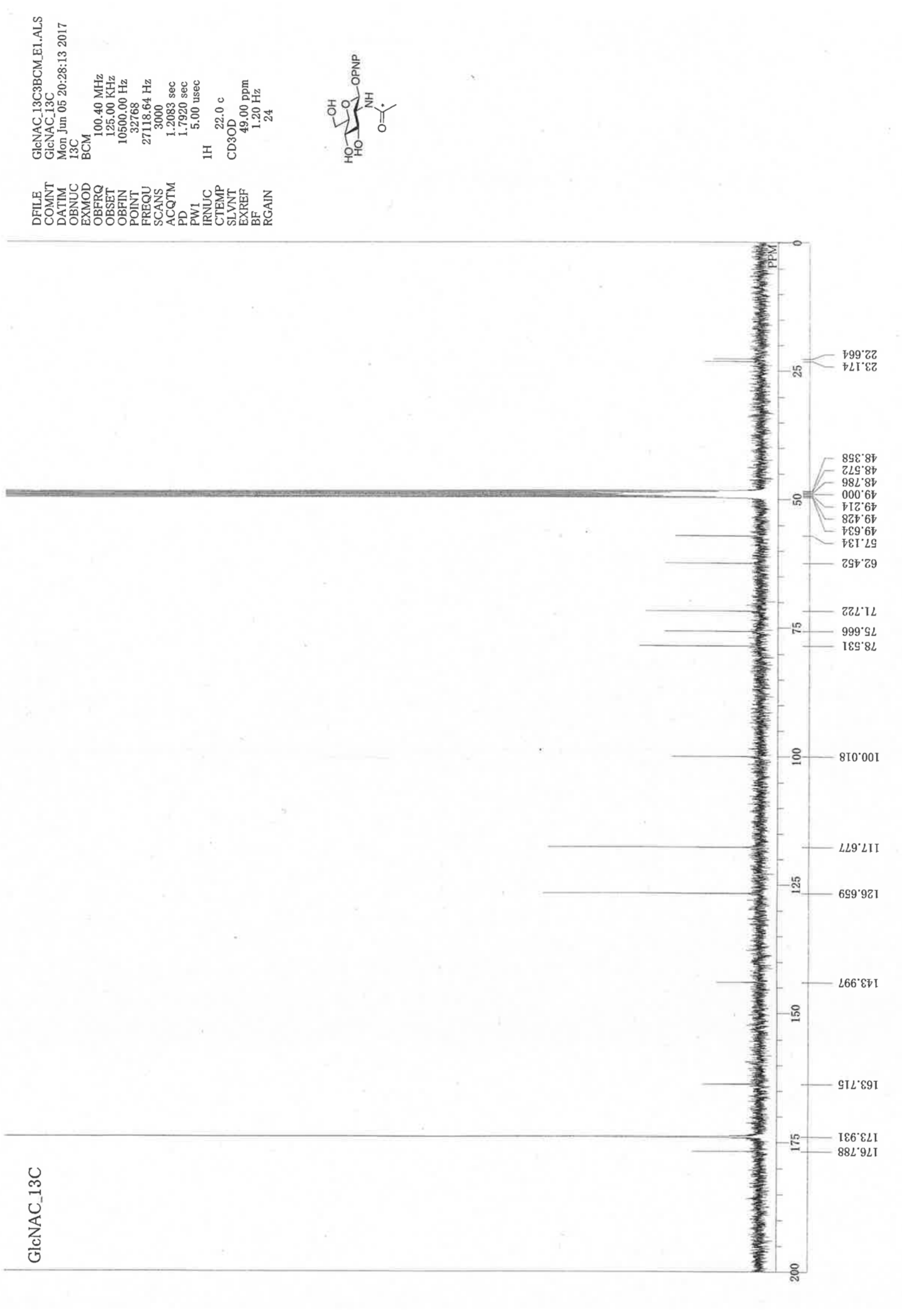
GlcNAC_13C
DFILE GlcNAC_13C3BCM_E1.ALS
COMNT GlcNAC_13C
DATIM Mon Jun 05 20:28:13 2017
OBNUC 13C
EXMOD BCM
OBFRQ 100.40 MHz
OBSET 125.00 KHz
OBFIN 10500.00 Hz
POINT 32768
FREQU 27118.64 Hz
SCANS 3000
ACQTM 1.2083 sec
PD 1.7920 sec
PW1 5.00 usec
IRNUC 1H
CTEMP 22.0 c
SLVNT CD3OD
EXREF 49.00 ppm
BF 1.20 Hz
RGAIN 24
HO
HO
OH
O
OPNP
NH
O
PPM
200
175
150
125
100
75
50
25
0
176.788
173.931
163.715
143.997
126.659
117.677
100.018
78.531
75.666
71.722
62.452
57.134
49.634
49.428
49.214
49.000
48.786
48.572
48.358
23.174
22.664

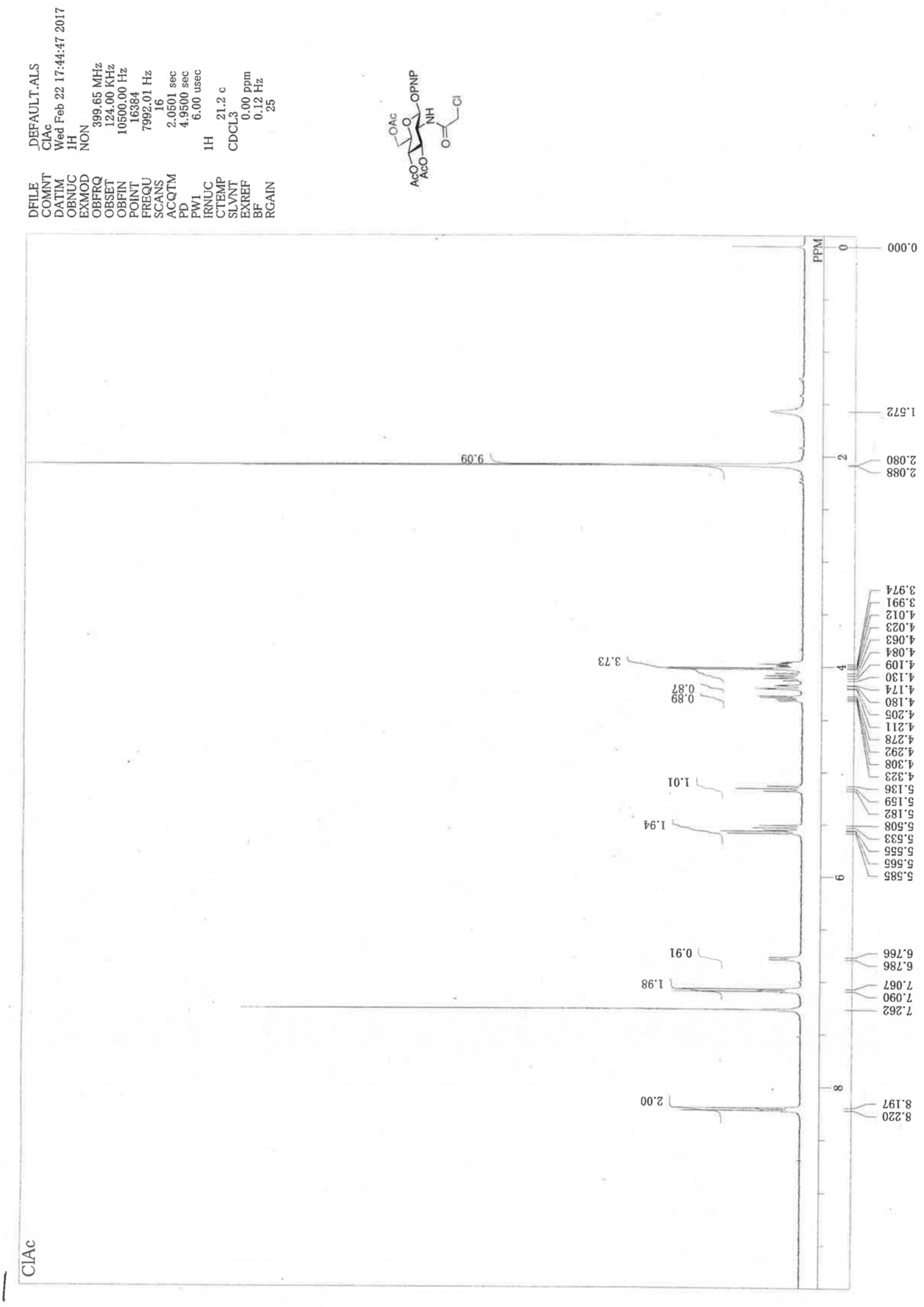
DFILE _DEFAULT.ALS
COMNT ClAc
DATIM Wed Feb 22 17:44:47 2017
OBNUC 1H
EXMOD NON
OBFRQ 399.65 MHz
OBSET 124.00 KHz
OBFIN 10500.00 Hz
POINT 16384
FREQU 7992.01 Hz
SCANS 16
ACQTM 2.0501 sec
PD 4.9500 sec
PW1 6.00 usec
IRNUC 1H
CTEMP 21.2 c
SLVNT CDCL3
EXREF 0.00 ppm
BF 0.12 Hz
RGAIN 25
OAc
AcO
AcO
O
OPNP
NH
O
Cl
ClAc
PPM
0
2
4
6
8
9.09
3.73
0.87
0.89
1.01
1.94
0.91
1.98
2.00
0.000
1.572
2.080
2.088
3.974
3.991
4.012
4.023
4.063
4.084
4.109
4.130
4.174
4.180
4.205
4.211
4.278
4.292
4.308
4.323
5.136
5.159
5.182
5.508
5.533
5.555
5.565
5.585
6.766
6.786
7.067
7.090
7.262
8.197
8.220

DFILE auto1BCM_E1_FT.als
COMNT
DATIM Thu Feb 23 07:36:18 2017
OBNUC 13C
EXMOD BCM
OBFRQ 100.40 MHz
OBSET 125.00 KHz
OBFIN 10500.00 Hz
POINT 32768
FREQU 27118.64 Hz
SCANS 15000
ACQTM 1.2083 sec
PD 1.7920 sec
PW1 5.00 usec
IRNUC 1H
CTEMP 22.0 c
SLVNT CDCL3
EXREF 77.00 ppm
BF 1.20 Hz
RGAIN 25

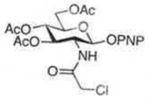

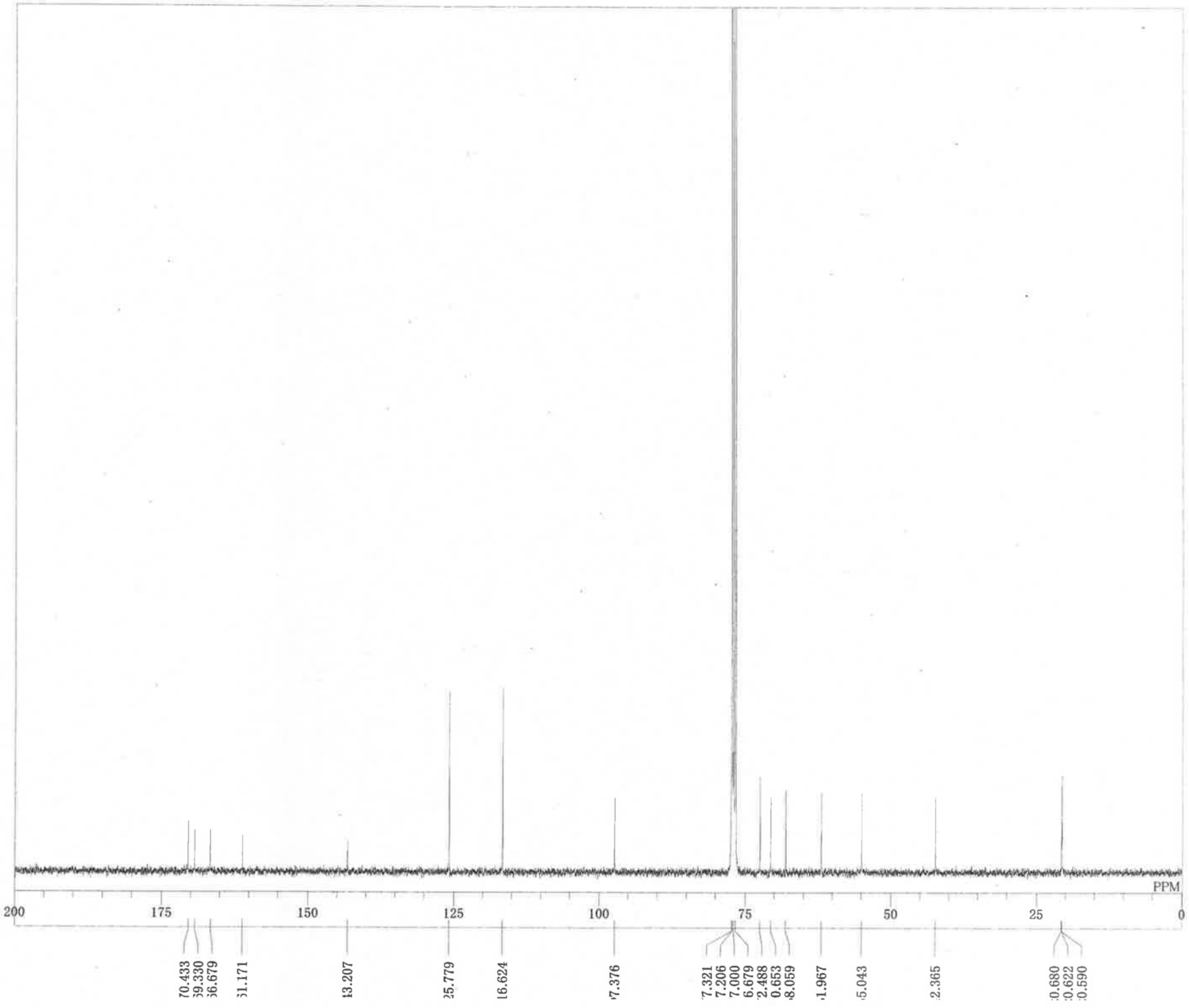

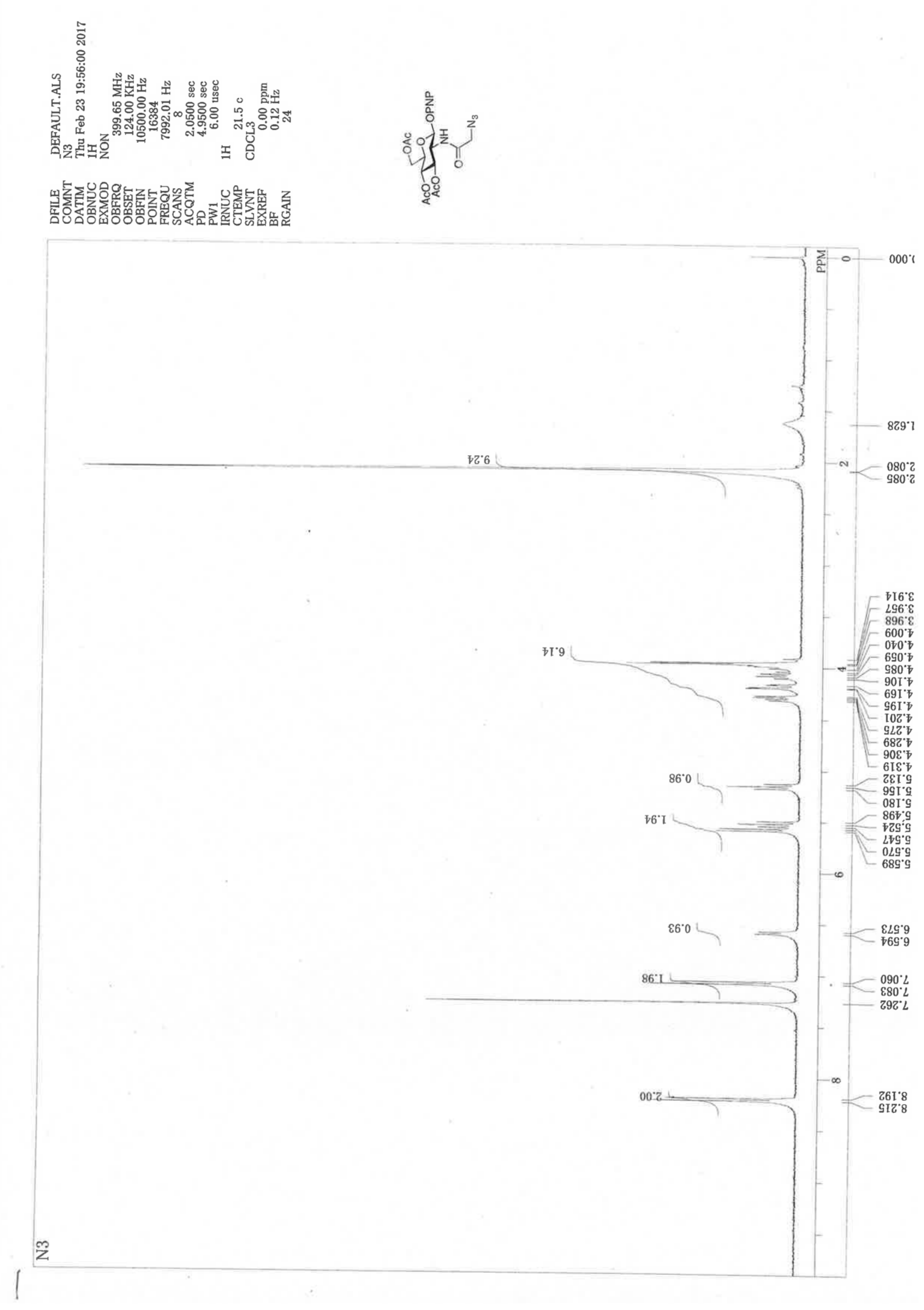
DFILE _DEFAULT.ALS
COMNT N3
DATIM Thu Feb 23 19:56:00 2017
OBNUC 1H
EXMOD NON
OBFRQ 399.65 MHz
OBSET 124.00 KHz
OBFIN 10500.00 Hz
POINT 16384
FREQU 7992.01 Hz
SCANS 8
ACQTM 2.0500 sec
PD 4.9500 sec
PW1 6.00 usec
IRNUC 1H
CTEMP 21.5 c
SLVNT CDCL3
EXREF 0.00 ppm
BF 0.12 Hz
RGAIN 24
OAc
AcO
AcO
O
OPNP
NH
O
N_3
N3
PPM
0
2
4
6
8
9.24
6.14
0.98
1.94
0.93
1.98
2.00
0.000
1.628
2.080
2.085
3.914
3.957
3.968
4.009
4.040
4.059
4.085
4.106
4.169
4.195
4.201
4.275
4.289
4.306
4.319
5.132
5.156
5.180
5.498
5.524
5.547
5.570
5.589
6.573
6.594
7.060
7.083
7.262
8.192
8.215

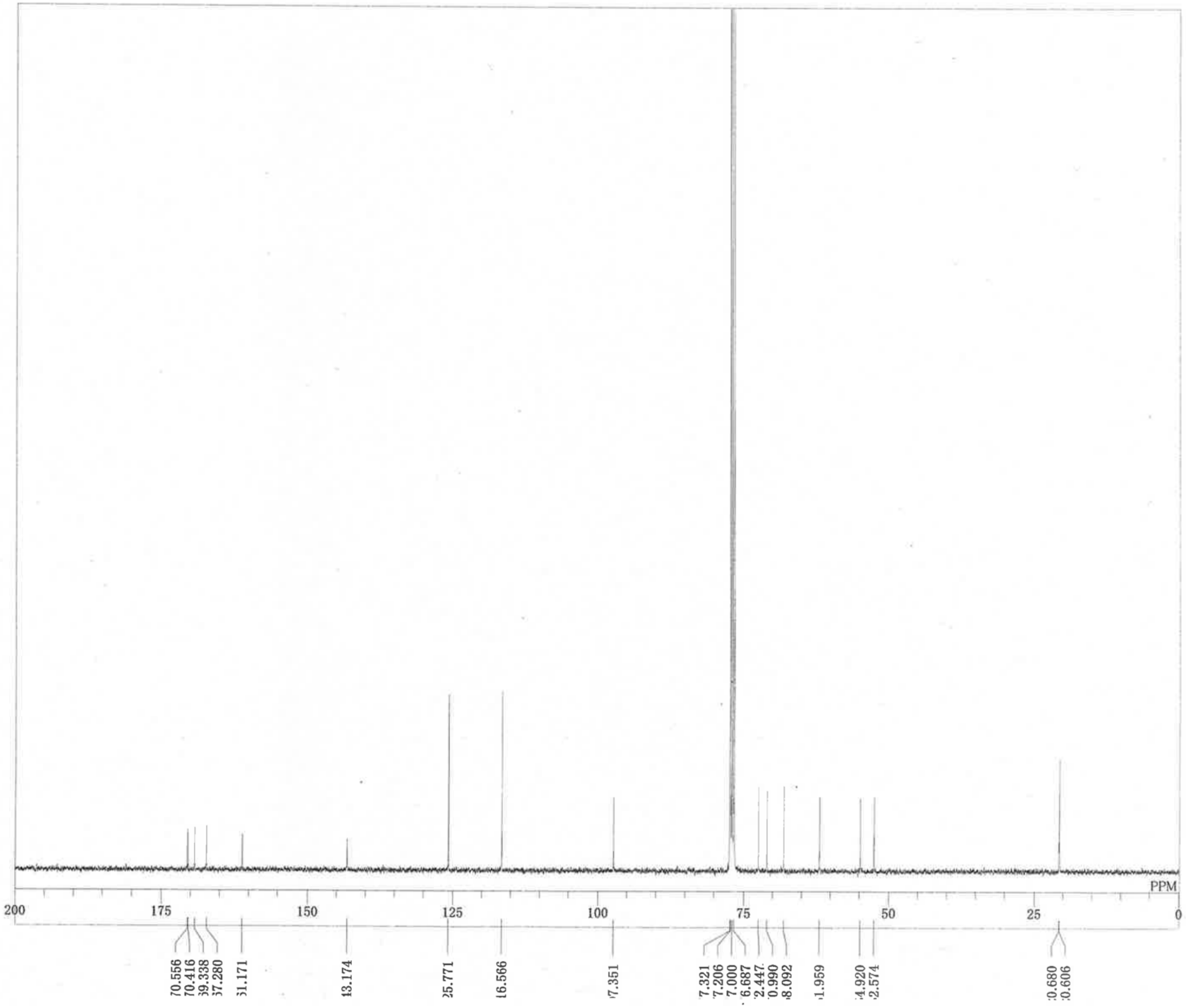

DFILE	auto1BCM_E1.ALS
COMNT	
DATIM	Fri Feb 24 08:35:37 2017
OBNUC	13C
EXMOD	BCM
OBFRQ	100.40 MHz
OBSET	125.00 KHz
OBFIN	10500.00 Hz
POINT	32768
FREQU	27118.64 Hz
SCANS	15000
ACQTM	1.2083 sec
PD	1.7920 sec
PW1	5.00 usec
IRNUC	1H
CTEMP	21.4 c
SLVNT	CDCL3
EXREF	77.00 ppm
BF	1.20 Hz
RGAIN	26

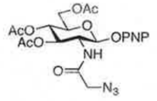

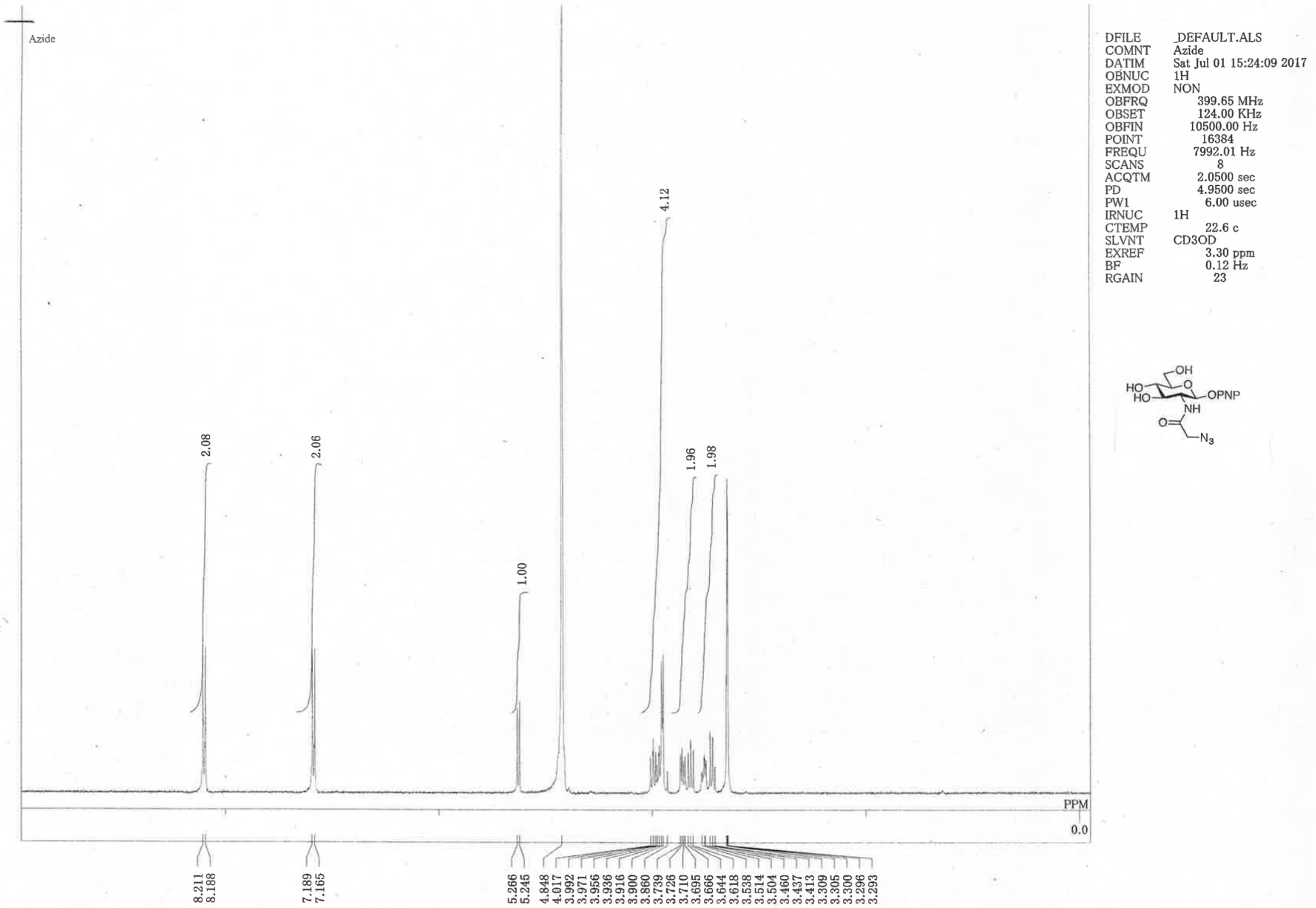

Azide
DFILE _DEFAULT.ALS
COMNT Azide
DATIM Sat Jul 01 15:24:09 2017
OBNUC 1H
EXMOD NON
OBFRQ 399.65 MHz
OBSET 124.00 KHz
OBFIN 10500.00 Hz
POINT 16384
FREQU 7992.01 Hz
SCANS 8
ACQTM 2.0500 sec
PD 4.9500 sec
PW1 6.00 usec
IRNUC 1H
CTEMP 22.6 c
SLVNT CD3OD
EXREF 3.30 ppm
BF 0.12 Hz
RGAIN 23
HO
HO
OH
O
OPNP
NH
O
N_3
2.08
2.06
1.00
4.12
1.96
1.98
PPM
0.0
8.211
8.188
7.189
7.165
5.266
5.245
4.848
4.017
3.992
3.971
3.956
3.936
3.916
3.900
3.860
3.739
3.726
3.710
3.695
3.666
3.644
3.618
3.538
3.514
3.504
3.460
3.437
3.413
3.309
3.305
3.300
3.296
3.293

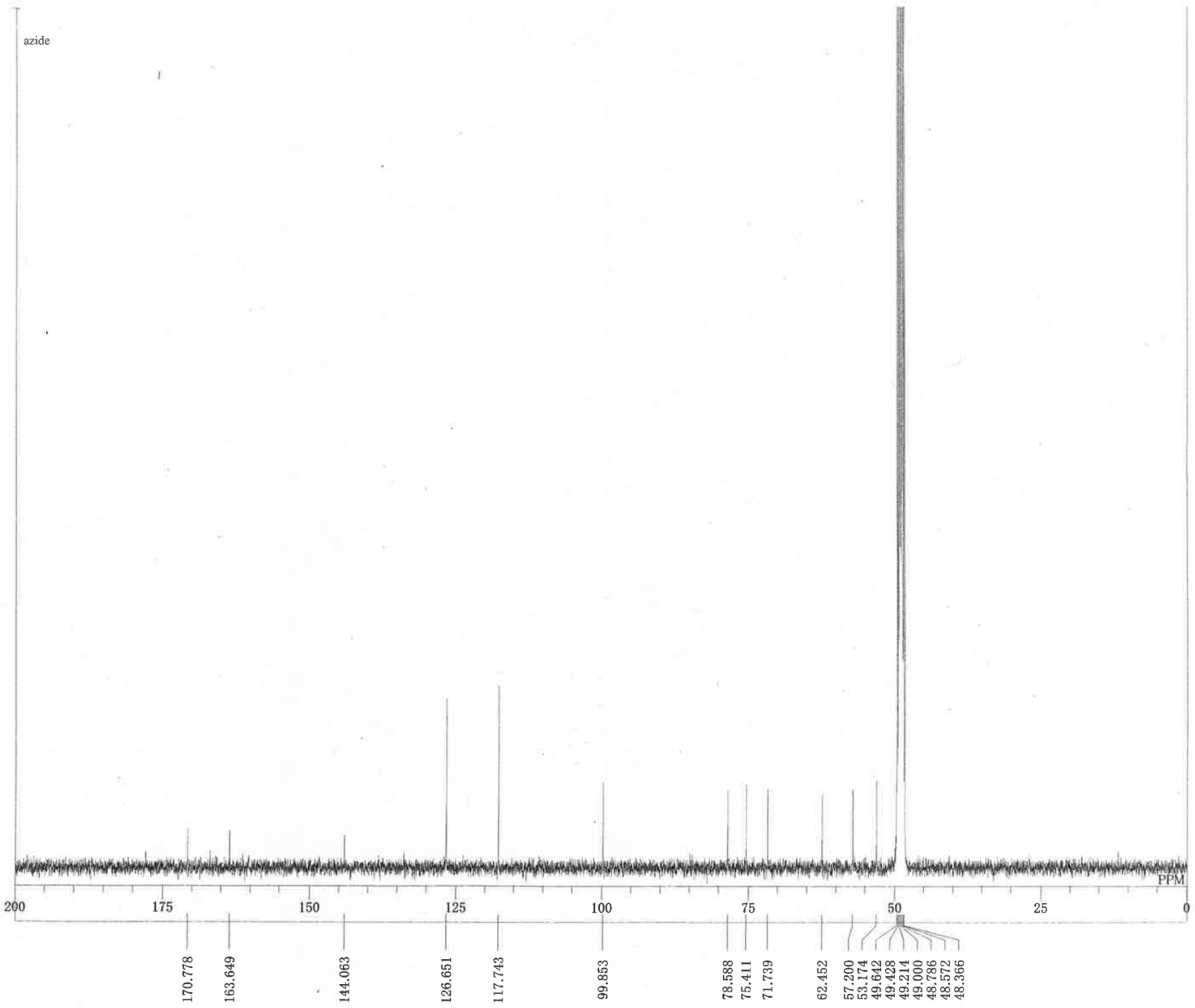

DFILE auto1BCM_E1_FT.als
COMNT azide
DATIM Sun Jul 02 03:52:49 2017
OBNUC 13C
EXMOD BCM
OBFRQ 100.40 MHz
OBSET 125.00 KHz
OBFIN 10500.00 Hz
POINT 32768
FREQU 27118.64 Hz
SCANS 10000
ACQTM 1.2083 sec
PD 1.7920 sec
PW1 5.00 usec
IRNUC 1H
CTEMP 23.6 c
SLVNT CD3OD
EXREF 49.00 ppm
BF 1.20 Hz
RGAIN 24

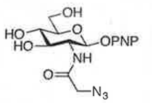

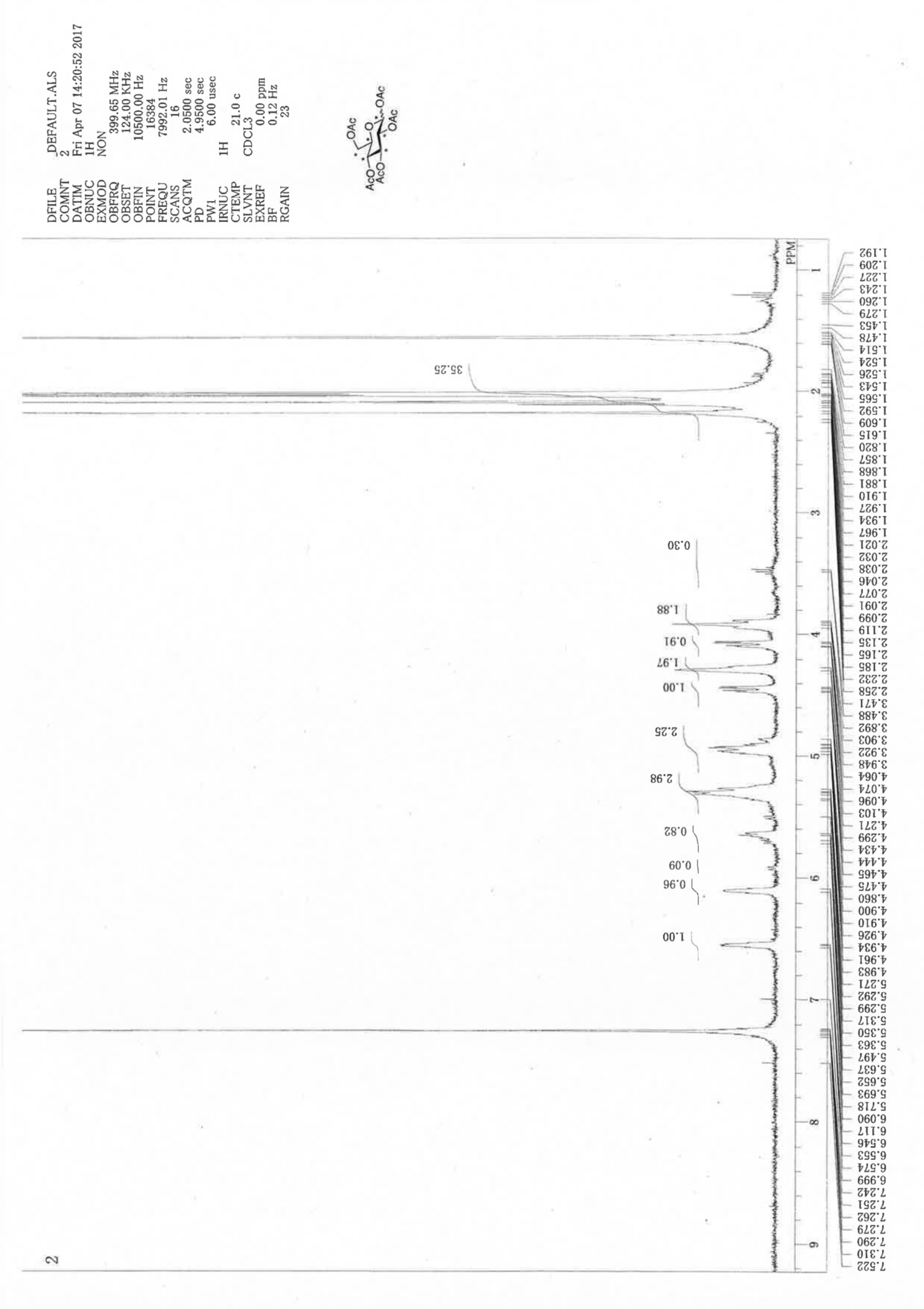
DFILE DEFAULT.ALS
COMNT 2
DATIM Fri Apr 07 14:20:52 2017
OBNUC 1H
EXMOD NON
OBFRQ 399.65 MHz
OBSET 124.00 KHz
OBFIN 10500.00 Hz
POINT 16384
FREQU 7992.01 Hz
SCANS 16
ACQTM 2.0500 sec
PD 4.9500 sec
PW1 6.00 usec
IRNUC 1H
CTEMP 21.0 c
SLVNT CDCL3
EXREF 0.00 ppm
BF 0.12 Hz
RGAIN 23
PPM

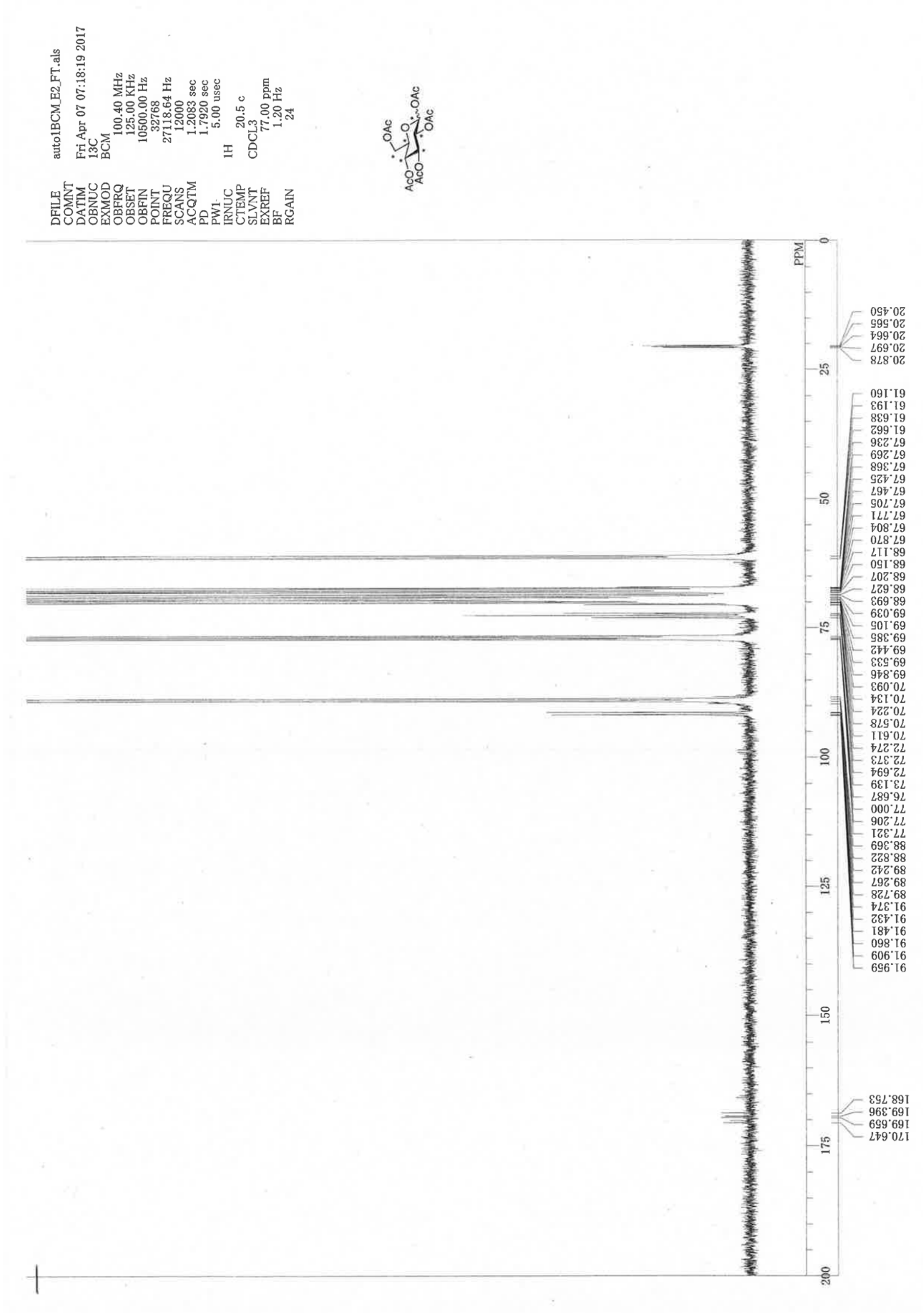
DFILE auto1BCM_E2_FT.als
COMNT
DATIM Fri Apr 07 07:18:19 2017
OBNUC 13C
EXMOD BCM
OBFRQ 100.40 MHz
OBSET 125.00 KHz
OBFIN 10500.00 Hz
POINT 32768
FREQU 27118.64 Hz
SCANS 12000
ACQTM 1.2083 sec
PD 1.7920 sec
PW1 5.00 usec
IRNUC 1H
CTEMP 20.5 c
SLVNT CDCL3
EXREF 77.00 ppm
BF 1.20 Hz
RGAIN 24
AcO
AcO
OAc
O
OAc
OAc
PPM
0
25
50
75
100
125
150
175
200
20.450
20.565
20.664
20.697
20.878
61.160
61.193
61.638
61.662
67.236
67.269
67.368
67.425
67.467
67.705
67.771
67.804
67.870
68.117
68.150
68.207
68.627
68.693
69.039
69.105
69.386
69.442
69.533
69.846
70.093
70.134
70.224
70.578
70.611
72.274
72.373
72.694
73.139
76.687
77.000
77.206
77.321
88.369
88.822
89.242
89.267
89.728
91.374
91.432
91.481
91.860
91.909
91.959
168.753
169.396
169.659
170.647

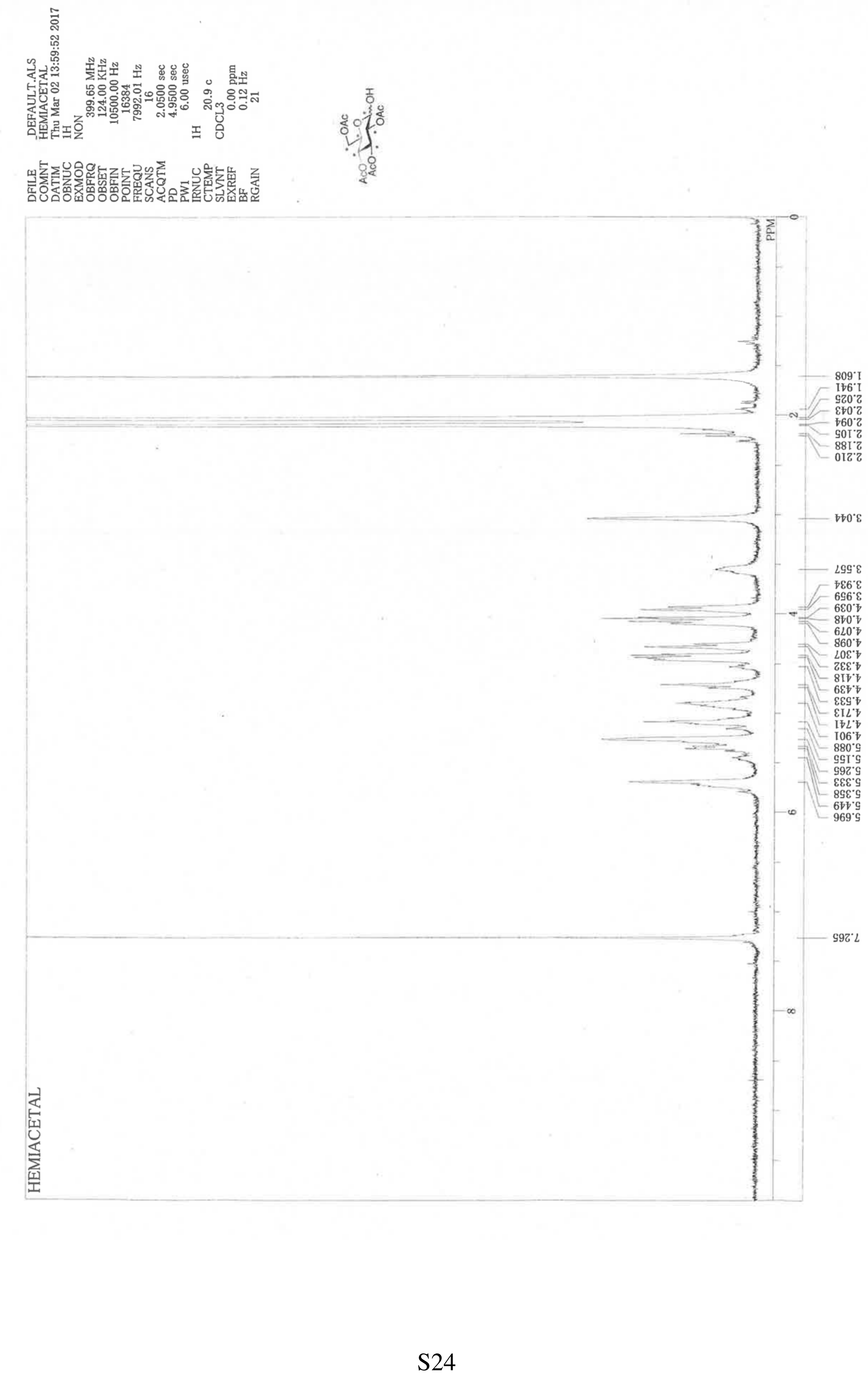
DFILE _DEFAULT.ALS
COMNT HEMIACETAL
DATIM Thu Mar 02 13:59:52 2017
OBNUC 1H
EXMOD NON
OBFRQ 399.65 MHz
OBSET 124.00 KHz
OBFIN 10500.00 Hz
POINT 16384
FREQU 7992.01 Hz
SCANS 16
ACQTM 2.0500 sec
PD 4.9500 sec
PW1 6.00 usec
IRNUC 1H
CTEMP 20.9 c
SLVNT CDCL3
EXREF 0.00 ppm
BF 0.12 Hz
RGAIN 21
AcO
AcO
OAc
OAc
OH
HEMIACETAL
PPM
0
2
4
6
8
1.608
1.941
2.025
2.043
2.094
2.105
2.188
2.210
3.044
3.557
3.934
3.959
4.039
4.048
4.079
4.098
4.307
4.332
4.418
4.439
4.533
4.713
4.741
4.901
5.088
5.155
5.265
5.333
5.358
5.449
5.696
7.265

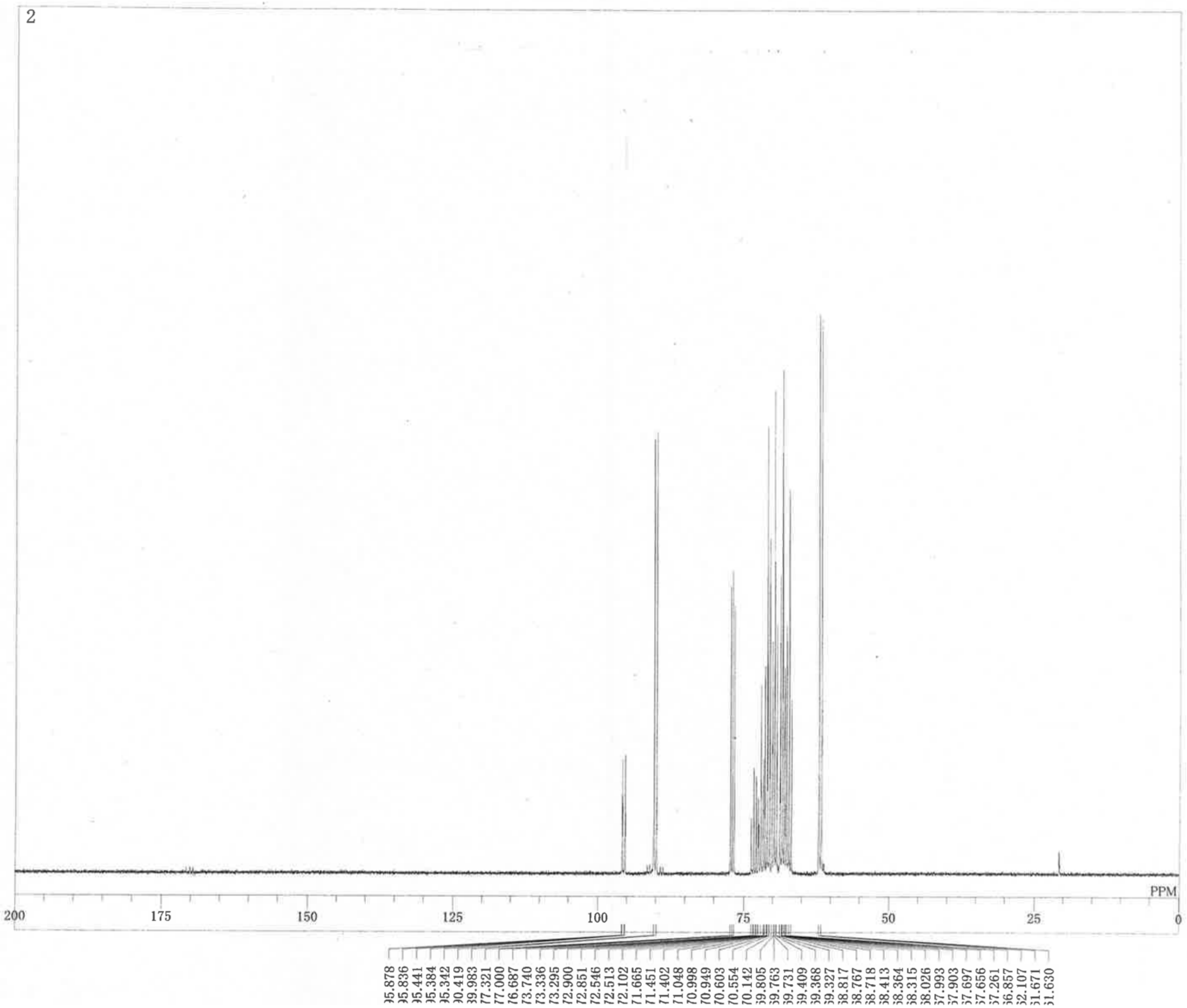

DFILE	_DEFAULT.ALS
COMNT	2
DATIM	Thu Mar 02 17:59:58 2017
OBNUC	13C
EXMOD	BCM
OBFRQ	100.40 MHz
OBSET	125.00 KHz
OBFIN	10500.00 Hz
POINT	32768
FREQU	27118.64 Hz
SCANS	4607
ACQTM	1.2083 sec
PD	1.7920 sec
PW1	5.00 usec
IRNUC	1H
CTEMP	20.8 c
SLVNT	CDCL3
EXREF	77.00 ppm
BF	0.12 Hz
RGAIN	23

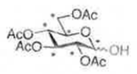

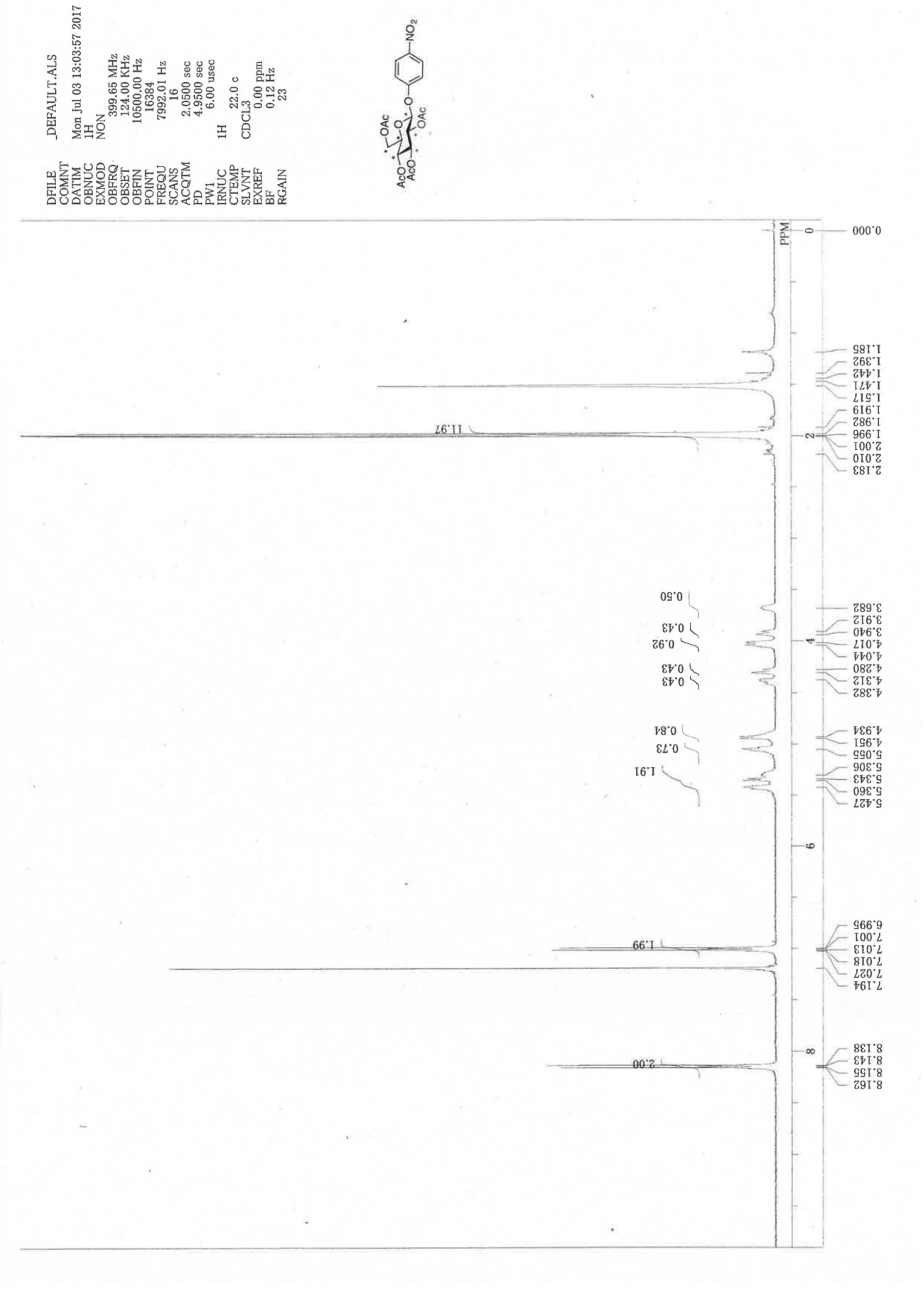
DFILE _DEFAULT.ALS
COMNT
DATIM Mon Jul 03 13:03:57 2017
OBNUC 1H
EXMOD NON
OBFRQ 399.65 MHz
OBSET 124.00 KHz
OBFIN 10500.00 Hz
POINT 16384
FREQU 7992.01 Hz
SCANS 16
ACQTM 2.0500 sec
PD 4.9500 sec
PW1 6.00 usec
IRNUC 1H
CTEMP 22.0 c
SLVNT CDCL3
EXREF 0.00 ppm
BF 0.12 Hz
RGAIN 23
AcO
AcO
OAc
OAc
NO_2
PPM
0
2
4
6
8
0.000
1.185
1.392
1.442
1.471
1.517
1.919
1.982
1.996
2.001
2.010
2.183
3.682
3.912
3.940
4.017
4.044
4.280
4.312
4.382
4.934
4.951
5.055
5.306
5.343
5.360
5.427
6.995
7.001
7.013
7.018
7.027
7.194
8.138
8.143
8.155
8.162
11.97
0.50
0.43
0.92
0.43
0.43
0.84
0.73
1.91
1.99
2.00

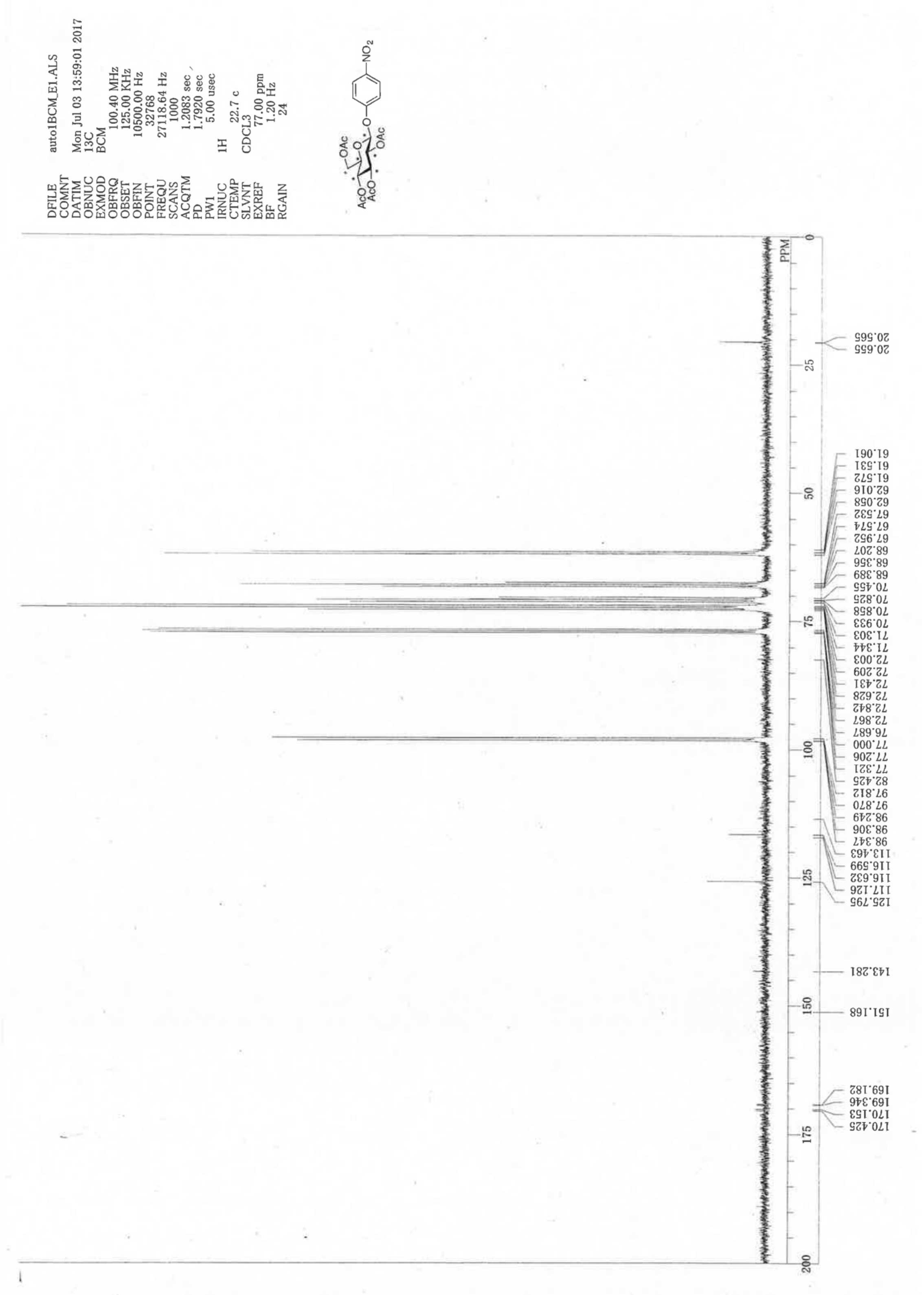
DFILE auto1BCM_E1.ALS
COMNT
DATIM Mon Jul 03 13:59:01 2017
OBNUC 13C
EXMOD BCM
OBFRQ 100.40 MHz
OBSET 125.00 KHz
OBFIN 10500.00 Hz
POINT 32768
FREQU 27118.64 Hz
SCANS 1000
ACQTM 1.2083 sec
PD 1.7920 sec
PW1 5.00 usec
IRNUC 1H
CTEMP 22.7 c
SLVNT CDCL3
EXREF 77.00 ppm
BF 1.20 Hz
RGAIN 24
AcO
AcO
OAc
OAc
NO_2
PPM
20.565
20.655
61.061
61.531
61.572
62.016
62.058
67.532
67.574
67.952
68.207
68.356
68.389
70.455
70.825
70.858
70.933
71.303
71.344
72.003
72.209
72.431
72.628
72.842
72.867
76.687
77.000
77.206
77.321
82.425
97.812
97.870
98.249
98.306
98.347
113.463
116.599
116.632
117.126
125.795
143.281
151.168
169.182
169.346
170.153
170.425
0
25
50
75
100
125
150
175
200

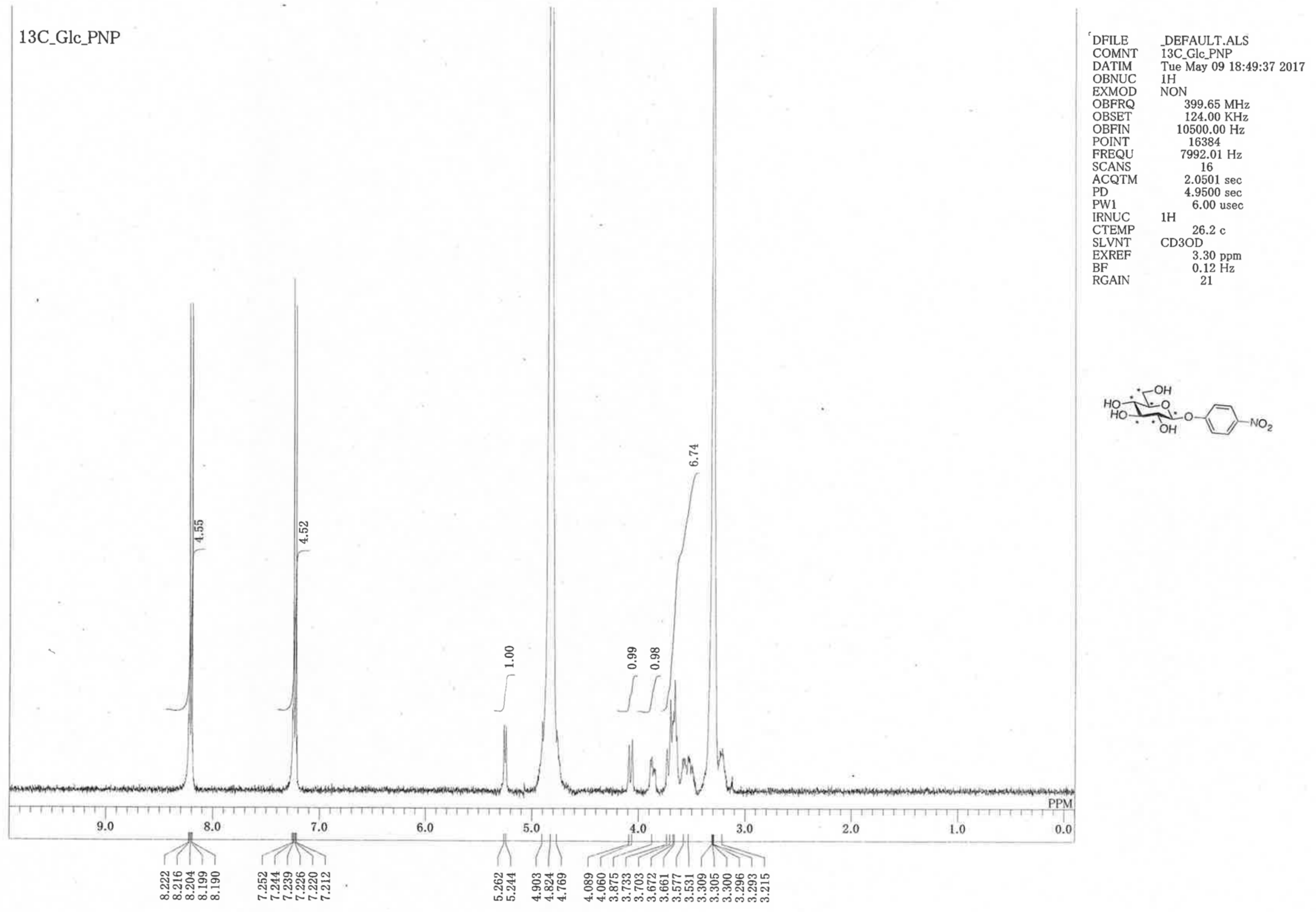
13C_Glc_PNP
DFILE _DEFAULT.ALS
COMNT 13C_Glc_PNP
DATIM Tue May 09 18:49:37 2017
OBNUC 1H
EXMOD NON
OBFRQ 399.65 MHz
OBSET 124.00 KHz
OBFIN 10500.00 Hz
POINT 16384
FREQU 7992.01 Hz
SCANS 16
ACQTM 2.0501 sec
PD 4.9500 sec
PW1 6.00 usec
IRNUC 1H
CTEMP 26.2 c
SLVNT CD3OD
EXREF 3.30 ppm
BF 0.12 Hz
RGAIN 21
HO
HO
OH
OH
O
NO2
4.55
4.52
1.00
0.99
0.98
6.74
PPM
9.0
8.0
7.0
6.0
5.0
4.0
3.0
2.0
1.0
0.0
8.222
8.216
8.204
8.199
8.190
7.252
7.244
7.239
7.226
7.220
7.212
5.262
5.244
4.903
4.824
4.769
4.089
4.060
3.875
3.733
3.703
3.672
3.661
3.577
3.531
3.309
3.305
3.300
3.296
3.293
3.215

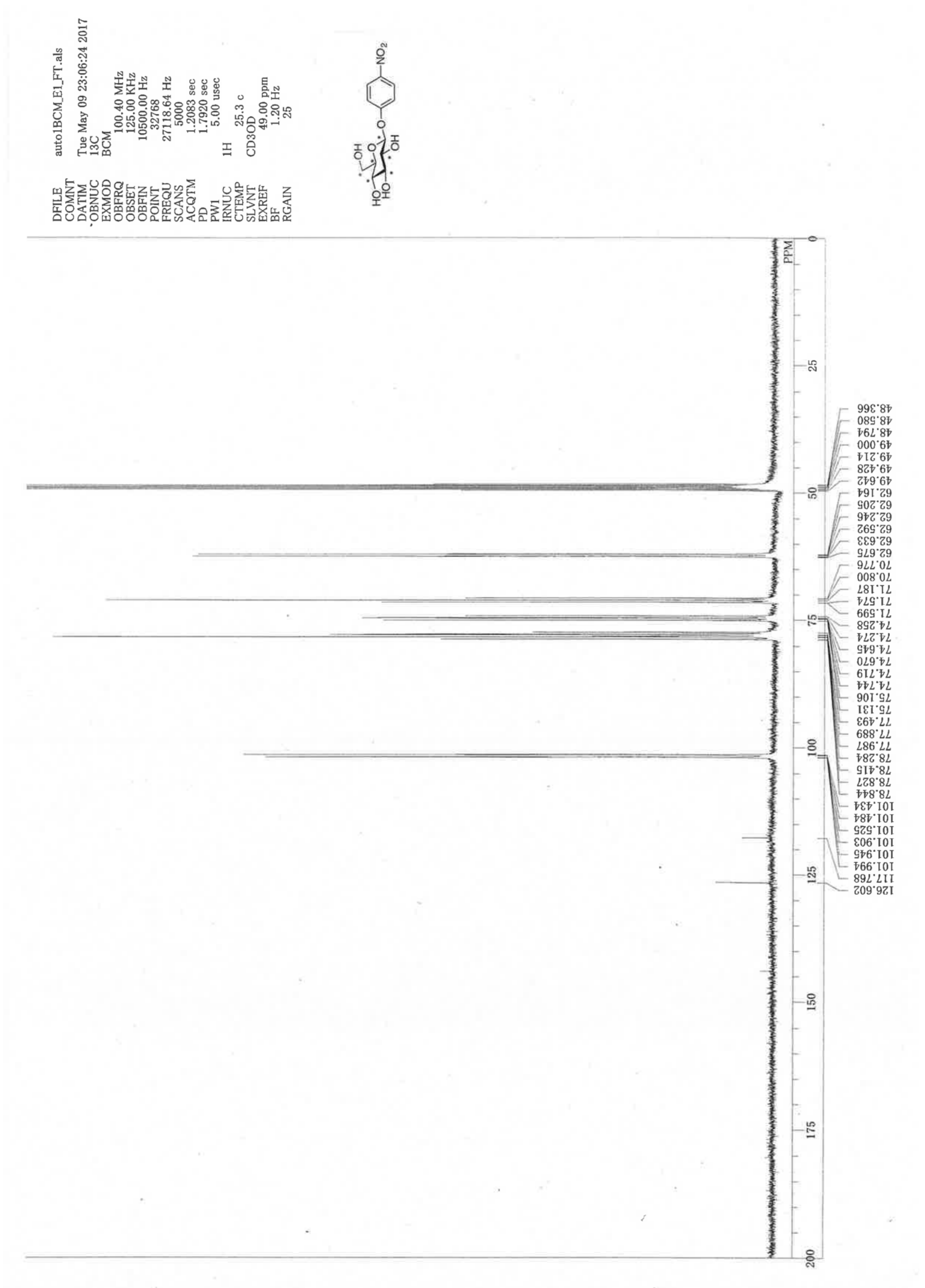
DFILE auto1BCM_E1_FT.als
COMNT
DATIM Tue May 09 23:06:24 2017
OBNUC 13C
EXMOD BCM
OBFRQ 100.40 MHz
OBSET 125.00 KHz
OBFIN 10500.00 Hz
POINT 32768
FREQU 27118.64 Hz
SCANS 5000
ACQTM 1.2083 sec
PD 1.7920 sec
PW1 5.00 usec
IRNUC 1H
CTEMP 25.3 c
SLVNT CD3OD
EXREF 49.00 ppm
BF 1.20 Hz
RGAIN 25
HO
HO
OH
O
O
OH
NO2
48.366
48.580
48.794
49.000
49.214
49.428
49.642
62.164
62.205
62.246
62.592
62.633
62.675
70.776
70.800
71.187
71.574
71.599
74.258
74.274
74.645
74.670
74.719
74.744
75.106
75.131
77.493
77.889
77.987
78.284
78.415
78.827
78.844
101.434
101.484
101.525
101.903
101.945
101.994
117.768
126.602
PPM
0
25
50
75
100
125
150
175
200

^{1}H-NMR spectrum of compound **3**

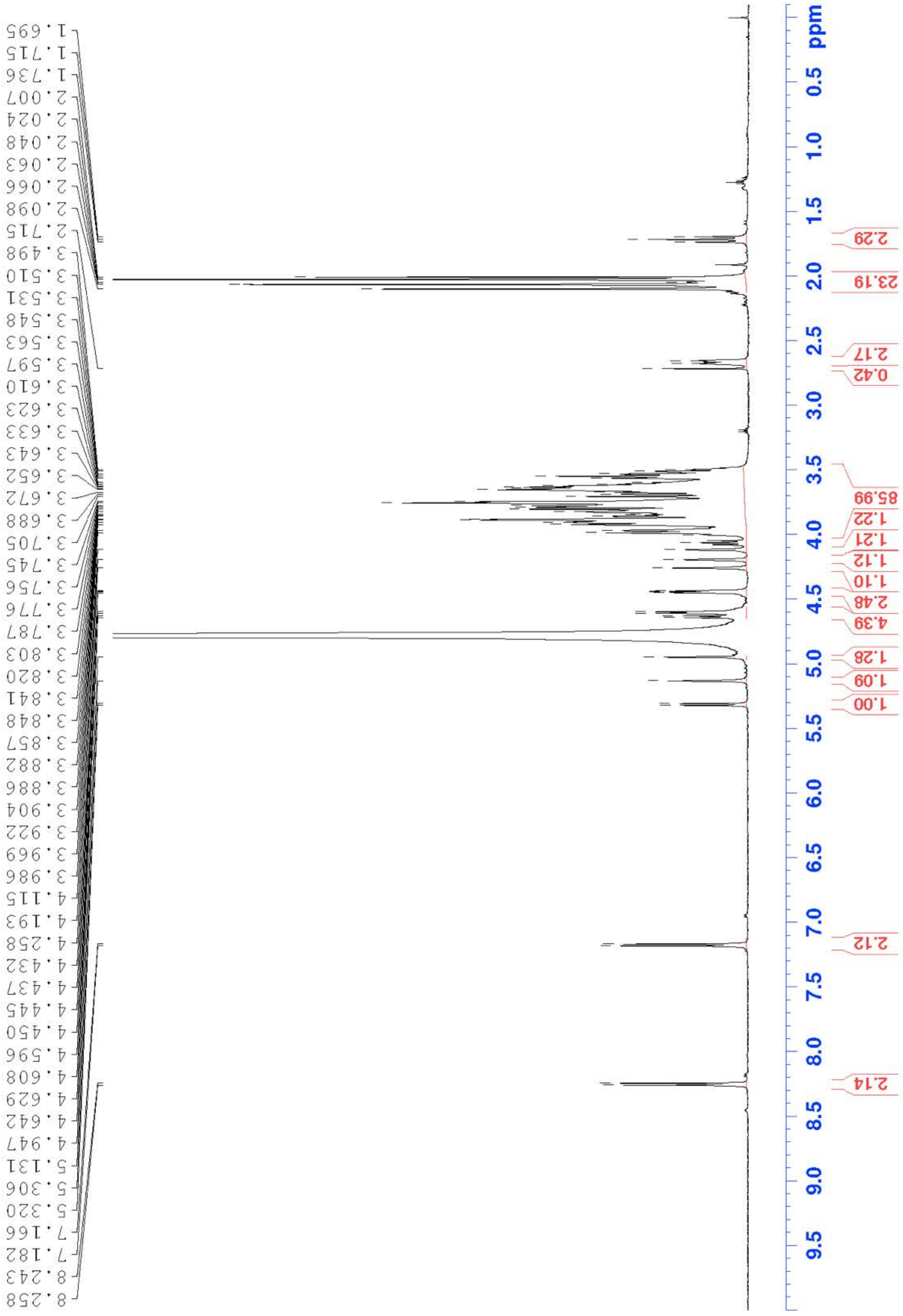

^{13}C-NMR spectrum of compound **3**

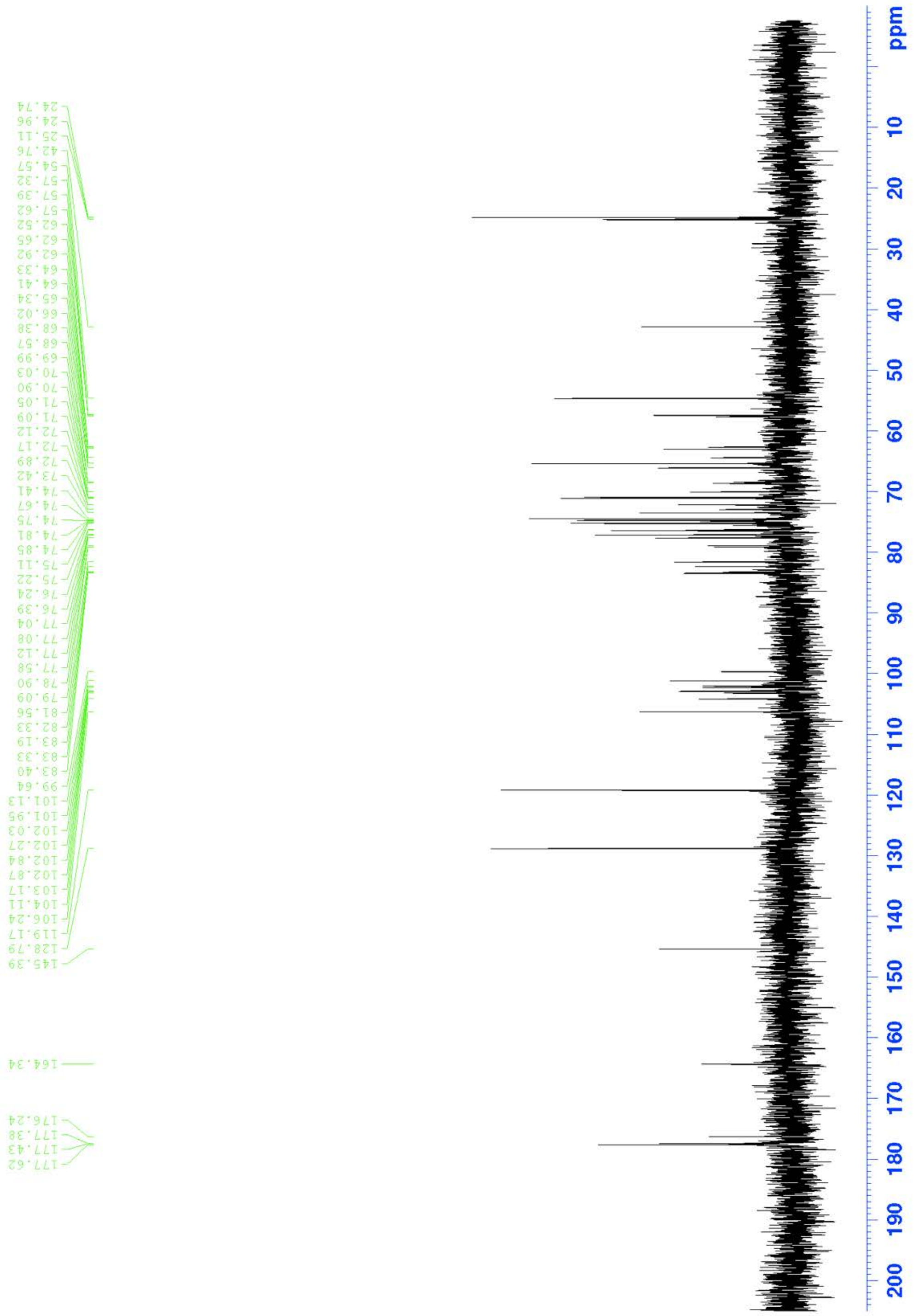

DQF COSY spectrum of compound **3**

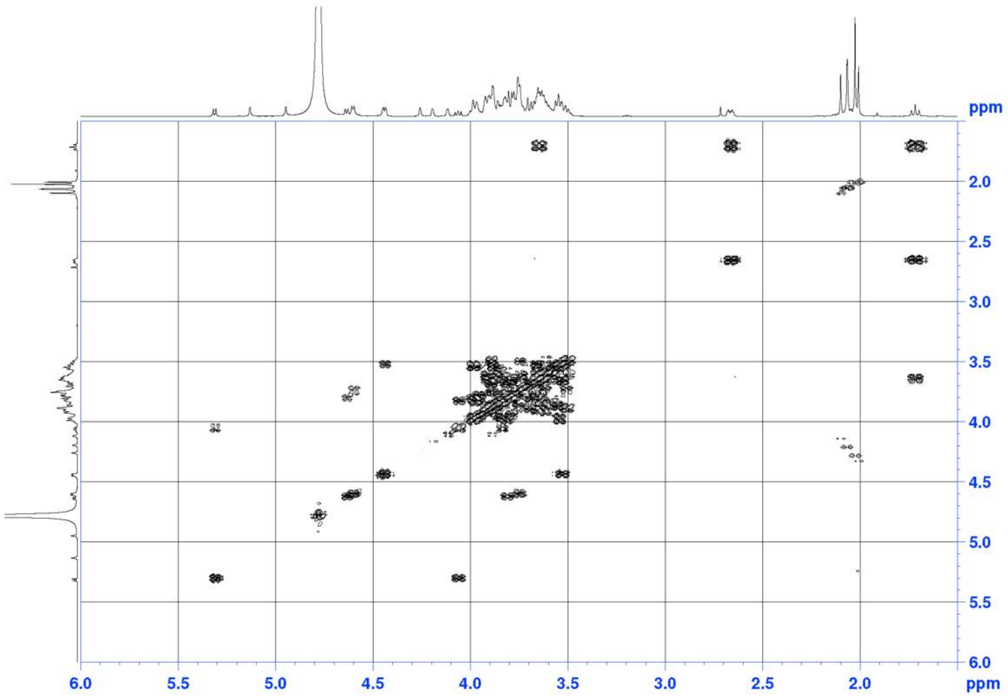

^{1}H-^{13}C HSQC spectrum of compound **3**

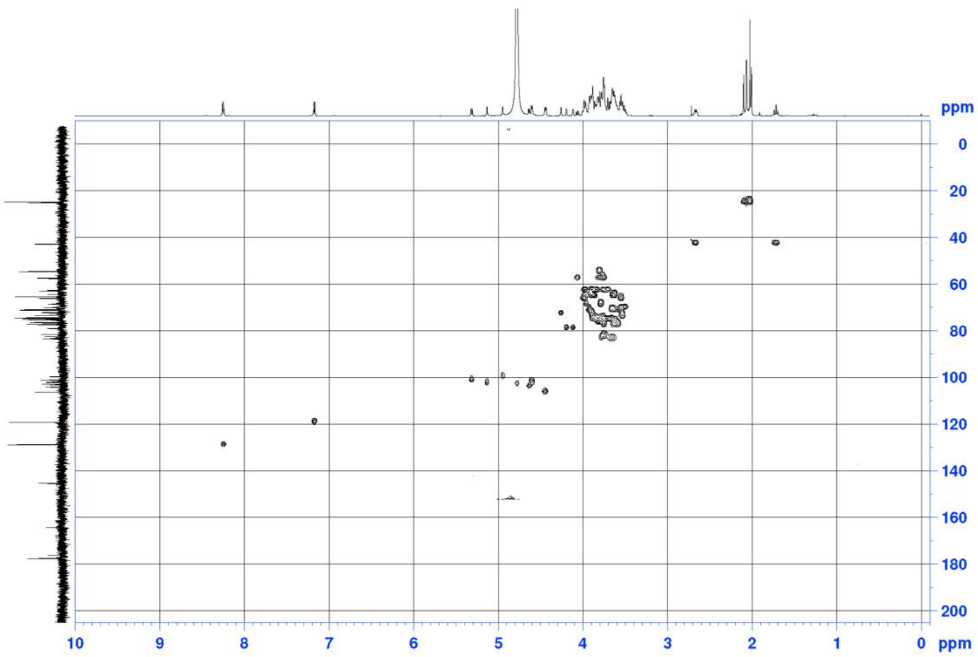

¹H-NMR spectrum of compound **4b**

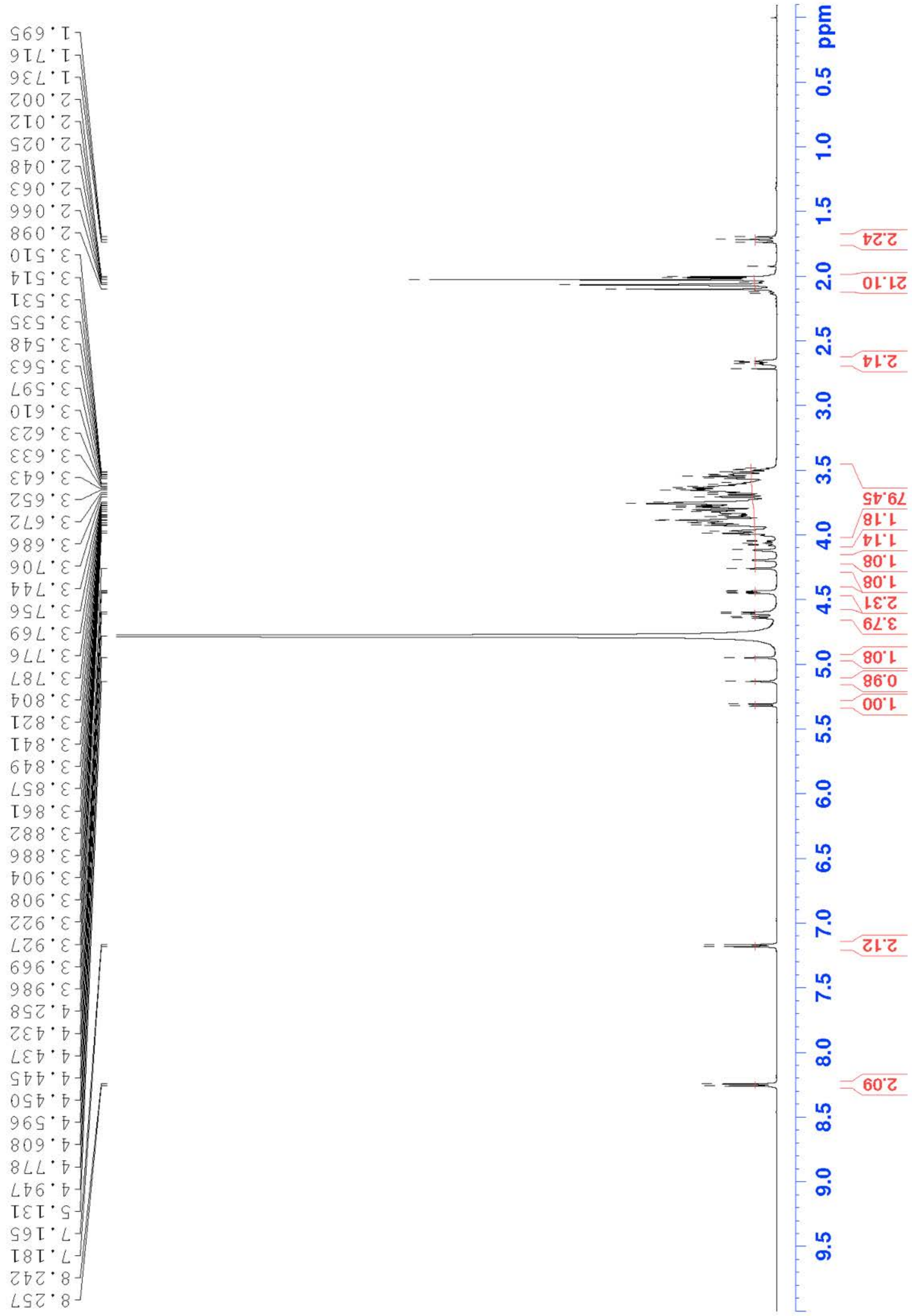

^{13}C-NMR spectrum of compound **4b**

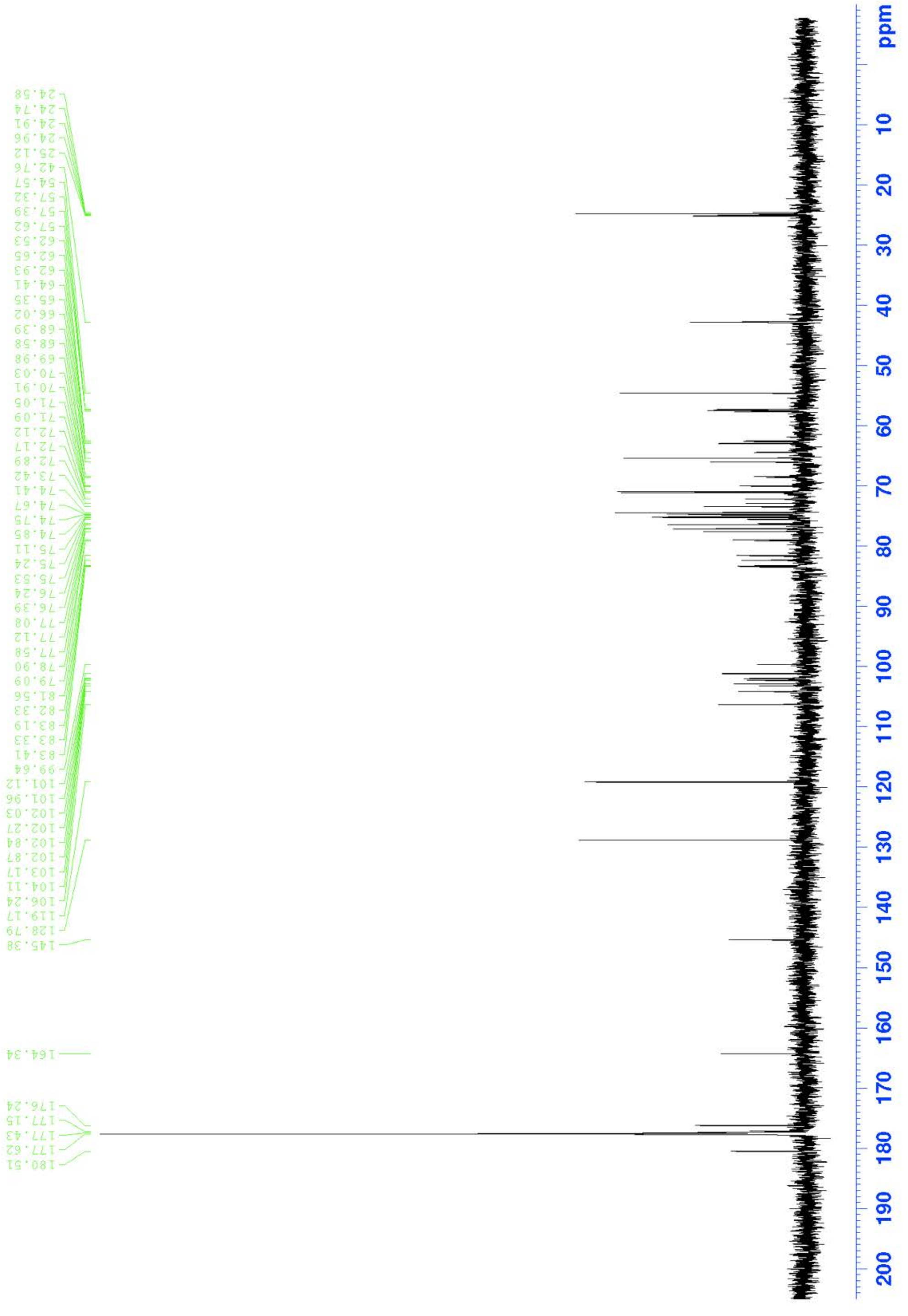

DQF COSY spectrum of compound **4b**

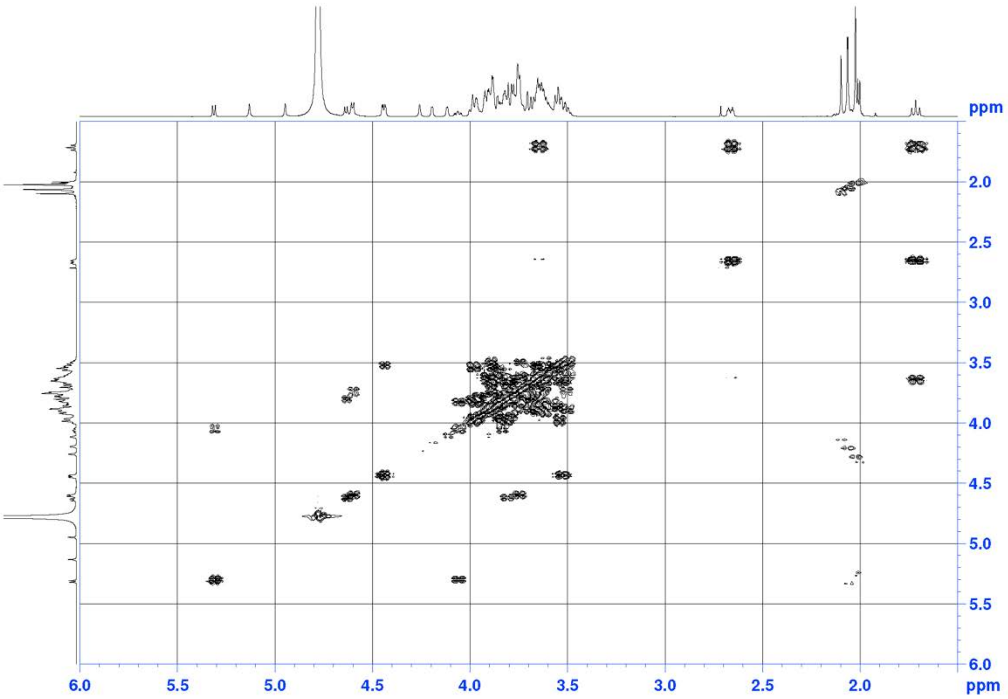

^{1}H-^{13}C HSQC spectrum of compound **4b**

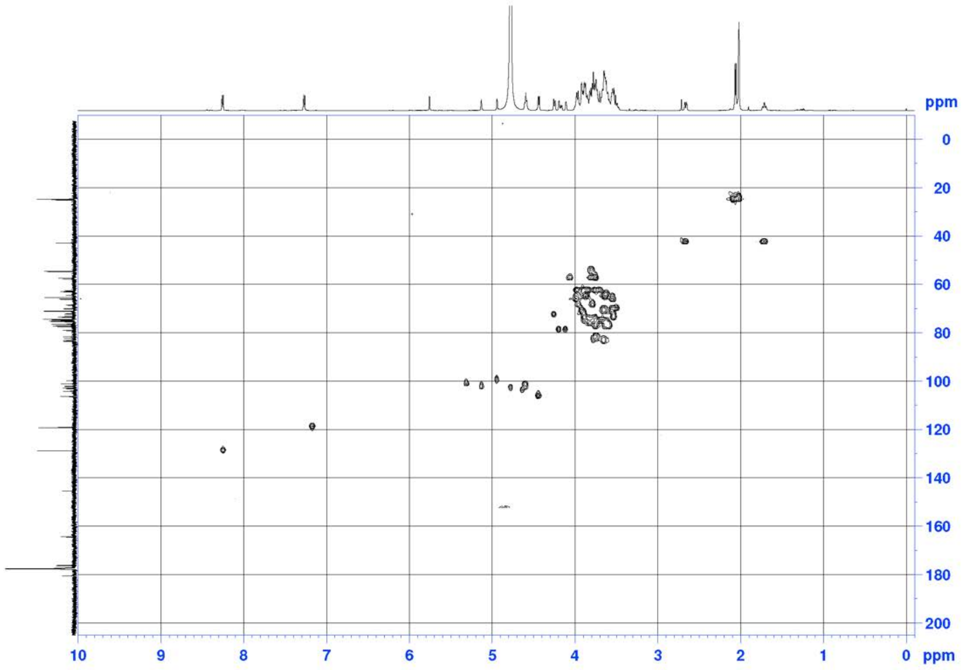

¹H-NMR spectrum of compound **5b**

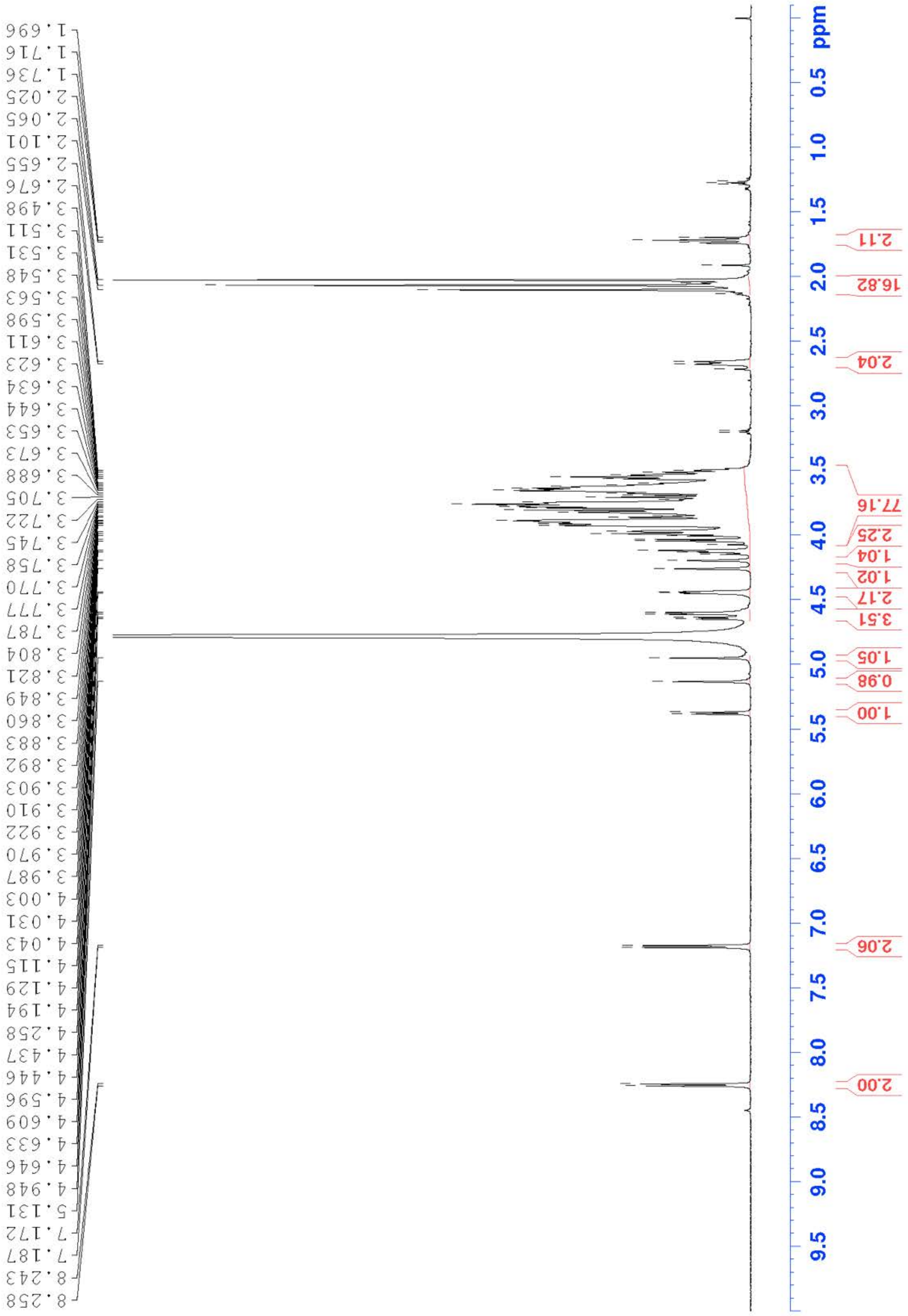

^{13}C-NMR spectrum of compound **5b**

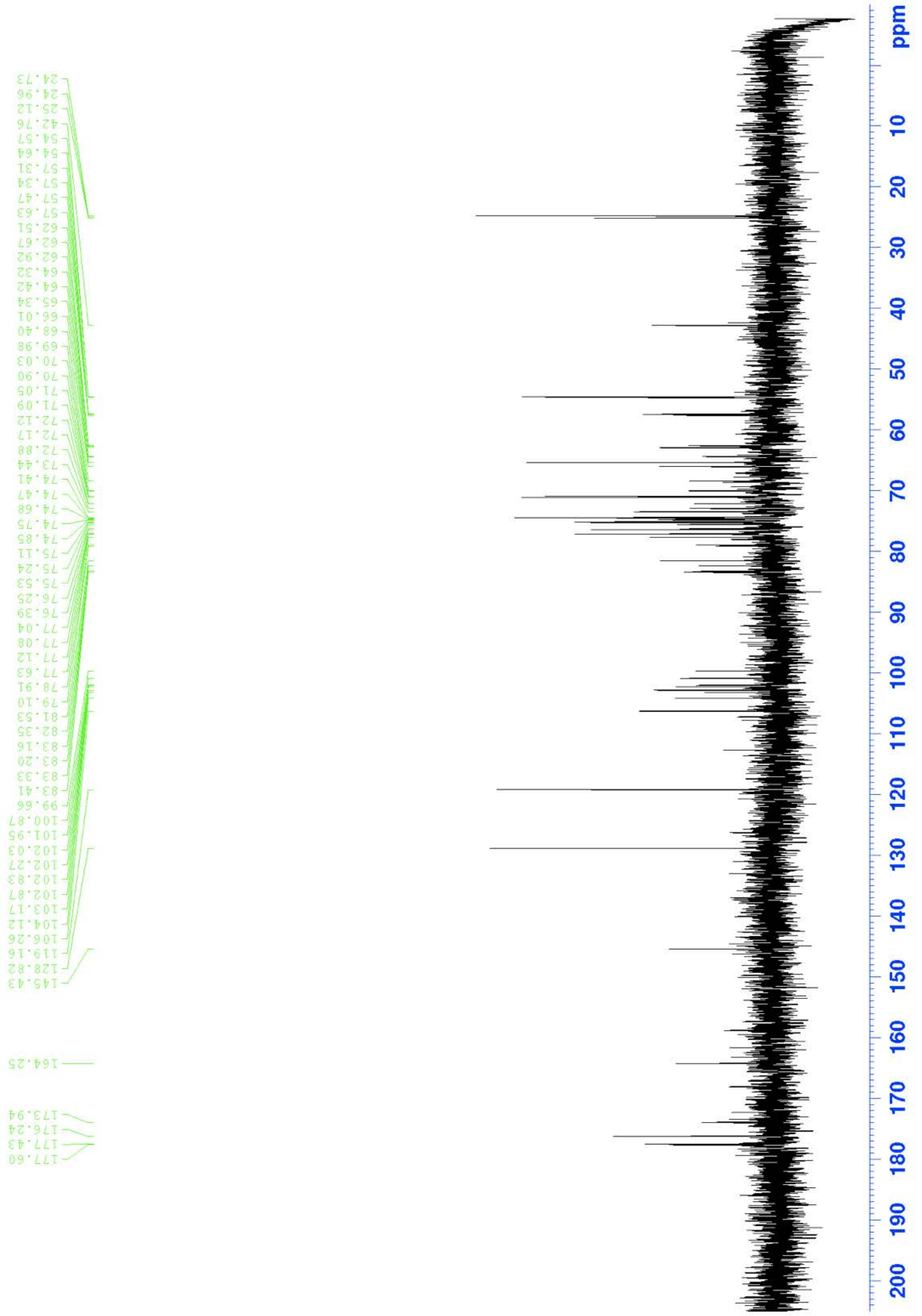

DQF COSY spectrum of compound **5b**

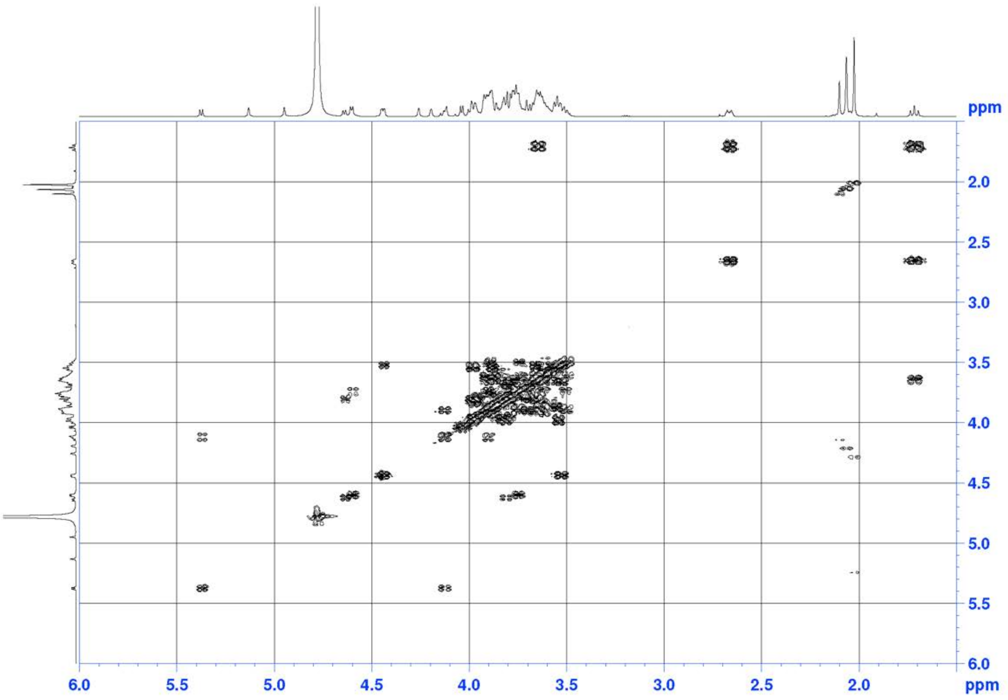

^{1}H-^{13}C HSQC spectrum of compound **5b**

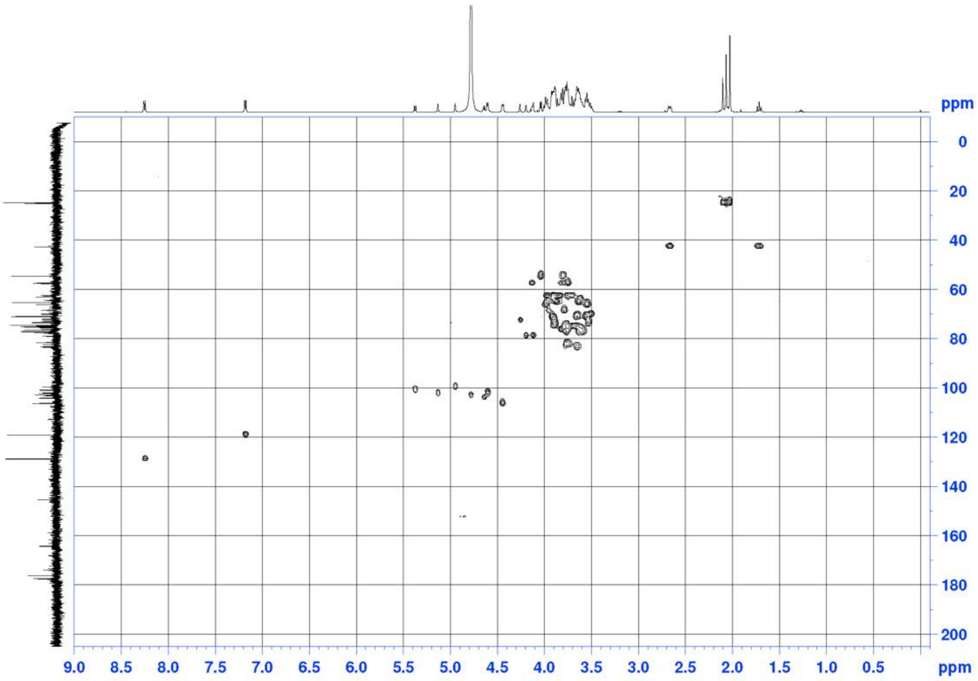

^{1}H-NMR spectrum of compound **6b**

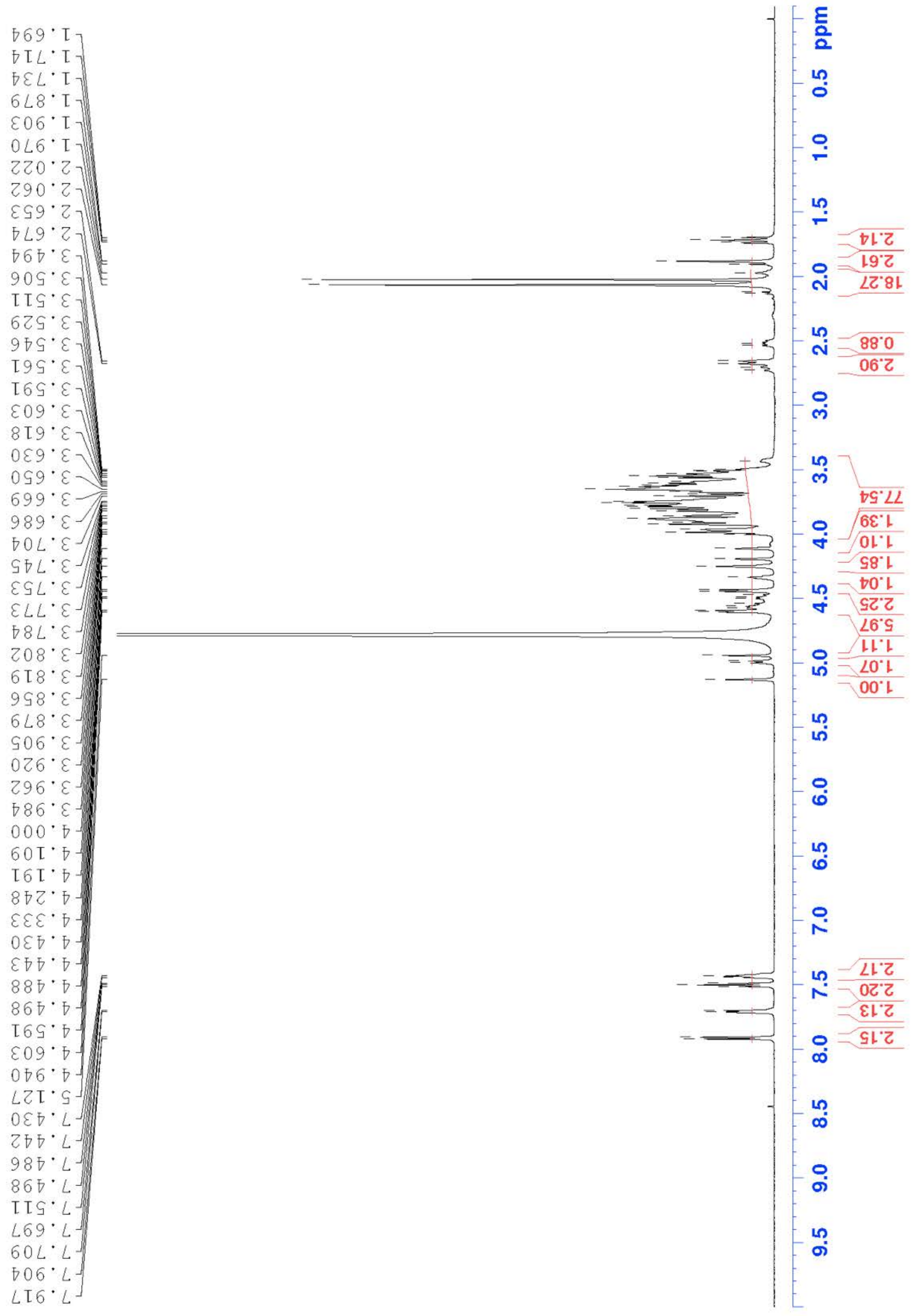

^{13}C-NMR spectrum of compound **6b**

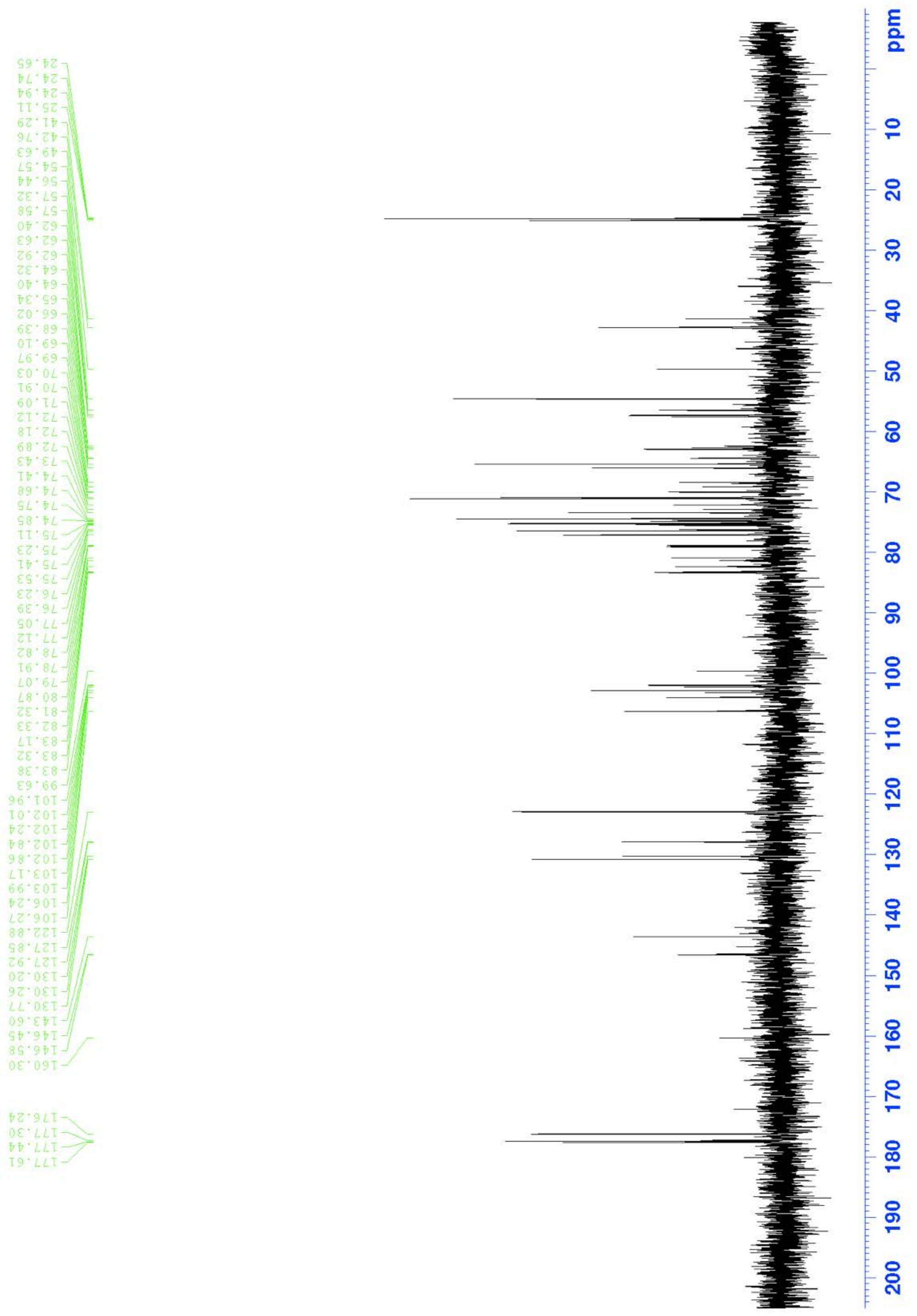

DQF COSY spectrum of compound **6b**

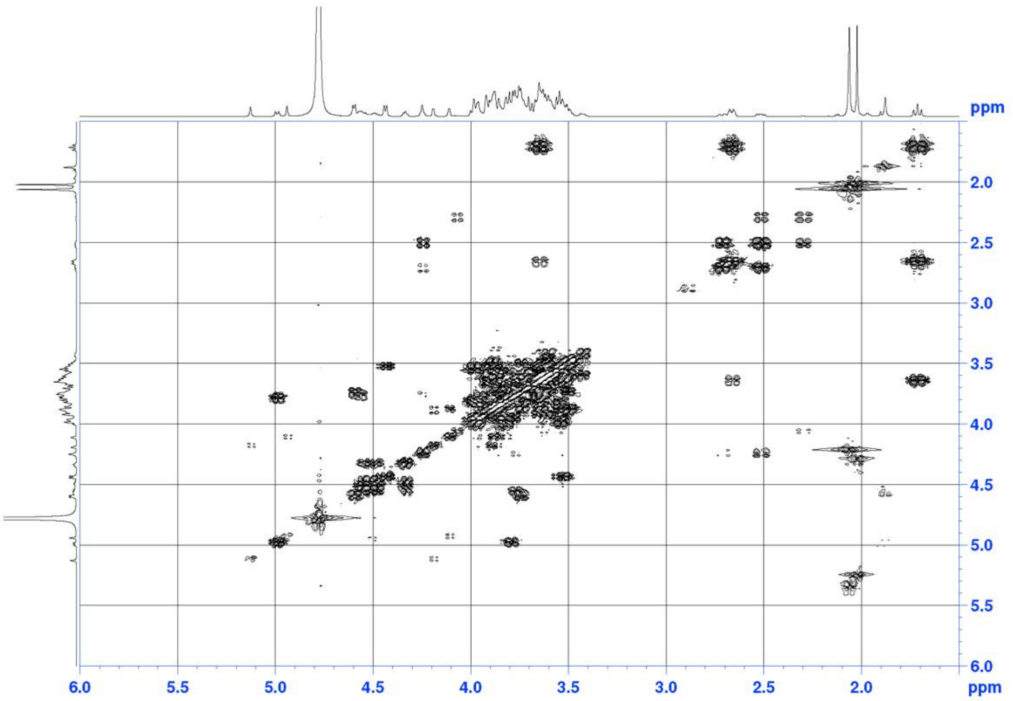

HSQC spectrum of compound **6b**

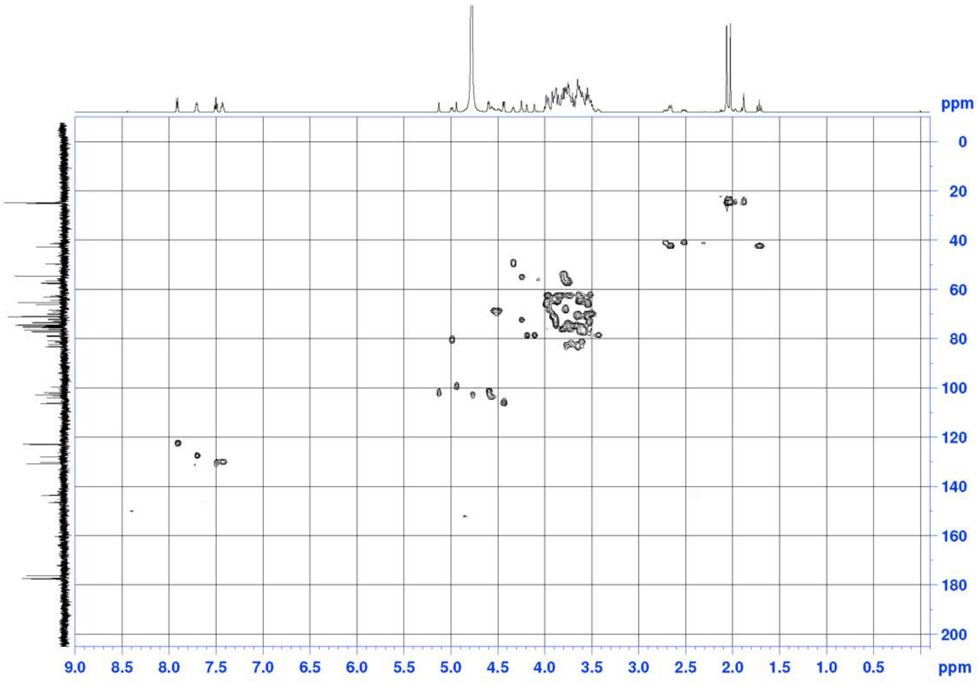

^{1}H-NMR spectrum of compound **7b**

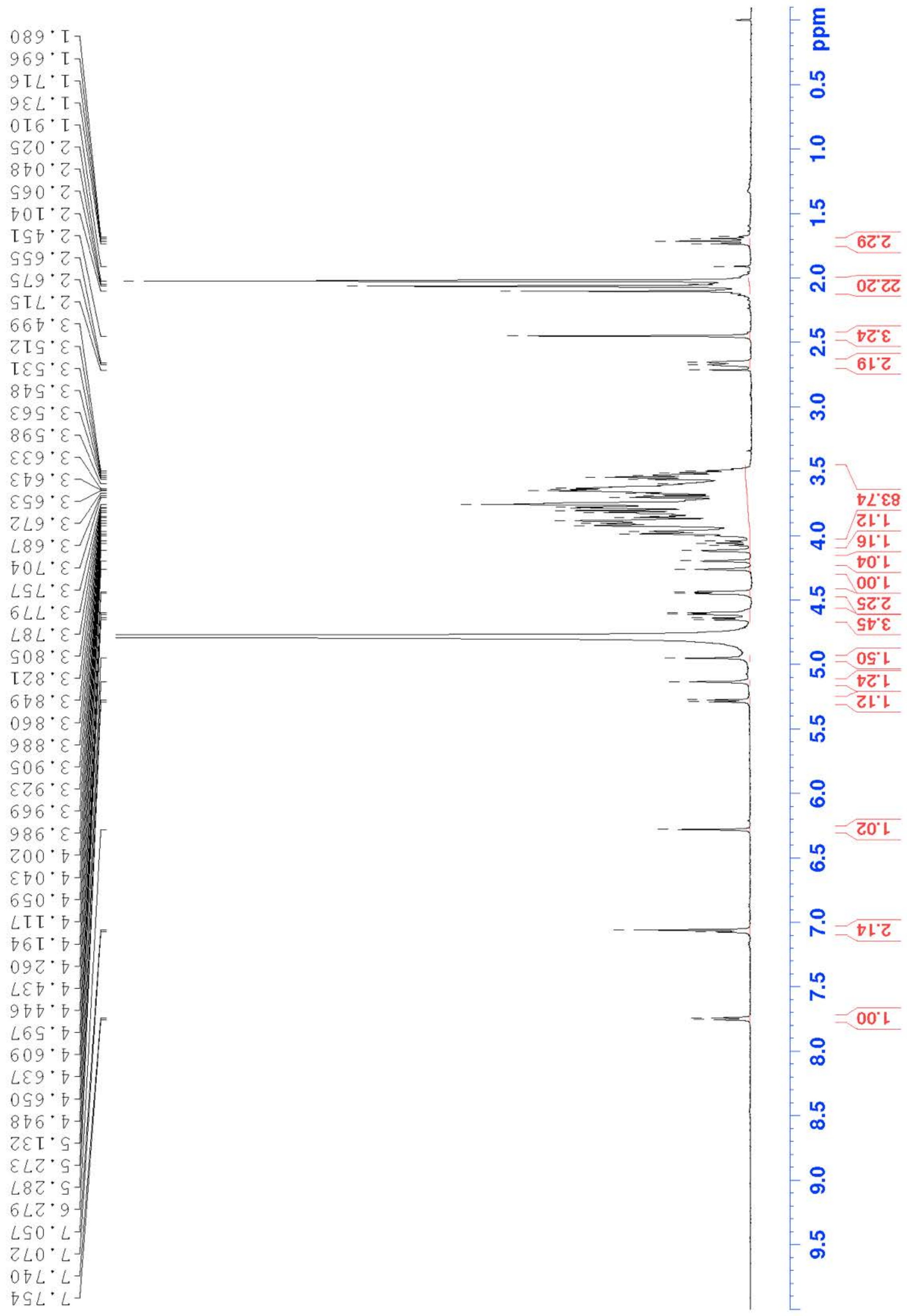

^{13}C-NMR spectrum of compound **7b**

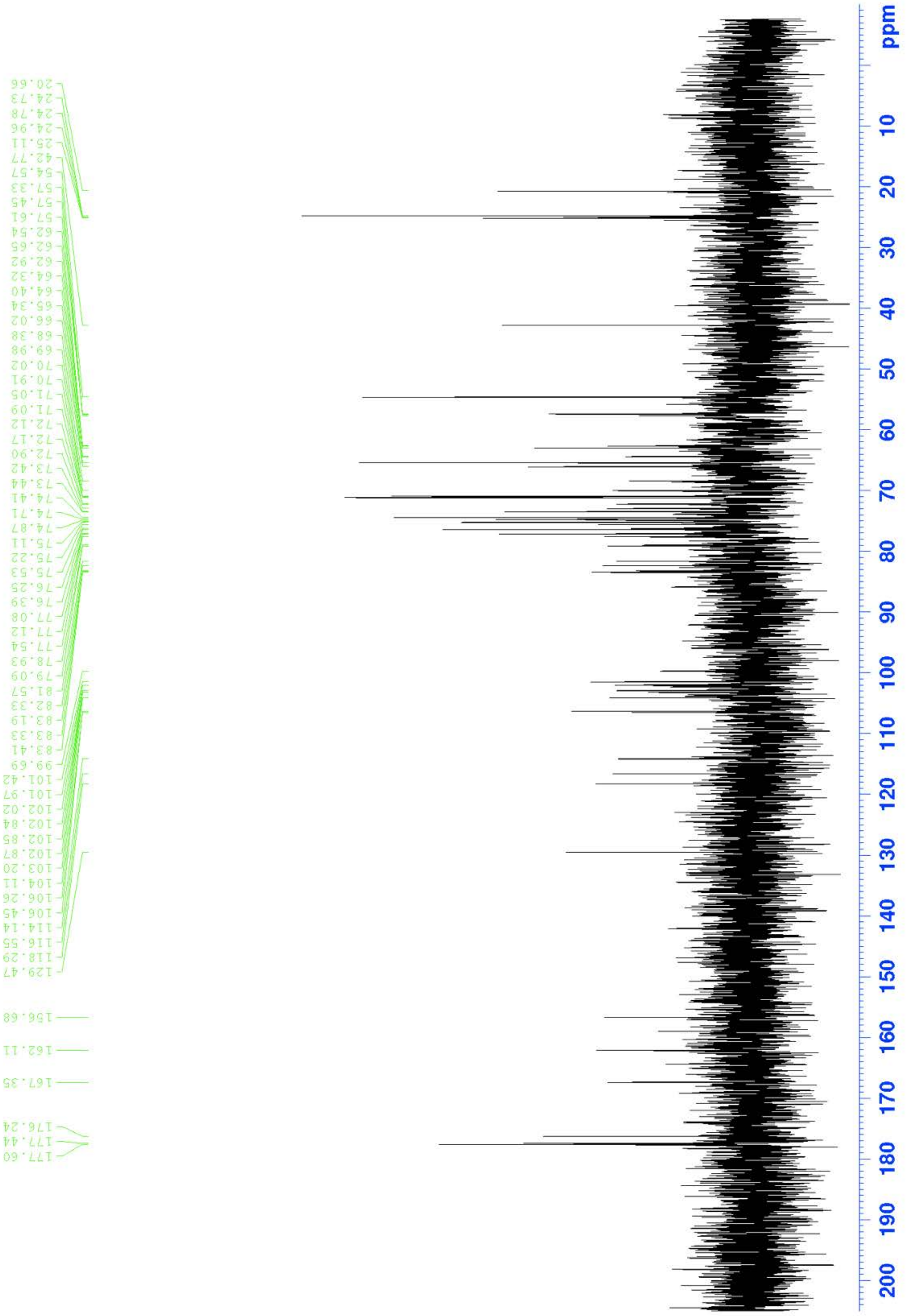

DQF COSY spectrum of compound **7b**

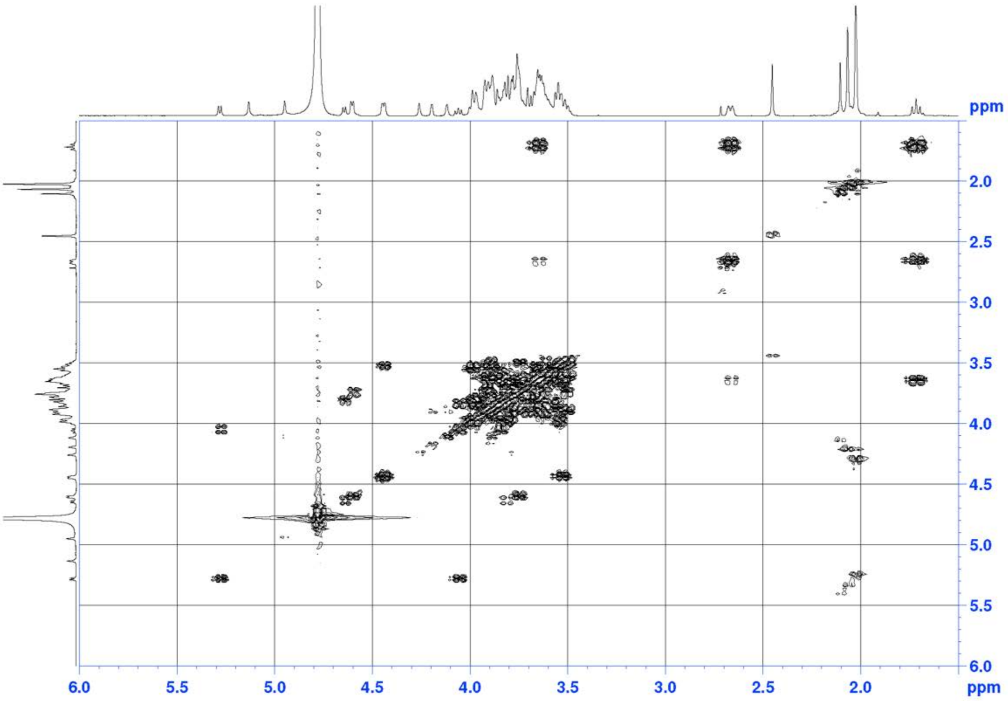

^{1}H-^{13}C HSQC spectrum of compound **7b**

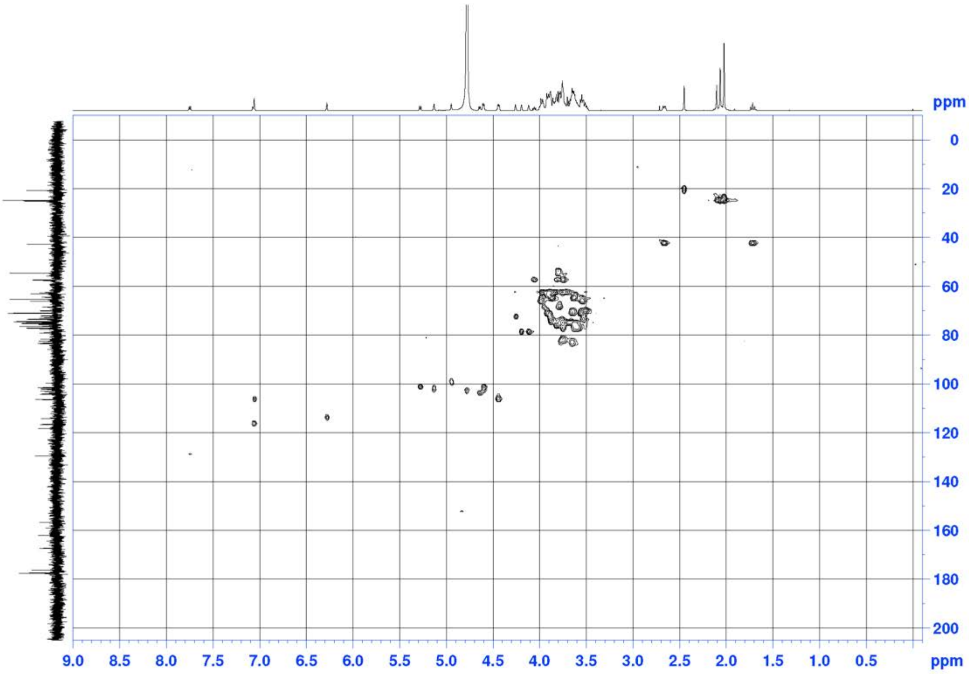

1H-NMR spectrum of compound **8b**

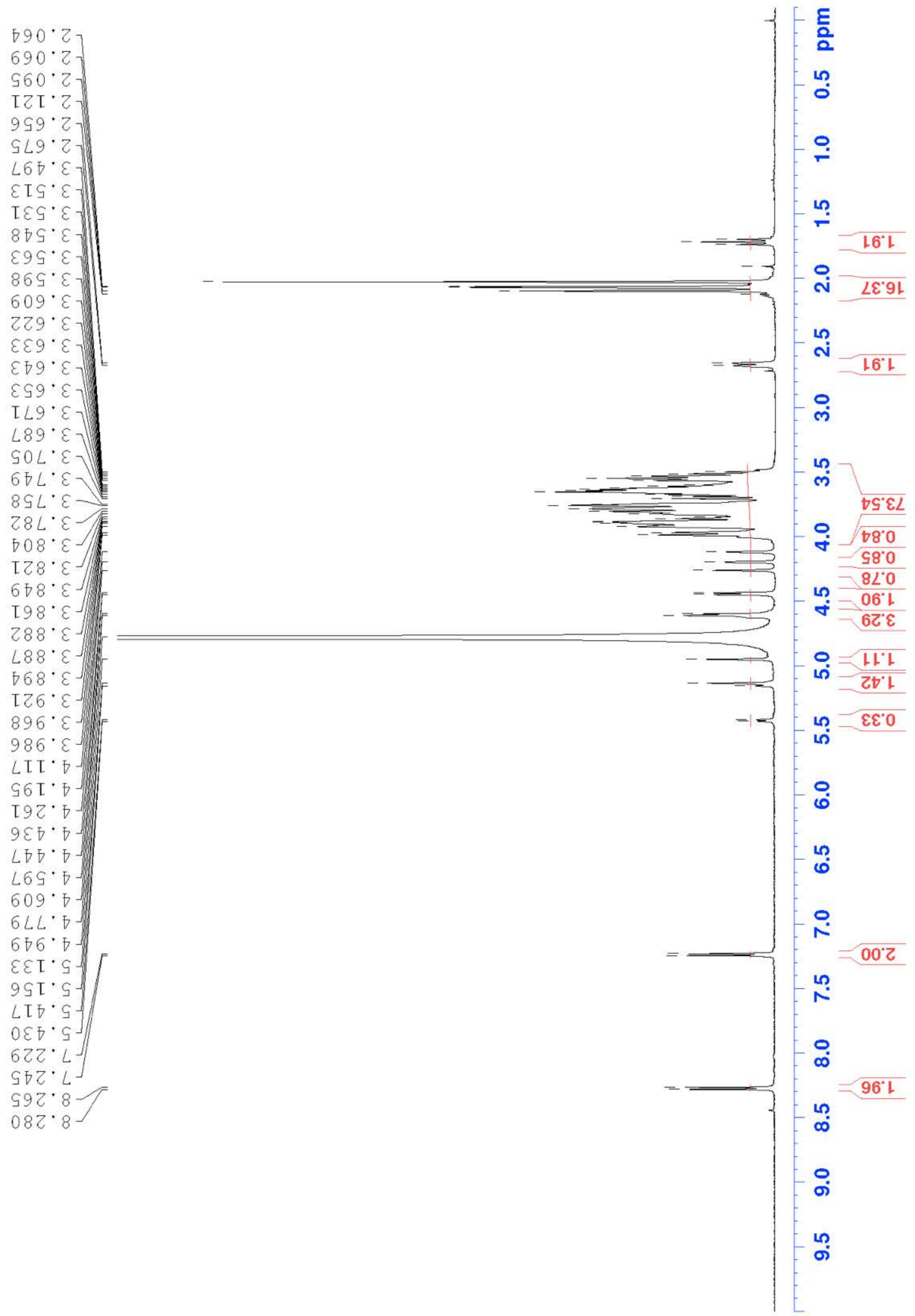

^{13}C-NMR spectrum of compound **8b**

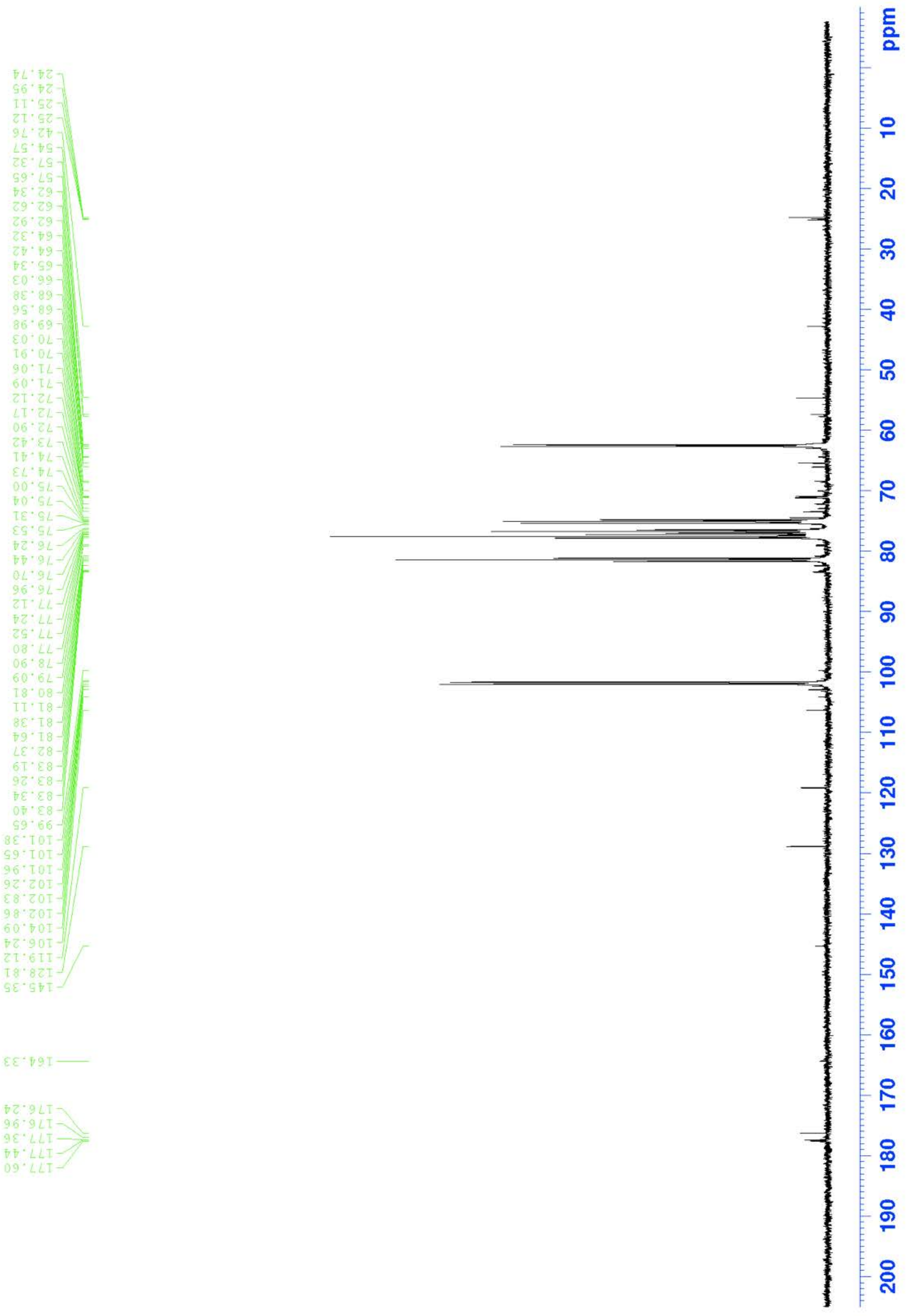

DQF COSY spectrum of compound **8b**

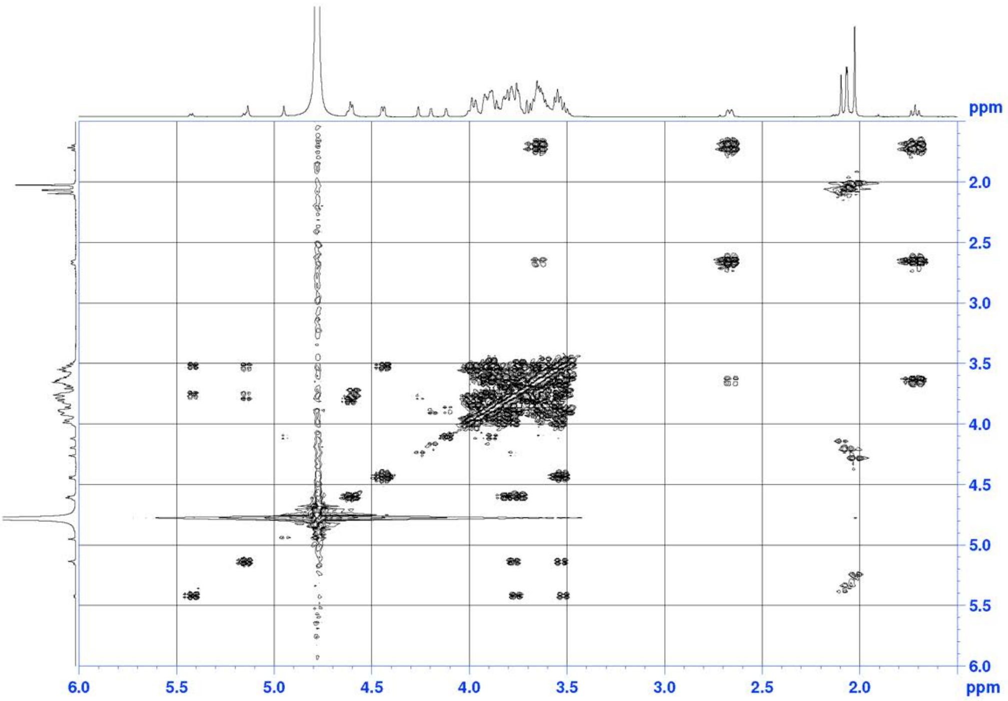

^{1}H-^{13}C HSQC spectrum of compound **8b**

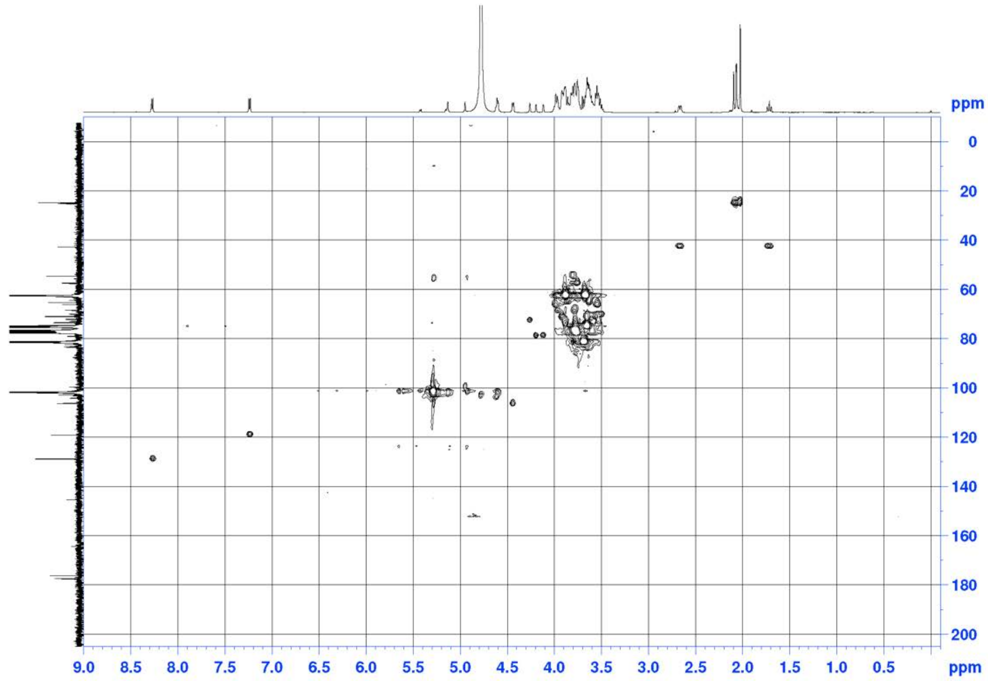

^{1}H-NMR spectrum of compound **9b**

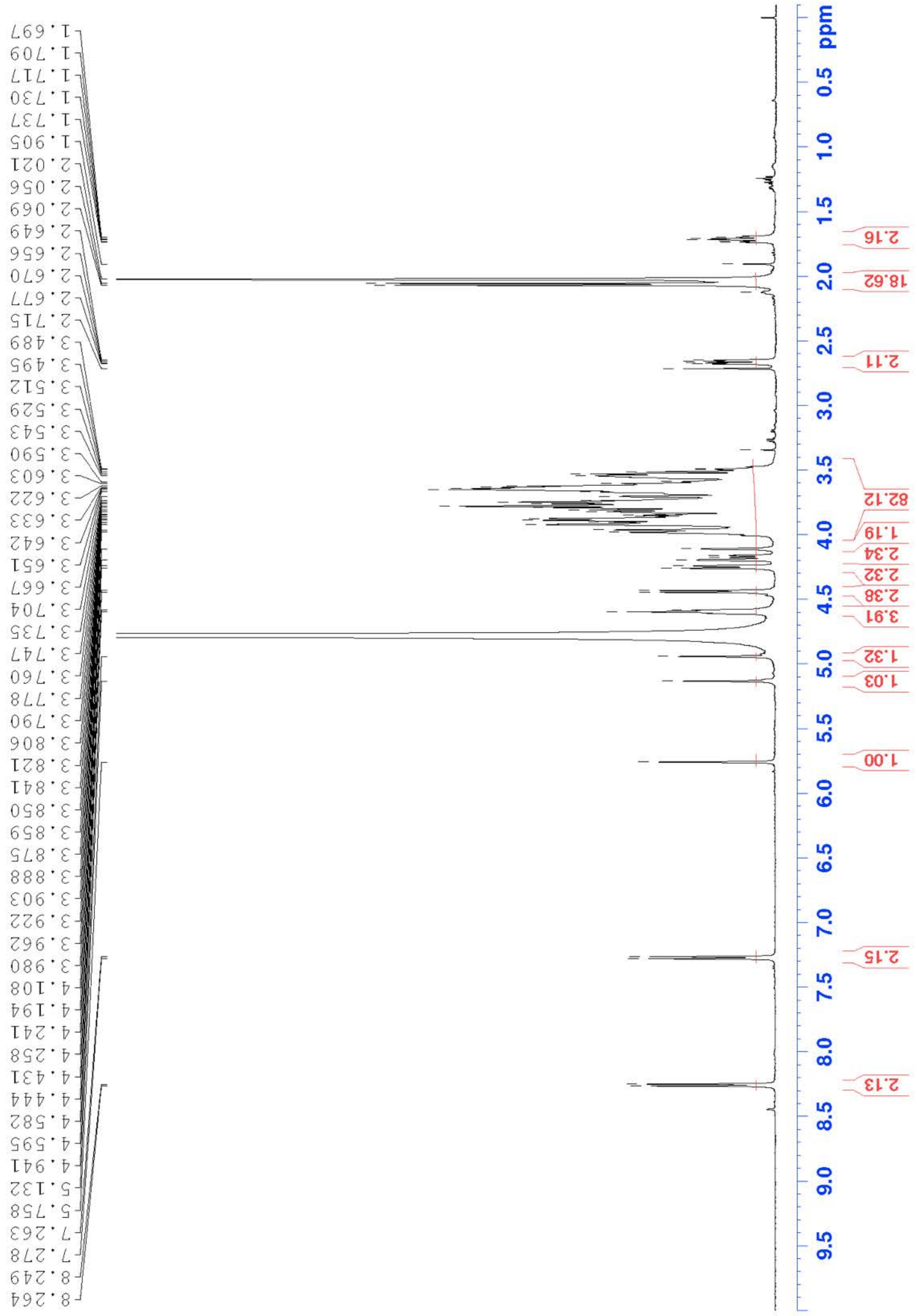

^{13}C-NMR spectrum of compound **9b**

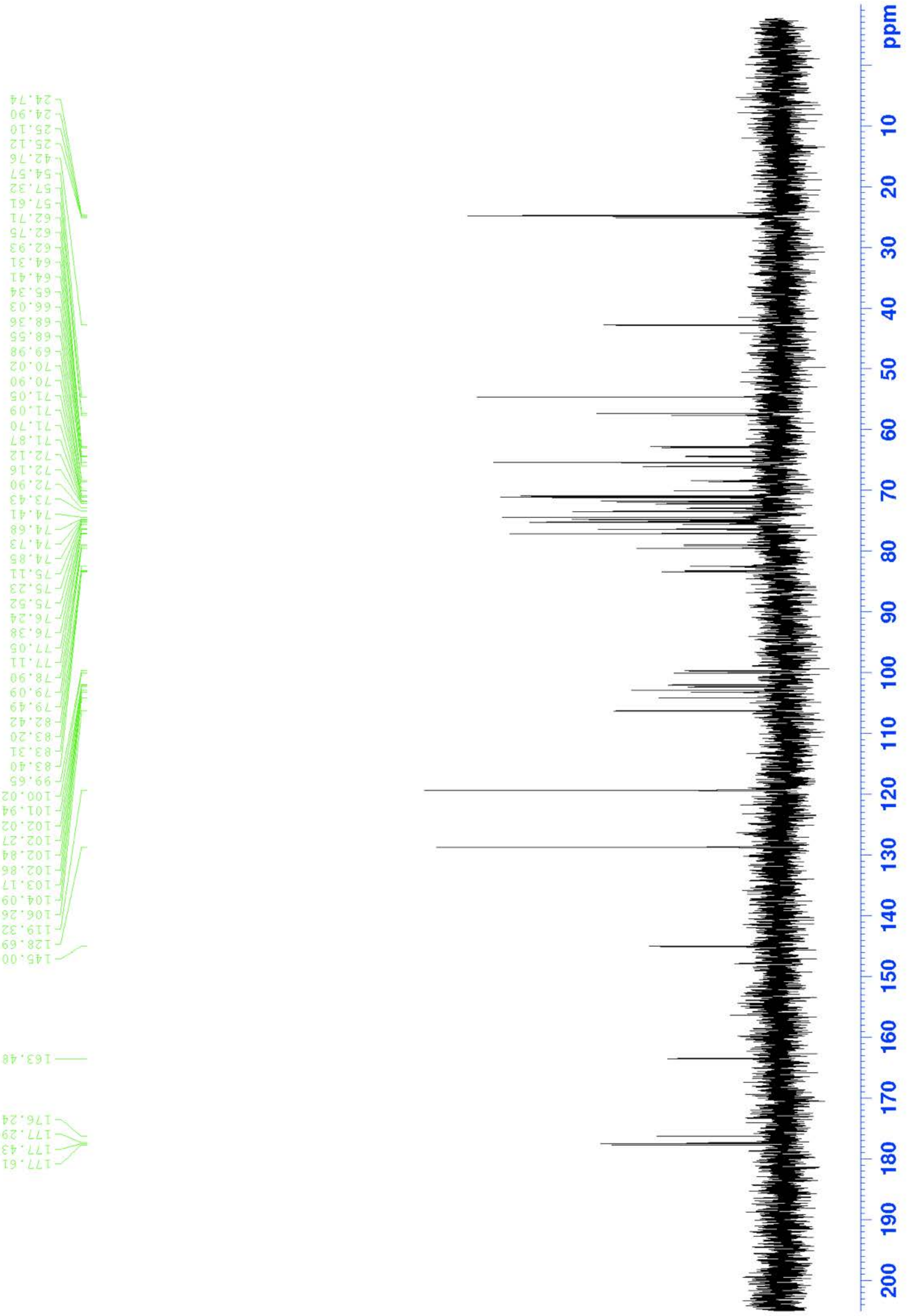

DQF COSY spectrum of compound **9b**

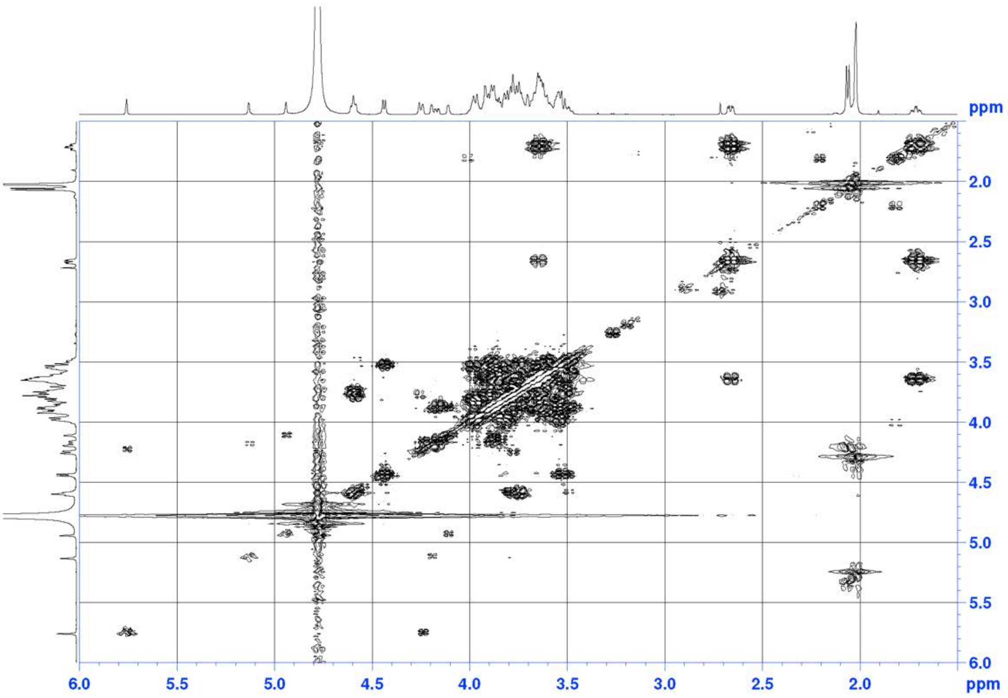

^{1}H-^{13}C HSQC spectrum of compound **9b**

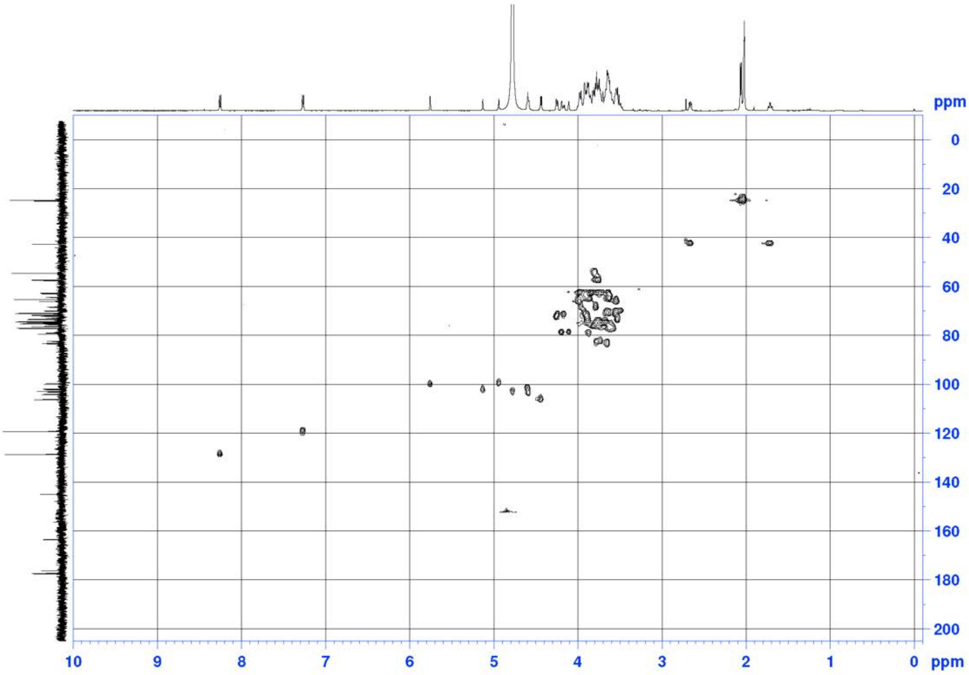

^{1}H-NMR spectrum of compound **15b**

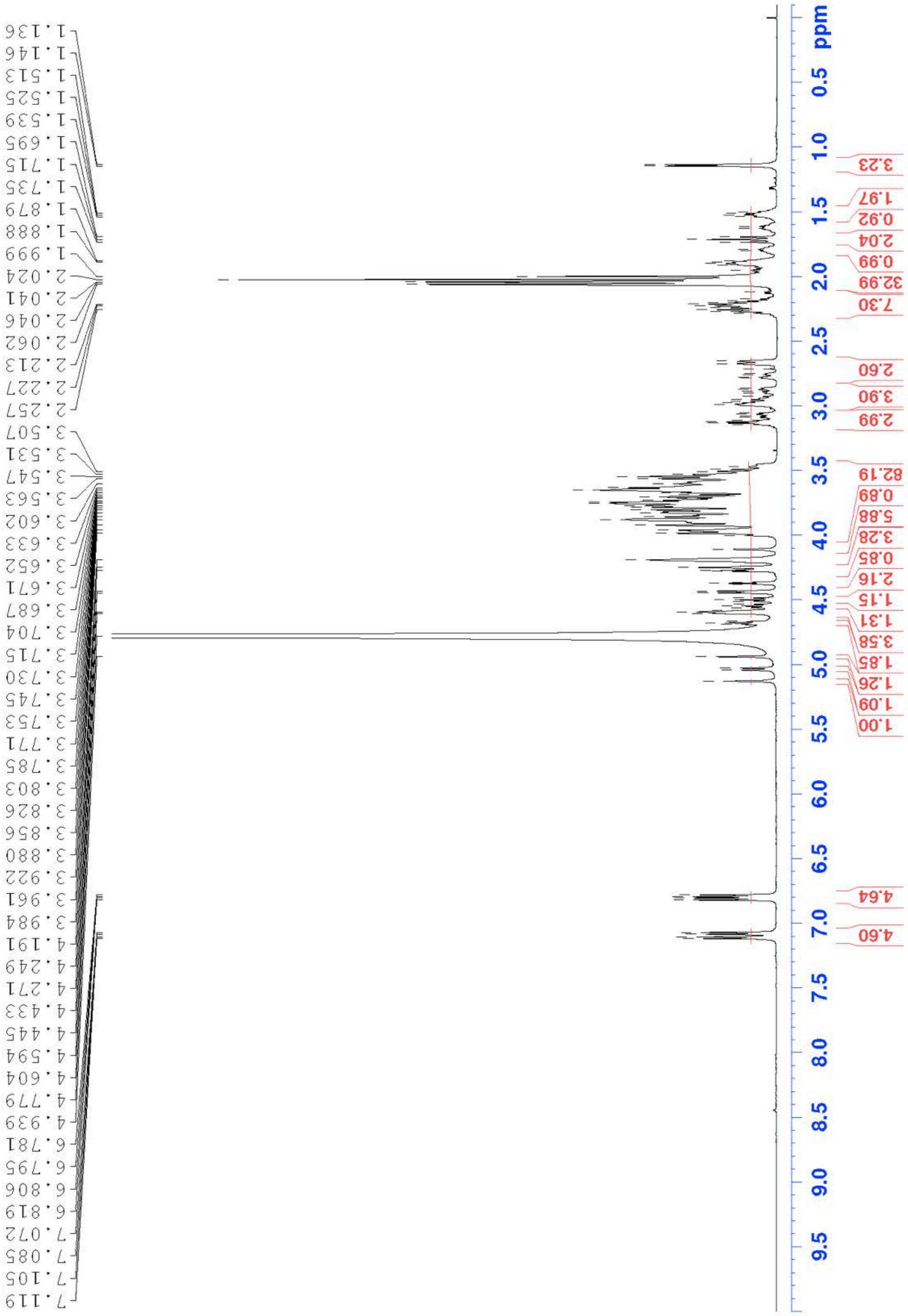

^{13}C-NMR spectrum of compound **15b**

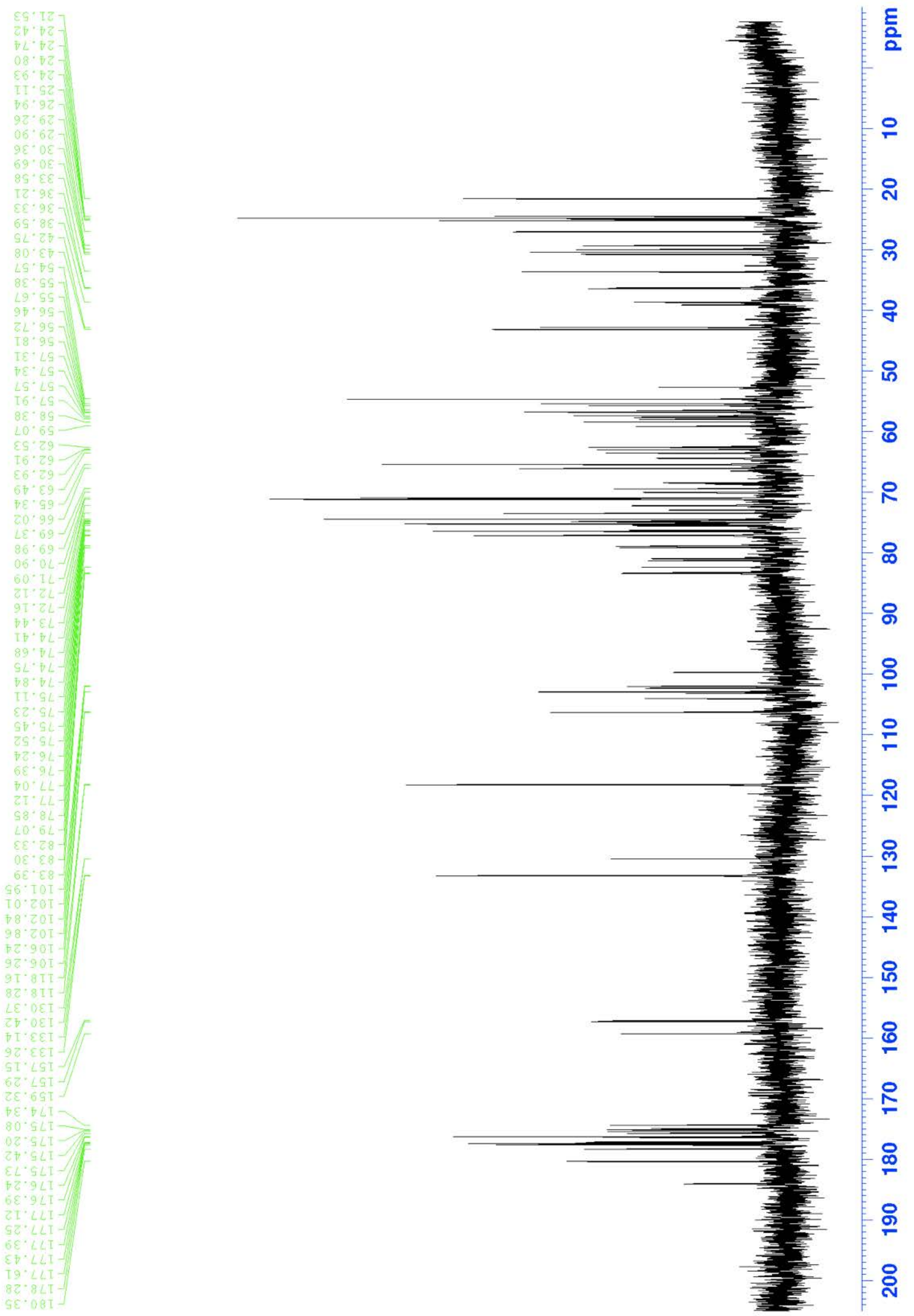

^{1}H-^{1}H COSY spectrum of compound **15b**

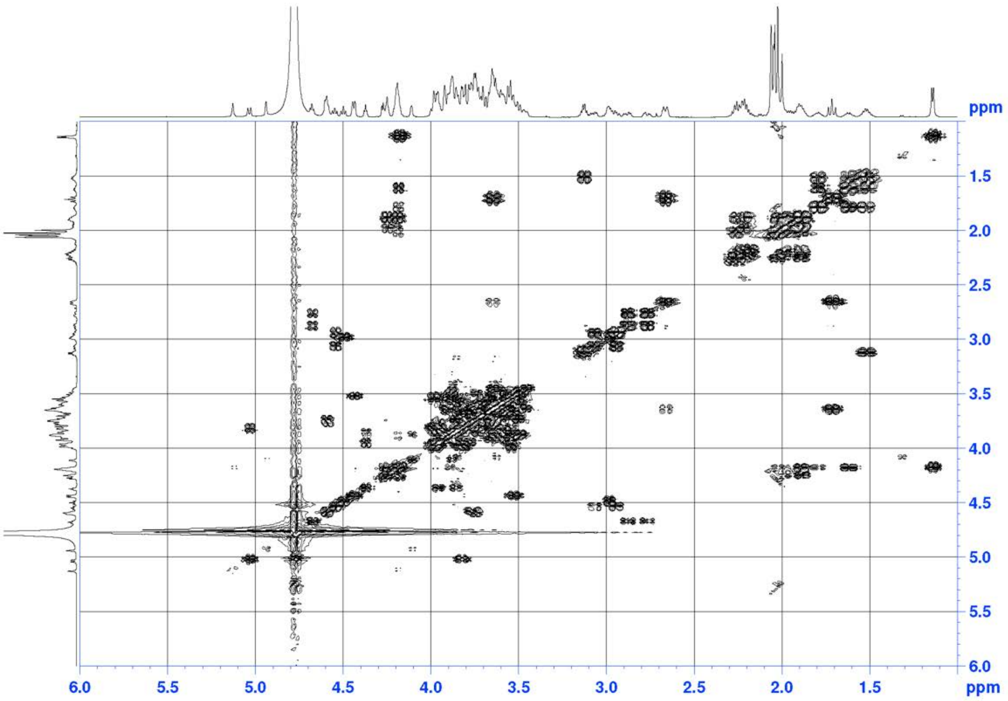

^{1}H-^{13}C HSQC spectrum of compound **15b**

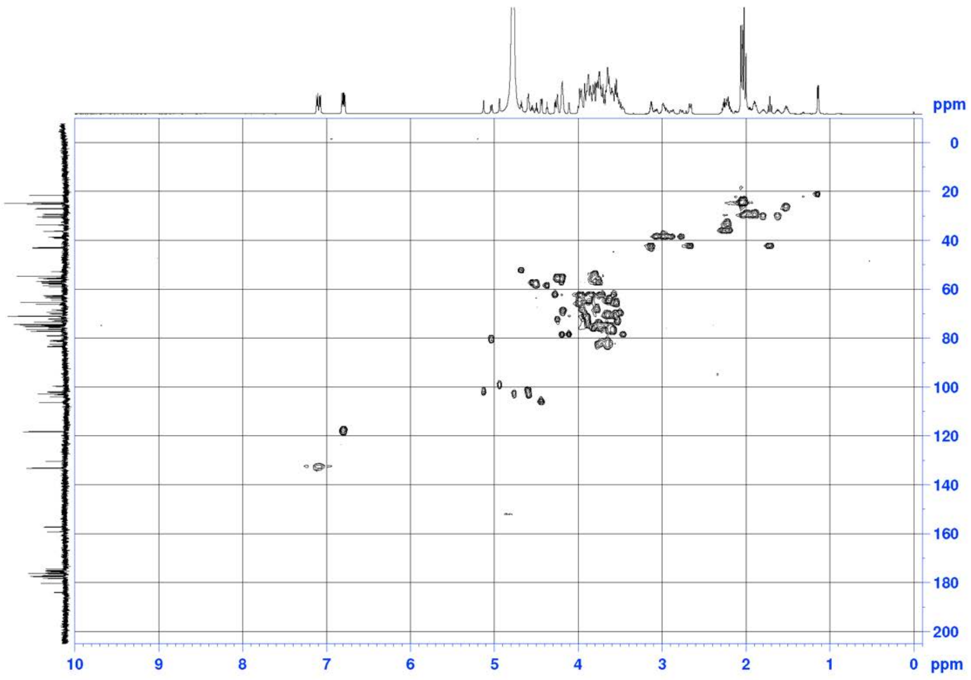

Supplement: Acceptor range of endo-β-N-acetylglucosaminidase Mutant endo-CC N180H: from Monosaccharide to Antibody: Supporting information [file rsos171521supp1.pdf]
